# Photocatalytic *In Situ* Activation of Bench-Stable Saccharides: A General Synthetic Strategy for *C*‑Glycosyl Bicyclo[1.1.1]pentanes

**DOI:** 10.1021/acs.orglett.6c02105

**Published:** 2026-06-08

**Authors:** Chun Qi, Luca Dell’Amico, Giulio Goti

**Affiliations:** Department of Chemical Sciences, 124236 University of Padova, Via Francesco Marzolo 1, 35131 Padova, Italy

## Abstract

*C*-Glycosyl bicyclo[1.1.1]­pentanes (BCPs) are saturated analogues of aryl *C*-glycosides; however, the available methods for their preparation remain limited in scope and generality. Herein, we develop a general strategy for their synthesis using bench-stable saccharides for the *in situ* generation of glycosyl iodides that undergo atom transfer radical addition (ATRA) to [1.1.1]­propellane. By avoiding the need for the isolation of activated glycosides, this approach enables the incorporation of C­(*sp* ^3^)-rich BCP motifs across both disarmed and armed glycosides.

## Graphical abstract

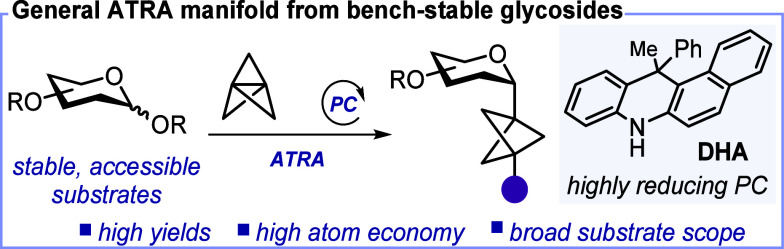

Alkyl *C*-glycosides are widespread in nature and have found successful application in the development of metabolically stable drugs.^1^ These molecules are commonly synthesized by forging a C–C bond at the anomeric position of glycosides, a task that can be achieved through multiple strategies,^2^ involving glycosyl anionic,^3^ cationic,^4^ and radical^5^ species and transition metal catalysts, among others (Figure 1A).^6^ While effective, all of these approaches are generally limited to the introduction of primary and secondary alkyl groups, with tertiary alkylation protocols remaining largely underdeveloped.

**Figure 1:**
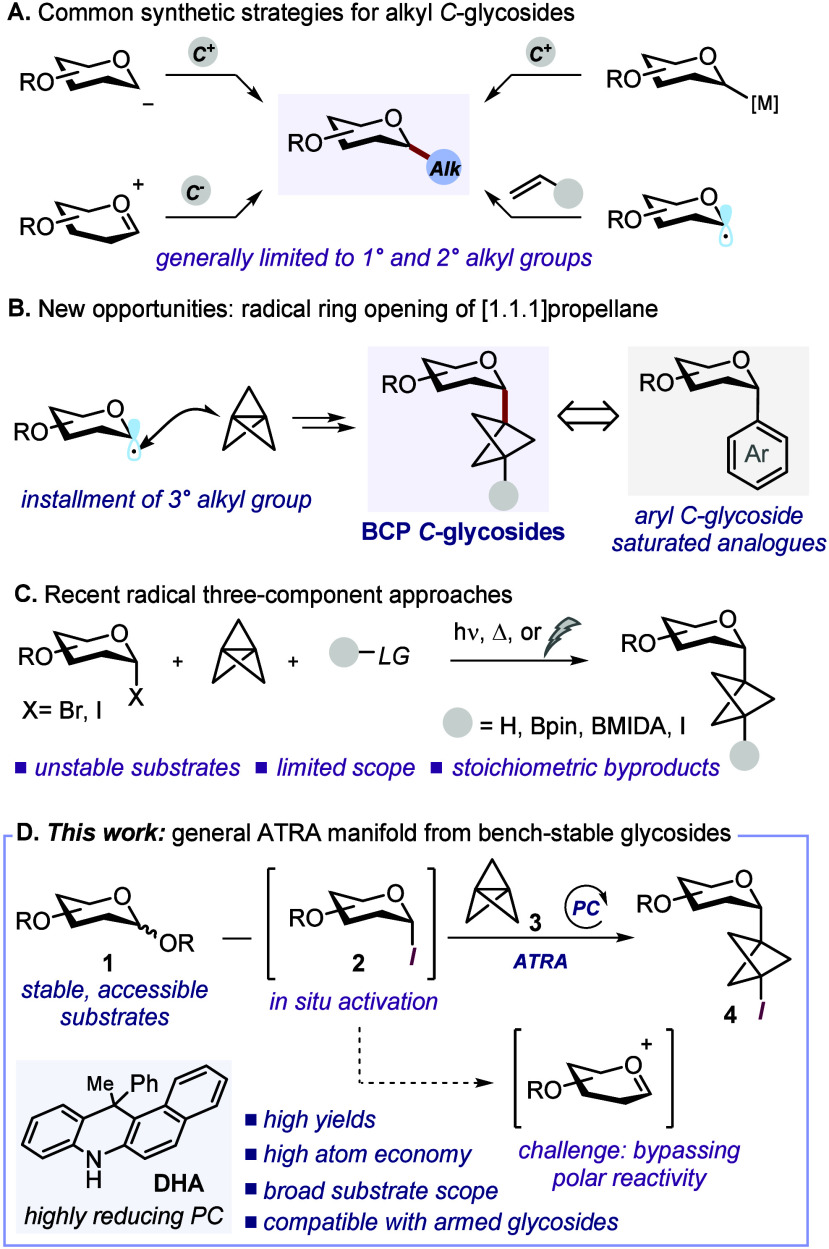
(A) Traditional approaches toward alkyl *C*-glycosides. (B) Radical strain-release strategy for C-glycosylation with a tertiary BCP moiety. (C) State-of-the-art methodology for the synthesis of *C*-glycosyl BCPs. (D) Our newly developed ATRA to [1.1.1]­propellane from bench-stable saccharides. Abbreviations: ATRA, atom transfer radical addition; BCP, bicyclo[1.1.1]­pentyl; LG, leaving group; pin, pinacol; MIDA, *N*-methyliminodiacetic acid.

The incorporation of saturated molecular fragments within drug scaffolds is a potent strategy in medicinal chemistry to access new chemical space and enhance physicochemical properties.^7^ Consequently, *C*-glycosylation methods enabling the introduction of highly substituted C­(*sp* ^3^) centers are in great demand.^8^ Of particular interest is the alkylation with three-dimensional bioisosteres of aryl rings to afford saturated analogues of aryl *C*-glycosides,^9^ a class of natural products and therapeutics of considerable clinical relevance.^10^ In this context, recent research efforts have focused on the incorporation of the bicyclo[1.1.1]­pentyl (BCP) moiety to access the corresponding *C*-glycosyl BCPs (Figure 1B).^11^

From a synthetic perspective, *C*-glycosyl BCPs can be conveniently prepared through strain-release reactivity via the addition of glycosyl radicals to [1.1.1]­propellane.^12^ Following this strategy and building on work from the Molander group,^13^ the Ackermann group and our research group independently reported distinct light-driven, electrochemical, and thermal protocols for the conversion of glycosyl halides into *C*-glycosyl BCP derivatives (Figure 1C).^14^ Although effective, all of these methods rely on unstable glycosyl bromides or iodides, which have to be generally prepared from glycosyl esters. In addition, these protocols share a common three-component approach that inextricably leads to the stoichiometric formation of byproducts.

Following a mechanistically distinct approach, the Anderson group reported an Ir-photocatalyzed protocol in which a per-acetylated d-glucosyl iodide derivative could undergo atom transfer radical addition (ATRA) with [1.1.1]­propellane to give an iodo BCP *C*-glycoside.^12g^ While the study investigated a broad range of alkyl iodides, general application of the method to the synthesis of *C*-glycosides is complicated by the high reactivity and instability of glycosyl iodides.^15^ These compounds have found limited application in synthesis since their preparation, handling, and isolation are challenging.^16^ Only disarmed derivatives (usually per-acetylated ones) are sufficiently stable to be isolated and characterized.^17^ To the best of our knowledge, glycosyl iodides are not even available on the market.^18^

To address these limitations, we pursued a general and operationally simple strategy enabling the direct conversion of a great variety of bench-stable, unactivated saccharides **1** into BCP *C*-glycosides **4**. Specifically, we envisaged that glycosyl iodides **2** could be generated *in situ* and reacted with [1.1.1]­propellane **3** under photocatalytic conditions, thereby eliminating the need for the isolation and handling of inherently unstable glycosyl halides (Figure 1D). For this strategy to succeed, the highly reactive glycosyl iodides must undergo rapid ATRA, avoiding competing polar pathways. This is particularly critical for armed glycosides bearing electron-donating protecting groups, which favor oxocarbenium ion formation.^19^ We therefore hypothesized that the dihydrobenzoacridine (**DHA**) photoredox catalyst developed in our laboratory could efficiently activate **2** to generate glycosyl radicals. Indeed, **DHA** exhibits a sufficiently reducing excited-state potential (*E*_ox_*­(**DHA** ^**•**^^+^/DHA*) = −2.37 V vs SCE in CH_3_CN)^20^ to promote reductive cleavage of the C–I bond in otherwise difficult-to-reduce glycosyl iodides (*E*_red_(**1a**/**1a** ^•–^) = −1.94 V vs SCE in CH_3_CN (see the Supporting Information)), thereby effectively initiating a chain mechanism (*vide infra*).

To test the feasibility of our idea, we started investigating the reaction between β-d-glucose pentaacetate **1a** and Me_3_SiI as an activating agent at 0 °C,^21^ followed by the one-pot addition of [1.1.1]­propellane **3** and **DHA** as the photocatalyst under 427 nm LED irradiation (Table 1). Initially, CH_2_Cl_2_ was identified as an effective solvent for the *in situ* generation of glucosyl iodide from **1a**, resulting in **4a** predominantly as the α-anomer (73% yield, 6:1 α:β ratio (see Table 1, entry 1)). The reaction showed a strong dependence on the stoichiometry of Me_3_SiI, with optimal performance observed at 1.2 equiv (Table 1, entries 1–4). Using 1,2-dichloroethane as the solvent increased the yield to >95% (Table 1, entry 5) by reducing the extent of formation of hydrodehalogenated side products, likely arising from unwanted hydrogen atom transfer (HAT) from the solvent. Finally, control experiments revealed that the process occurs sluggishly in the dark with reduced efficiency in the absence of the photocatalyst under 390 or 427 nm irradiation (Table 1, entries 6–8). Notably, *fac*-Ir­(ppy)_3_ failed to promote the reactivity, highlighting the superior ability of **DHA** to sustain the radical chain (Table 1, entry 9). Interestingly, no conversion was observed upon mixing all of the reaction components together.

**Table 1:**
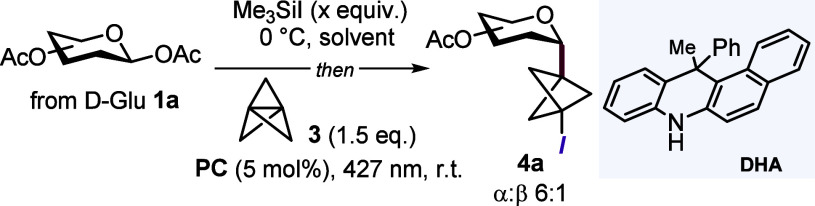
Optimization of the Reaction Conditions^a^

entry	Me_3_Sil (equiv)	solvent	PC	yield of **4a** (%)^b^
1	1.1	CH_2_Cl_2_	**DHA**	73
2	1.2	CH_2_Cl_2_	**DHA**	80
3	1.3	CH_2_Cl_2_	**DHA**	68
4	2.0	CH_2_Cl_2_	**DHA**	<5
5	1.2	DCE	**DHA**	>95
6^c^	1.2	DCE	–	32
7^d^	1.2	DCE	–	50
8	1.2	DCE	–	71
9	1.2	DCE	*fac*-Ir(ppy)_3_	36

a Reactions were performed using 0.1 mmol of **1a**, [1.1.1]­propellane **3** as a solution in Et_2_O, and 0.4 mL of a solvent. The first and second steps ran for 3 and 2 h, respectively. Abbreviations: DCE, 1,2-dichloroethane; **DHA**, 12-methyl-12-phenyl-7,12-dihydrobenzo­[*a*]­acridine; r.t., room temperature.

b Determined by ^1^H NMR analysis of the crude mixture using trichloroethylene as an internal standard.

c Reaction in the absence of light.

d Reaction performed with a λ = 390 nm LED lamp.

Then, we performed further investigation to clarify the role of the photocatalyst and the overall reaction mechanism. Stern–Volmer quenching studies revealed that **DHA** emission is effectively quenched by **2a** with a *k*_q_ of 2.9 × 10^9^ M^–1^ s^–1^, which indicates that quenching at a diffusion-controlled rate can occur (Figure 2A). A reaction between glucosyl iodide **2a** and propellane **3** was completely inhibited in the presence of the 2,2,6,6-tetramethyl-1-piperidinyloxy radical (TEMPO), suggesting the involvement of radical species in the transformation (see the Supporting Information). We then investigated the behavior of propellane **3** in the presence of Me_3_SiI and found that the two compounds undergo electrophilic ring opening to afford a methylenecyclobutane derivative (65% (see the Supporting Information)). This finding explains the need for *in situ* preactivation of the substrate and the strong dependence of the reaction efficiency on Me_3_SiI stoichiometry. Interestingly, monitoring of the reaction over time by ^1^H NMR spectroscopy revealed a relatively complex reaction profile (Figure 2B). Treatment of **1a** with Me_3_SiI at 0 °C for 3 h selectively gives the activated glucosyl iodide as the β-anomer (β-**2a**). This species is known to be in equilibrium with the more stable α-**2a**.^22^ Indeed, during the course of the photochemical reaction, we observed the consumption of β-**2a** with concomitant formation of α-**2a** and ATRA product **4a**. After reaching a maximum, the level of α-**2a** also progressively decreased with an increase in the level of formation of **4a** (for details, see the Supporting Information).

**Figure 2:**
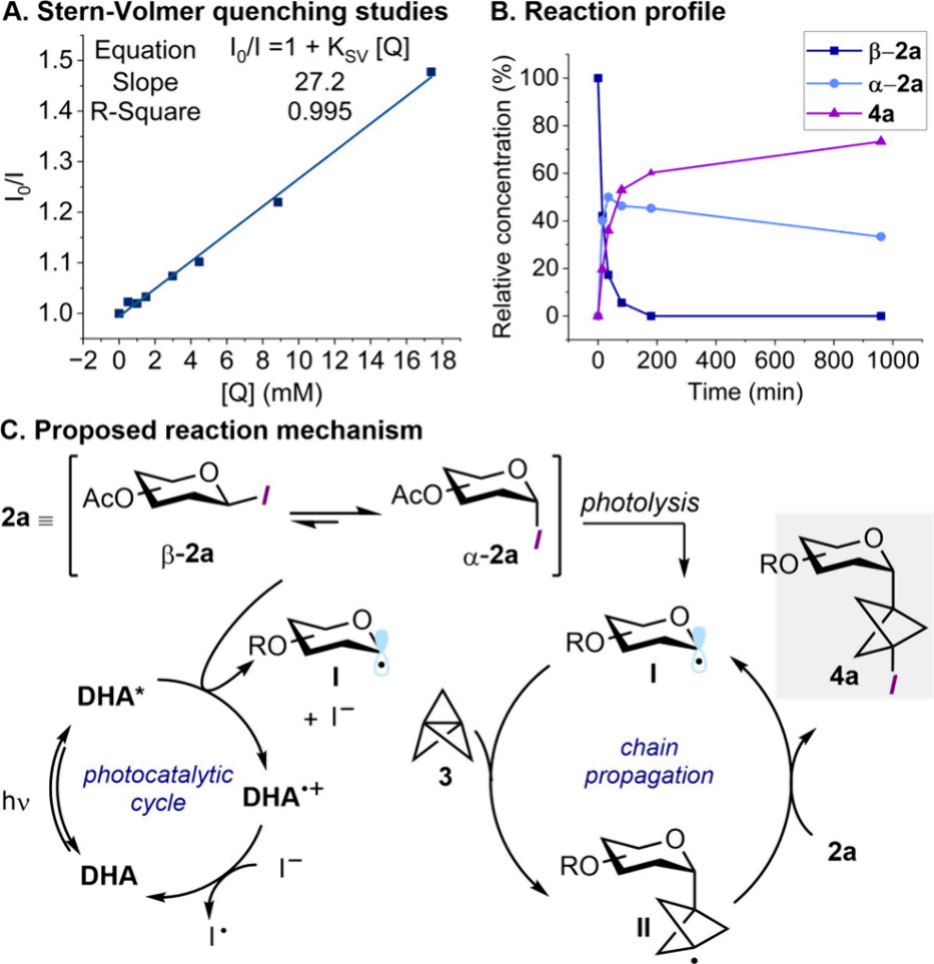
(A) Stern–Volmer quenching studies of **DHA** emission upon addition of increasing quantities of **2a**. (B) Distribution of β-**2a**, α-**2a**, and **4a** during the reaction course as determined by ^1^H NMR analysis of the crude mixture. (C) Proposed reaction mechanism.

Based on these experimental data, we propose the following reaction mechanism (Figure 2C). Upon light absorption, **DHA** reaches the excited state (DHA*). This highly reducing species undergoes thermodynamically favored SET with **2a** that, after fragmentation, gives key glycosyl radical **I**, along with I^–^. A SET oxidation of I^–^ (*E*_ox_(**I** ^**•**^ **/I** ^**–**^) = +0.26 V vs SCE in CH_3_CN)^23^ from ground state **DHA** ^**•+**^ (*E*_ox_(**DHA** ^**•+**^/**DHA**) = +0.76 V vs SCE in CH_3_CN) closes the photocatalytic cycle. Besides, direct photolysis of **2a** also contributes to the generation of glycosyl radical **I**. This intermediate is readily trapped by propellane **3** in a ring-opening step to give BCP radical **II**. Finally, **II** reacts via halogen atom transfer (XAT) with another molecule of **2a** to give ATRA product **4a** and a further glycosyl radical **I**, thus sustaining the radical chain reaction.^24^ Such reactivity occurs in a highly dynamic system, with glycosyl iodide β-**2a** being involved in equilibration toward more stable anomer α-**2a**.^25^ We hypothesize that, under these conditions, chain propagation might be relatively inefficient. In this context, the increased efficiency observed in the presence of **DHA** suggests its ability to reinitiate new radical chains, thereby compensating for premature chain termination (for details, see Table S3).

Adopting the optimized conditions described in entry 5 of Table 1, we investigated the generality of the photocatalytic one-pot protocol for a variety of bench-stable saccharides (0.2 mmol scale reaction (Table 2)). Isolation of the model substrate proceeded smoothly, giving **4a** in 89% yield. Notably, the reaction could be performed on a 10-fold scale simply using a batch reactor, with an only moderate decrease in performance (2 mmol, 51% yield). Then, we evaluated variously protected d-glucosyl derivatives. A per-pivaloylated derivative behaved well (**4b**), while a low yield was observed for per-benzoylated **4c** because of a moderate level of formation of the glycosyl iodide intermediate. On the contrary, we were pleased to see that the reaction was amenable to armed derivatives. Glucosides protected as benzyl and methyl ethers are known to be orders of magnitude more reactive than the corresponding per-acetylated derivatives in glycosylation reactions; nevertheless, they reacted smoothly giving **4d** and **4e** in good to moderate yields.^16h,26^ Even a more reactive per-silylated derivative reacted well, allowing easy access to unprotected **4f** upon acidic treatment of the reaction mixture with trifluoroacetic acid in methanol.^27^ Finally, acetonides could also be accommodated, enabling access to d-mannosyl derivatives in the furanosidic and pyranosidic forms (**4g** and **4h**, respectively).

**Table 2:**
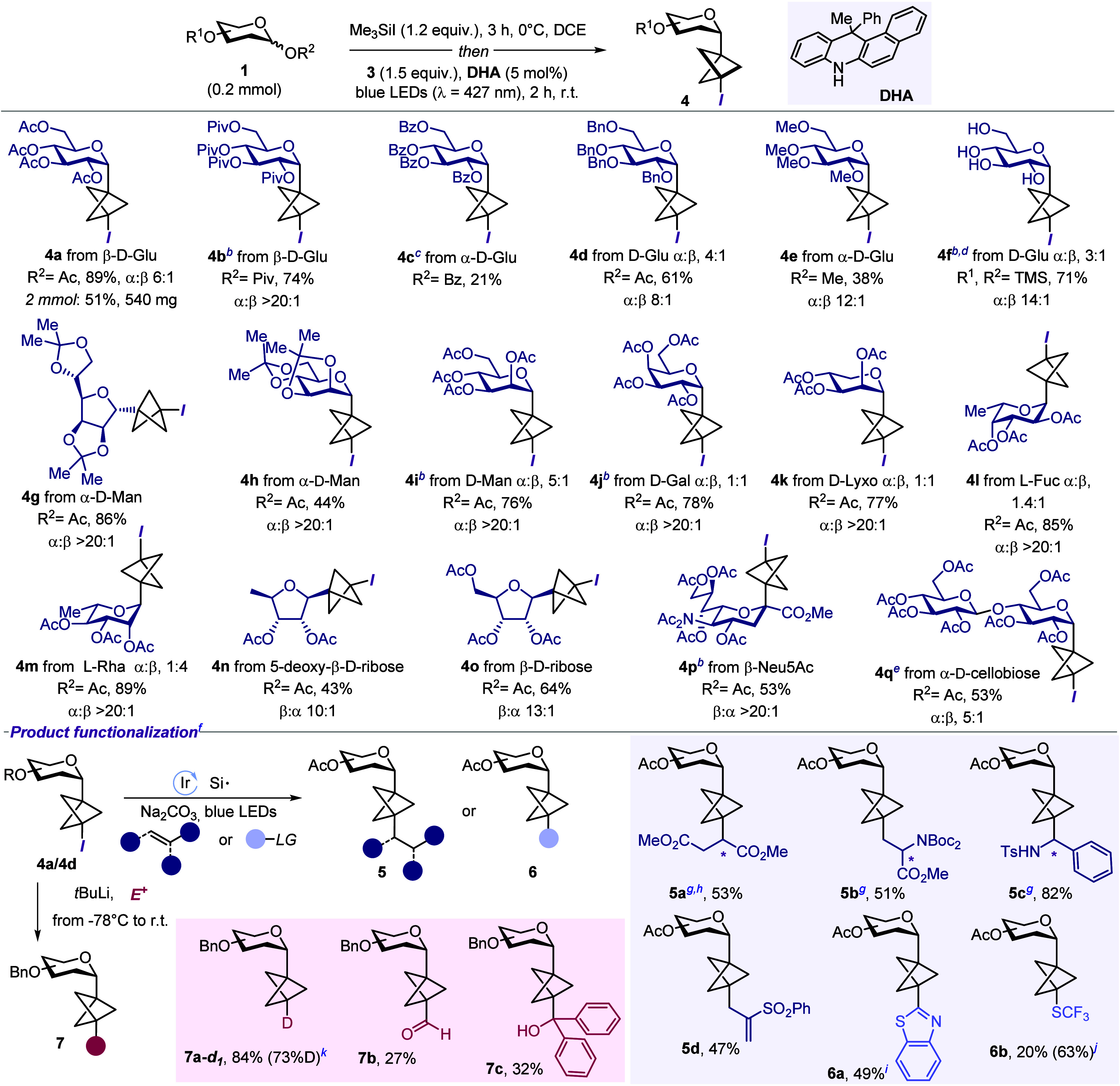
Survey of Glycosides for the Synthesis of Iodo BCP *C*-Glycosides and Postfunctionalization Studies^a^

a Reactions were performed on a 0.2 mmol scale of **1** using Me_3_SiI (1.2 equiv), [1.1.1]­propellane **3** as a solution in Et_2_O (1.5 equiv), **DHA** (0.5 mol %), and 0.8 mL of a solvent. Yields refer to isolated products **4**. α:β ratios were determined by ^1^H NMR analysis of the crude mixture using trichloroethylene as the internal standard. Abbreviations: DCE, 1,2-dichloroethane; **DHA**, 12-methyl-12-phenyl-7,12-dihydrobenzo­[*a*]­acridine; r.t., room temperature; TFA, trifluoroacetic acid.

b Iodination step for 6 h.

c Iodination step with ZnI_2_ (20 mol %). Yield determined by ^1^H NMR analysis.

d The reaction crude was treated with TFA (0.4 mmol) in MeOH prior to isolation.

e The preformed glycosyl iodide substrate was used.

f Photocatalyzed reactions: **4a** (0.1 mmol), Ir­[(dF­(CF_3_)­ppy)_2_(dtbbpy)]­PF_6_ (2.5 mol %), (Me_3_Si)_3_SiH (2 equiv), Na_2_CO_3_ (2 equiv), a radical acceptor (3 equiv), and blue LEDs (λ = 456 nm). Li–I exchange/addition reactions: **4d** (0.1 mmol), *t*BuLi (2.2 equiv), and an electrophile (3 equiv).

g **5a**–**c** obtained with 1:1 d.r.

h With 6 equiv of dimethyl fumarate and a 9:1 MeOH/H_2_O mixture as the solvent.

i With (Me_3_Si)_3_SiOH instead of (Me_3_Si)_3_SiH.

j The yield in parentheses refers to the compound prior to isolation.

k The reaction was run on a 0.05 mmol scale.

Next, we explored the saccharide scope. Per-acetylated hexoses and pentoses reacted well, giving d-Man (**4i**), d-Gal (**4j**), and d-Lyxo (**4k**) derivatives. Deoxysugars and furanoses were also competent substrates, including d-Fuc, d-Rha, 5-deoxy-d-ribose, and d-ribose compounds (**4l**–**o**, respectively). The protocol’s mild conditions also allowed for the synthesis of *N*-acetylneuraminic acid derivative **4p**, which is notable given the strong tendency of sialosyl iodides to undergo elimination forming α,β-unsaturated ester side products.^28^ As a limitation of the method, we found disaccharides to react with low yields (≤30% as judged by ^1^H NMR analysis), possibly due to interglycosidic bond cleavage during the Me_3_SiI-mediated activation.^16c^ However, for this specific case, we demonstrated that the desired BCP *C*-glycosides can be obtained using the preformed glycosyl iodides, as exemplified in the synthesis of a d-cellobiose derivative (**4q**).

Overall, the alkylated products were obtained with good to excellent stereoselectivities. Pyranosides preferentially gave α-anomers, which is consistent with a pronounced kinetic anomeric effect.^29^ Among these, per-acetylated glucoside **4a** showed the lowest selectivity (α:β 6:1), a result that is in agreement with those of our previous studies on the addition of glucosyl radicals to [1.1.1]­propellane.^14b^ On the other hand, the high α selectivity observed for mannofuranoside **4g** is most likely the result of steric effects from the two dioxolane rings, which shield the approach from the β face.^30^ Likewise, steric effects are mostly responsible for the good β selectivity shown by ribofuranosides **4n** and **4o**.^30b,31^

To demonstrate the synthetic versatility of the ATRA products, we targeted their derivatization taking advantage of the iodo functional group (Table 2, bottom). An Ir-photocatalyzed protocol enabled radical generation from per-acetylated **4a** and subsequent reaction with a series of SOMOphiles.^32^ Giese-type addition to dimethyl fumarate proceeded well, giving alkylated **5a**. Similarly, addition to a dehydroalanine derivative gave α-amino acid **5b** as a saturated analogue of a *C*-glycosyl phenylalanine. Radical addition to an aromatic aldimine proceeded in very good yield (**5c**), while radical addition/elimination with an allylsulfone derivative enabled the synthesis of allylated **5d**. Homolytic aromatic substitution with a benzothiazole sulfone gave heteroarylated **6a**. Reaction with *N*-(trifluoromethylthio)­phthalimide enabled functionalization of the BCP core with the SCF_3_ moiety, a relevant functional group in medicinal chemistry (**6b**). In addition, with per-benzylated **4d** in hand, we could explore its derivatization via lithium–iodine exchange followed by reaction with various electrophiles. Quenching of the organolithium intermediate with CD_3_OD gave deuterated **7a-** *d*_1_, while addition to ethyl formate and benzophenone enabled formylation (**7b**) and functionalization as a tertiary alcohol (**7c**).

In summary, we developed a straightforward and atom-economical approach for the direct synthesis of BCP *C*-glycosides from a wide range of simple, unactivated glycosides. By overcoming key limitations of existing *C*-glycosylation methods, this work will stimulate further development of catalytic tools for the functionalization of complex saccharides.^33^

## Supplementary Material



## Data Availability

The data underlying this study are available in the published article and its Supporting Information.

## Notes

Experimental procedures, full characterization data, and copies of NMR spectra (PDF)
