# Supplementary material for: Photocatalytic In Situ Activation of Bench-Stable Saccharides: A General Synthetic Strategy for C‑Glycosyl Bicyclo[1.1.1]pentanes
Source: Org Lett. 2026 Jun 8;28(24):7881–6. doi: 10.1021/acs.orglett.6c02105 (PMC13288914; doi:10.1021/acs.orglett.6c02105)

Supplementary Information for

Photocatalytic In Situ Activation of Bench-Stable Saccharides: A General Synthetic Strategy to *C*-Glycosyl Bicyclo[1.1.1]pentanes

Chun Qi, Luca Dell'Amico,* Giulio Goti,*

Department of Chemical Sciences, University of Padova, Via Marzolo 1, 35131, Padova, Italy

*Correspondence to: luca.dellamico@unipd.it and giulio.goti@unipd.it

Table of Contents

A. GENERAL INFORMATION **3**

B. SYNTHESIS OF STARTING MATERIALS **6**

B.1. Synthesis of 1,2,3,4,6-penta-*O*-acetyl-β-D-glucopyranose **1a** 6

B.2 Synthesis of 1,2,3,4,6-penta-*O*-pivaloyl-β-D-glucopyranose **1b** 6

B.3. Synthesis of methyl 2,3,4,6-tetra-*O*-methyl-α-D-glucopyranoside **1e** 7

B.4. Synthesis of 1,2,3,4,6-penta-*O*-trimethylsilyl-D-glucopyranose **1f** 7

B.5. Synthetic procedure for 1-*O*-acetyl-2,3:5,6-di-*O*-isopropylidene-α-D-mannofuranose **1g** 8

B.6. Synthetic procedure for 1-*O*-acetyl-2,3:4,6-di-*O*-isopropylidene-α-D-mannopyranose **1h** 9

B.7. General procedure for the synthesis of glucosyl benzoate 1c and glycosyl acetates **1d,i-m**........ 10

B.8. Synthetic procedure for methyl 2,4,7,8,9-penta-*O*-acetyl-5-(*N*-acetylacetamido)-3,5-dideoxy-D-*glycero*-β-D-*galacto*-non-2-ulopyranosonate **1p** 12

B.9. Synthetic procedure for 2,3,6,2',3',4',6'-hepta-*O*-acetyl-α-D-cellobiosyl iodide **2q** 14

B.10. Preparation of [1.1.1]propellane (**3**) solution in Et_2O 15

C. OPTIMIZATION OF THE REACTION CONDITIONS **16**

D. SYNTHESIS OF *C*-GLYCOSYL IODO BCPs **18**

D.1. General procedure for the synthesis of *C*-glycosyl iodo BCPs **4** from unactivated saccharides 18

D.2. Synthesis of 1-(2,3,6,2',3',4',6'-hepta-*O*-acetyl-α-D-cellobiopyranosyl)-3-iodobicyclo[1.1.1]pentane **4q** 26

D.3. Unsuccessful substrates........ 27

F. REACTION SCALE UP AND PRODUCT DERIVATIZATIONS **27**

F.1. General procedure for derivatization via photocatalysis 27

F.2. General procedure for derivatization via Li/I exchange and electrophile trapping 31

G. MECHANISTIC INSIGHTS **33**

G.1. UV-Vis absorption spectra 34

G.2. Stern-Volmer quenching studies 35

G.3. Cyclic voltammetry measurements 37

G.4. TEMPO inhibition experiments 38

G.5. Investigation on the compatibility of iodotrimethylsilane with [1.1.1]propellane **3** 38

G.6. Reaction profile 40

I. REFERENCES 43

J. NMR SPECTRA 46

A. GENERAL INFORMATION

Analytical Methods. NMR spectra were recorded on a Bruker Avance 300 spectrometer equipped with a BBO-z grad probehead, Bruker AVANCE Neo 400 Nanobay equipped with a BBFO-ATM-z grad probehead, on a Bruker 400 AVANCE III HD spectrometer equipped with a BBI-z grad probehead. The chemical shifts (δ) for ^{1}H and ^{13}C are given in ppm relative to residual signals of the solvents ($CHCl_3$-d_1: 7.26 ppm for ^{1}H NMR and 77.16 ppm for ^{13}C NMR; CH_3OH-d_4: 3.31 ppm for ^{1}H NMR and 49.00 ppm for ^{13}C NMR; D_2O: 4.79 ppm for ^{1}H NMR). Coupling constants are given in Hz. The following abbreviations are used to indicate the multiplicity: s, singlet; d, doublet; t, triplet; q, quartet; quint, quintet; m, multiplet; b, broad signal. NMR yields were calculated by using trichloroethylene as internal standard. Structural assignments were made with additional information from COSY, NOESY, HSQC, and HMBC experiments.

High-Resolution Mass Spectra (HRMS) were obtained on a Xevo G2-XS QToF in the Department of Pharmaceutical Sciences (University of Padua), with electron spray ionization (ESI).

Optical rotation measurements were done on a Jasco Polarimeter P-1010. All the spectra were recorded at 25°C using a 1 dm path length Jasco polarimetry cell.

GC-MS analyses were performed by an Agilent Technology (7890A) equipped with an HP-5 column and MS spectrometer Agilent Technology 5975C.

Preparative HPLC purifications were performed on a Shimadzu LC-8A by using a C18 reversed-phase (RP) preparative column (Kinetex C18 150 mm × 21.20 mm, particle size 5 μm) at 30 °C using water/CH_3CN as mobile phase at a flow rate of 17 mL/min.

Absorption spectroscopy studies were performed at room temperature on a Varian Cary 50 UV-vis using a 1 mm path length Hellma Analytics quartz cuvette.

Steady-state emission spectroscopy studies were performed at room temperature on a Varian Cary Eclipse Fluorescence spectrophotometer using a 10 mm path length Hellma Analytics quartz cuvette.

Fluorescence lifetime measurements were performed on an FLS1000 Instrument (Edinburgh Instruments Ltd., U.K.) equipped with a pulsed ps laser at 402.6 nm (EPL 405), 10 MHz repetition rate, and an air-cooled single-photon counting photomultiplier (Hamamatsu R13456P) as detector. Fluorescence decays were recorded using the Time correlated single photon counting (TCSPC) method and analyzed with the Fluoracle Software using the IRF convolution fitting procedure.

The electrochemical characterizations were carried out using a BASi EC Epsilon potentiostat-galvanostat in a glass cell. A typical three-electrode cell was employed, which was composed of a glassy carbon (GC) working electrode (circle, diameter = 3 mm, surface area = 7.1 mm^2), a platinum counter electrode (circle, diameter = 1.5 mm, surface area = 1.8 mm^2), and Ag/AgCl (3M NaCl) as reference electrode. Before starting the measurements, the GC electrode and the platinum counter electrode were polished using a diamond polishing pad (BASI, PK-4 Polishing Kit, diamond polishing pad white, nylon). The GC electrode was polished using a

diamond paste (Struers, DP-Paste M - 1 μm), while no diamond paste was used for polishing the platinum counter electrode. Both electrodes were sonicated using a water:acetone, 1:1 solution for 5 minutes, then rinsed with water, and then with acetone. The GC electrode was polished following this procedure after each series of CV experiments. During the experiments, the glass electrochemical cell was kept closed with a stopper annexed to the potentiostat. Oxygen was removed by purging the solvent with N_2, introduced from a line into the cell by means of a glass pipe. The potential of ferrocenium/ferrocene E (Fc^+/Fc) couple was used as internal reference system to calibrate the potentiostat. All the results were subsequently converted in V vs SCE, in agreement with the value reported in literature [$E_{1/2}(Fc^+/Fc)$ = +0.38 V vs SCE].[1] The measurements were performed in a Bu_4NPF_6 (0.1 M) solution in CH_3CN at room temperature using the following parameters: initial potential = –0.08 V; switching potential = –2.83 V; final potential = –0.08 V; scan rate = 0.1 V/s.

General Procedures. All air and water-sensitive reactions were carried out in oven-dried glassware under argon atmosphere using standard Schlenk technique. The solvents were degassed when needed by bubbling argon for ten minutes. Synthesis grade solvents and anhydrous solvents were used as purchased. Chromatographic purification of products was accomplished using flash chromatography (FC) on silica gel (SiO_2, 0.04-0.063 mm) purchased from Merck Millipore - Supelco or using a Biotage Selekt automated flash chromatography system with cartridges packed with silica (Biotage Sfär silica - high-capacity duo, 20 μm). Thin layer chromatography (TLC) analysis was performed using Merck pre-coated TLC plates (silica gel 60 GF254, 0.25 mm), which were visualized by UV absorbance (254 nm) and/or using phosphomolybdic acid (PMA), basic aqueous potassium permanganate ($KMnO_4$), or acidic aqueous ceric ammonium molybdate stain solutions and heat as developing agents. Organic solutions were concentrated under reduced pressure on rotary evaporators (in vacuo at 40 ºC). Oil baths were used for heating the reactions. Unless otherwise stated, yields refer to isolated materials of ≥95% purity, as determined by ^{1}H NMR analysis. Slow addition of reagents at a given rate or continuous flow reactions were run using a Chemyx Inc Fusion 200-X Syringe Pump.

Materials. Commercially available reagents and solvents were purchased at the highest commercial quality from Sigma Aldrich, Fluorochem, TCI, BLDpharm and used as received, unless otherwise stated. 1,2,3-Tri-*O*-acetyl-5-deoxy-β-D-ribofuranose **1n**, tetra-*O*-acetyl-β-D-ribofuranose **1o**, and iodotrimethylsilane (stabilised with 0.1% Copper) were commercially available. **DHA**,[2] *N*,*N*-*tert*-butoxycarbonyldehydroalanine,[34] (*E*)-*N*-benzylidene-4-methylbenzenesulfonamide,[5] (prop-1-ene-2,3-diyldisulfonyl)dibenzene,[6] 2-(phenylsulfonyl)benzothiazole,[7] and 2-((trifluoromethyl)thio)isoindoline-1,3-dione[8] were prepared according to reported procedures.

Note: Iodotrimethylsilane (Me_3SiI) is a colorless liquid that gradually turns from pale yellow to dark brown upon prolonged storage. The reagent is moisture-sensitive and undergoes hydrolysis to give trimethylsilanol (Me_3SiOH) and HI. Notably, HI is also a competent reagent in the formation of glycosyl iodides.[9] In this study, reproducible results were obtained using either freshly opened Me_3SiI or previously opened batches stored for up to one year, containing Me_3SiI/Me_3SiOH mixtures of up to 1.3:1 ratio as judged by ^{1}H NMR analysis [Me_3SiI (400 MHz, $CDCl_3$, δ 0.80 ppm); Me_3SiOH (400 MHz, $CDCl_3$, δ 0.06 ppm)].

Light Sources. The light sources used in this work were purchased from Kessil (https://kessil.com/products/science_main.php):

- 390 nm: Kessil lamp PR160L-390 (max 52W).
- 427 nm: Kessil lamp PR160L-427 (max 45W).
- 456 nm: Kessil lamp PR160L-456 (max 50aW).

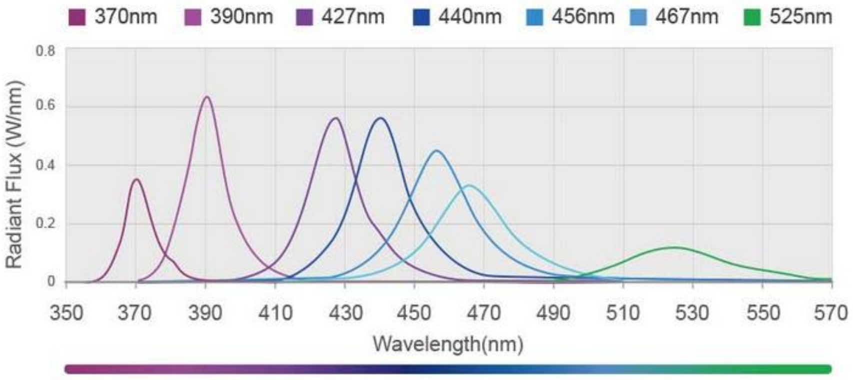

Figure S1. Emission spectra of the Kessil lamp PR160L reproduced from Kessil webpage (https://kessil.com/products/science_main.php).

Photochemical Set Ups.

The figures below show the general photochemical set up employed in the present study.

For photochemical reactions performed in 4 mL vial (Figure S2), the vessel containing the reaction mixture was placed at 2 cm from the light source and the mixture was stirred vigorously by a magnetic stirrer (IKA topolino). To maintain a stable reaction temperature, one fan was placed next to the vials.

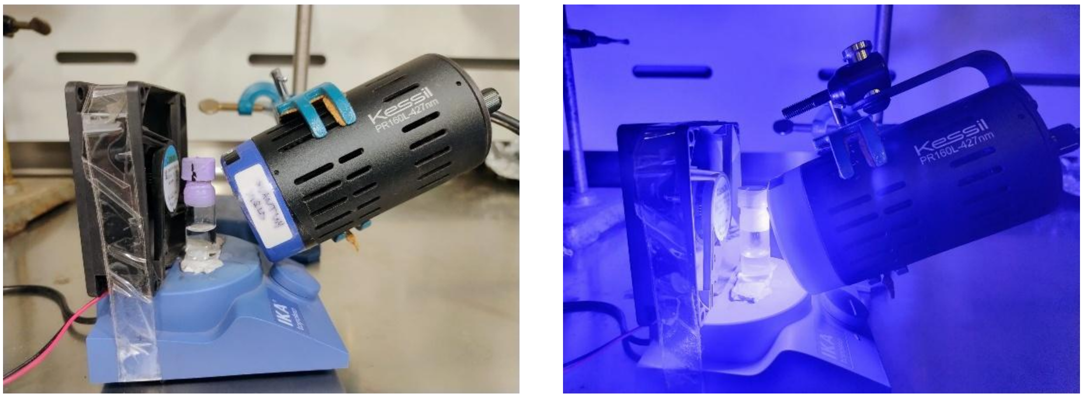

Figure S2. Photochemical set up for reactions performed in 4 mL vials.

An analogous set up was used for photochemical reactions performed using a 25 mL round bottom flask (Figure S3). In this case, two light sources were employed.

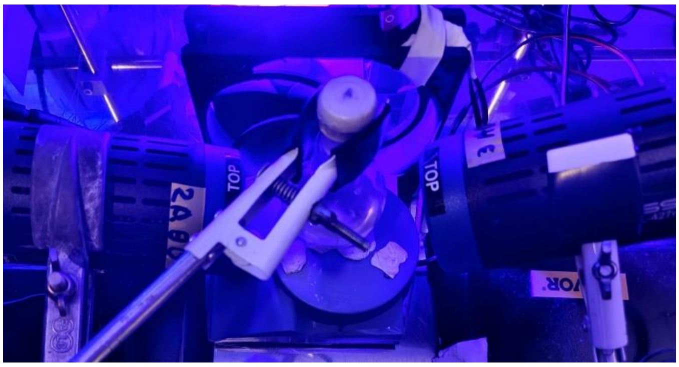

Figure S3. Photochemical set up for 2 mmol scale reaction using a 25 mL round bottom flask.

B. SYNTHESIS OF STARTING MATERIALS

B.1. Synthesis of 1,2,3,4,6-penta-*O*-acetyl-β-D-glucopyranose 1a

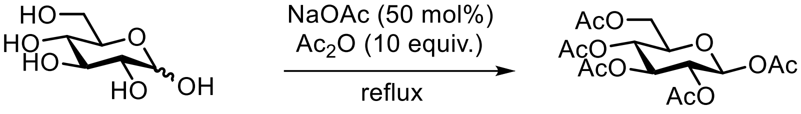

D-glucose

1a
54%

Compound **1a** was synthesized following an already reported procedure.[10] To a round bottom flask NaOAc (1.14 g, 13.9 mmol, 50 mol%) and Ac_2O (26 mL, 278 mmol, 10 equiv.) were added and the mixture heated at 120°C for 10 min. Then, D-glucose (5.00 g, 27.8 mmol) was slowly added over a period of 15 min and the mixture was stirred under vigorous reflux for 5 min. The mixture was cooled to room temperature, and the reaction was quenched by addition of ice and sonicated to allow the precipitation of a white powder. The precipitate was filtered and washed with H_2O to remove AcOH. The crude product was purified by recrystallization from hot EtOH to afford **1a** as a white solid (5.84 g, 54% yield). The spectroscopic data are consistent with those previously reported.[10]

^{1}H NMR (400 MHz, $CDCl_3$) δ 5.72 (d, *J* = 8.3 Hz, 1H), 5.25 (t, *J* = 9.4 Hz, 1H), 5.18 – 5.08 (m, 2H), 4.29 (dd, *J* = 12.5, 4.5 Hz, 1H), 4.11 (dd, *J* = 12.6, 2.2 Hz, 1H), 3.84 (ddd, *J* = 10.0, 4.6, 2.2 Hz, 1H), 2.12 (s, 3H), 2.09 (s, 3H), 2.03 (s, 3H), 2.03 (s, 3H), 2.01 (s, 3H).

B.2 Synthesis of 1,2,3,4,6-penta-*O*-pivaloyl-β-D-glucopyranose 1b

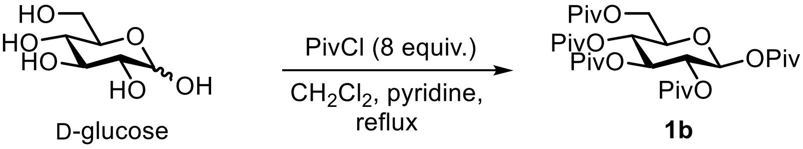

D-glucose

1b
54%

Compound **1b** was synthesized by modification of an already reported procedure.[11] To a round bottom flask D-glucose (1.0 g, 5.5 mmol) was suspended in CH_2Cl_2 (8 mL) and pyridine (4 mL) under nitrogen atmosphere. Then, pivaloyl chloride (3.4 mL, 27.8 mmol, 5 equiv.) was added and the mixture stirred under reflux overnight. Then, pivaloyl chloride (1.7 mL, 13.9 mmol, 2.5 equiv.) was further added and the reaction stirred

overnight to reach completion. The mixture was cooled down, dissolved in CH_2Cl_2 (20 mL), and the organic phase washed with a 30% w/v citric acid aqueous solution (2×10 mL). Then the aqueous phase was extracted with fresh CH_2Cl_2 (2×10 mL) and the reunited organic phases were washed with H_2O (20 mL), brine (20 mL), and dried over anhydrous Na_2SO_4. The mixture was filtered and the solvent evaporated under reduced pressure to give a crude mixture that was purified by flash chromatography (gradient Hex:EtOAc from 100:0 to 80:20) to give **1b** as a white solid (1.77 g, 54% yield). The spectroscopic data are consistent with those previously reported.[12]

^{1}H NMR (400 MHz, $CDCl_3$) δ 5.70 (d, *J* = 8.3 Hz, 1H), 5.37 (t, *J* = 9.4 Hz, 1H), 5.22 (t, *J* = 9.5 Hz, 1H), 5.16 (t, *J* = 9.7 Hz, 1H), 4.19 – 4.07 (m, 2H), 3.86 (ddd, *J* = 9.7, 5.2, 2.3 Hz, 1H), 1.21 (s, 9H), 1.18 (s, 9H), 1.15 (s, 9H), 1.12 (s, 18H).

B.3. Synthesis of methyl 2,3,4,6-tetra-*O*-methyl-α-D-glucopyranoside 1e

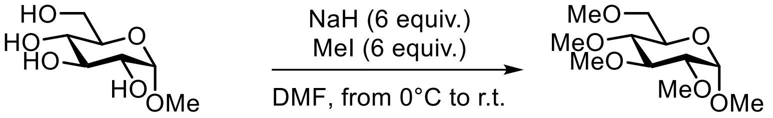

S1 **1e** 83%

Compound **1e** was synthesized by modification of an already reported procedure.[13] To a round bottom flask equipped with a magnetic stir bar, methyl α-D-glucopyranoside **S1** (1 g, 5.2 mmol, 1 equiv.) was added. The flask was evacuated and backfilled with nitrogen three times, then anhydrous DMF (50 mL) was added under nitrogen atmosphere. The mixture was cooled down to 0°C and NaH (60% in paraffin oil, 1.24 g, 31.2 mmol, 6 equiv.) was added. The mixture was stirred for 1 hour at room temperature and then MeI (1.9 mL, 31.2 mmol, 6 equiv.) was added at 0°C. The mixture was allowed to reach room temperature and stirred overnight. The reaction was then quenched with MeOH (25 mL) and the solvent was removed under reduced pressure. The crude was taken with CH_2Cl_2, H_2O (30 mL) was added and extracted with CH_2Cl_2 (3×30 mL). The reunited organic phases were washed with brine (1×30 mL), dried over anhydrous Na_2SO_4, filtered and the solvent was removed under reduced pressure. The crude was purified with flash column chromatography (petroleum ether:Et_2O, 40:60) to obtain **1e** as a pale-yellow oil (1.08 g, 83% yield). The spectroscopic data are consistent with those previously reported.[14]

^{1}H NMR (400 MHz, $CDCl_3$) δ 4.82 (d, *J* = 3.6 Hz, 1H), 3.62 (s, 3H), 3.61 – 3.56 (m, 3H), 3.54 (s, 3H), 3.53 – 3.46 (m, 4H), 3.41 (s, 3H), 3.41 (s, 3H), 3.24 – 3.15 (m, 2H).

B.4. Synthesis of 1,2,3,4,6-penta-*O*-trimethylsilyl-D-glucopyranose 1f

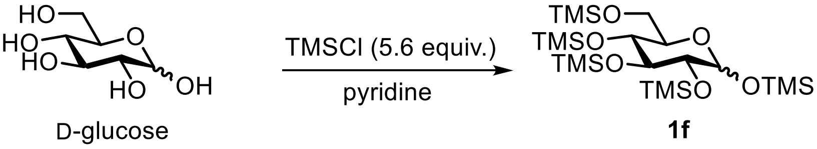

D-glucose **1f** 93%

Compound **1f** was synthesized by modification of an already reported procedure.[15] To a round bottom flask equipped with a magnetic stir bar, D-glucose (1 g, 5.5 mmol, 1 equiv.) was added. The flask was evacuated and backfilled with nitrogen three times, then anhydrous pyridine (5 mL) and TMSCl (4 mL, 31 mmol, 5.6 equiv.) were added under nitrogen atmosphere. The mixture was stirred at room temperature for 4 h, then Hex (40 mL) was added followed by crashed ice (10 mL). The crude mixture was transferred into a separatory funnel, the organic phase was washed with ice water (4×10 mL), and dried over anhydrous Na_2SO_4. The mixture was filtered and the solvent was removed under reduced pressure to give **1f** as a mixture of anomers in a 3.1:1 α:β ratio as a colorless liquid (2.08 g, 93% yield) that was used without further purification. The spectroscopic data are consistent with those previously reported.[16]

^{1}H NMR (400 MHz, $CDCl_3$, anomer mixture) δ 5.00 (d, J = 3.0 Hz, 3.1H, H-1α), 4.45 (d, J = 7.3 Hz, 1H, H-1β), 3.82 – 3.56 (m, 14H), 3.44 – 3.36 (m, 5H), 3.33 (dd, J = 9.1, 3.0 Hz, 3.1H), 3.26 – 3.18 (m, 2H), 0.17 (s, 36H), 0.14 (s, 82H), 0.13 (s, 28H), 0.10 (s, 36H).

B.5. Synthetic procedure for 1-*O*-acetyl-2,3:5,6-di-*O*-isopropylidene-α-D-mannofuranose 1g

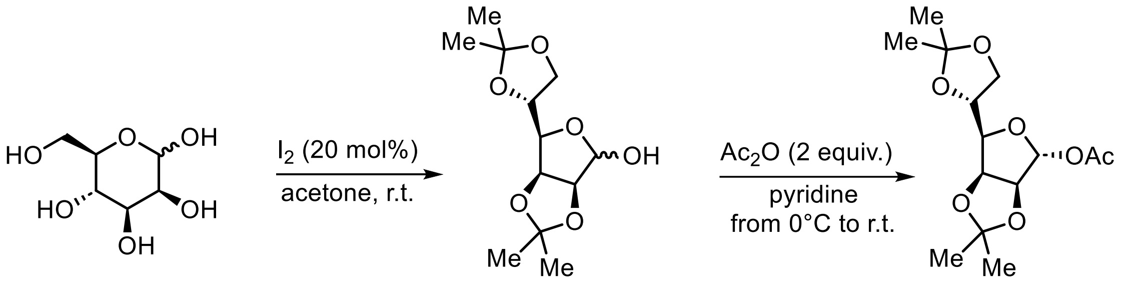

D-Man S2 90% 1g 99%

Step 1: Compound **S2** was synthesized through slight modification of an already reported procedure.[17] To a suspension of D-mannose (2.2 g, 12.2 mmol) in acetone (96 mL), iodine (650 mg, 2.55 mmol, 0.2 equiv.) was added and the mixture was stirred for 2 h at room temperature. The reaction mixture was quenched at 0 °C with saturated aqueous solutions of $Na_2S_2O_3$ (30 mL) and $NaHCO_3$ (150 mL). The mixture was concentrated to remove the majority of acetone and then extracted with chloroform (150 mL). Then the organic phase was washed with a saturated aqueous solution of $NaHCO_3$ (3×20 mL), the organic layer was dried over anhydrous Na_2SO_4, filtered and concentrated under reduced pressure to give **S2** as a pale yellow solid (2.84 g, 90%), which was used without further purification in the next step.

Step 2: Compound **1g** was synthesized following an already reported procedure.[18] To a round bottom flask, **S1** (2.84 g, 10.9 mmol) was added and dissolved in anhydrous pyridine (57 mL). Then Ac_2O (2.1 mL, 21.8 mmol, 2 equiv.) was added at 0°C and the mixture stirred at room temperature for 3h. The rection solution was concentrated under reduced pressure, and the residue was co-evaporated with toluene (2×25 mL) under reduced pressure. Purification by column chromatography (petroleum ether: EtOAc, 80:20) gave compound **1g** as a colorless syrup (3.27 g, 99%). The spectroscopic data are consistent with those previously reported.[18]

^{1}H NMR (400 MHz, CDCl$_3$) δ 6.12 (s, 1H), 4.85 (dd, *J* = 6.0, 3.5 Hz, 1H), 4.69 (d, *J* = 5.8 Hz, 1H), 4.51 – 4.33 (m, 1H), 4.17 – 4.07 (m, 1H), 4.07 – 3.99 (m, 2H), 2.07 (s, 3H), 1.48 (s, 3H), 1.46 (s, 3H), 1.38 (s, 3H), 1.34 (s, 3H).

B.6. Synthetic procedure for 1-*O*-acetyl-2,3:4,6-di-*O*-isopropylidene-α-D-mannopyranose 1h

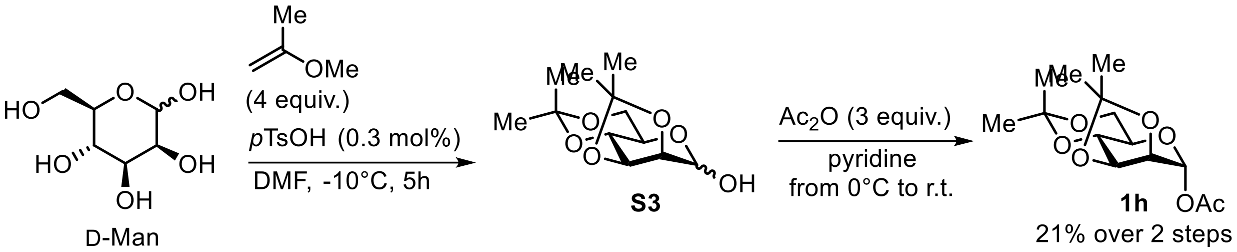

Compound **1h** was synthesized following an already reported procedure.[19]

Step 1: To a round bottom flask was added D-mannose (2.00 g, 11.1 mmol) and $CaSO_4$ (Drierite; 370 mg, 2.7 mmol) as a fine powder. The flask was evacuated and backfilled with nitrogen three times, then anhydrous DMF (7.4 mL) was added under nitrogen atmosphere. Then the mixture was cooled down to −10°C, 2-methoxyprop-1-ene (2.1 mL, 22.2 mmol) and *p*-TsOH (7.4 mg, 39 μmol, 0.3 mol%) were added, and the mixture stirred at −10°C for 3 h. Then, another portion of 2-methoxyprop-1-ene (2.1 mL, 22.2 mmol) was added dropwise during 2 h using a syringe-pump while keeping the reaction mixture at -10°C. The mixture was then filtered into a separatory funnel containing ice water (50 mL) and extracted with CH_2Cl_2 (4×20 mL). The reunited organic phase was washed with H_2O (4×20 mL), dried over anhydrous Na_2SO_4, filtered, and the solvent removed under reduced pressure. Crude **S3** was used in the next acetylation step without further purification.

Step2: Crude **S3** was dissolved in anhydrous pyridine (7.4 mL) and Ac_2O (3.1 mL, 33.3 mmol, 3 equiv.) was added at 0°C. The mixture was allowed to reach room temperature and stirred overnight. Then, the mixture was poured into a separatory funnel containing ice water (50 mL) and extracted with CH_2Cl_2 (2×50 mL). The reunited organic phase was washed with saturated $NaHCO_3$ aqueous solution (3×20 mL), dried over anhydrous Na_2SO_4, filtered, and the solvent removed under reduced pressure. The crude was purified by column chromatography (gradient from Hex:EtOAc, 98:10 to 90:10) to give compound **1h** as a colorless syrup (700 mg, 21% over two steps). The spectroscopic data are consistent with those previously reported.[20]

Note: precise control of the temperature is key to obtain the desired mannoside in its pyranosidic form.

Caution: The related 1-*O*-acetyl-2,3:5,6-di-*O*-isopropylidene-α-D-manno*furanose* has been often misassigned as **1h**. While chemical shifts values for **1h** are reported in the literature,[20] copies of the ^{1}H and ^{13}C NMR spectra are not available. To prevent further error propagation, we decided to report them in Section J.

^{1}H NMR (300 MHz, CDCl$_3$) δ 6.31 (s, 1H), 4.25 – 4.13 (m, 2H), 3.91 (dd, *J* = 10.6, 5.4 Hz, 1H), 3.84 – 3.69 (m, 2H), 3.62 (td, *J* = 10.0, 5.4 Hz, 1H), 2.12 (s, 3H), 1.56 (s, 3H), 1.52 (s, 3H), 1.43 (s, 3H), 1.36 (s, 3H).

^{13}C NMR (101 MHz, CDCl$_3$) δ 168.9, 110.1, 100.0, 91.6, 75.4, 74.8, 72.4, 63.9, 62.0, 29.1, 28.2, 26.3, 21.1, 18.9.

B.7. General procedure for the synthesis of glucosyl benzoate 1c and glycosyl acetates 1d,i-m

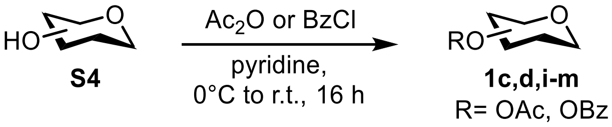

The following protocol was adapted from an already reported procedure.[21]

To a round bottom flask, equipped with a magnetic stir bar, the unprotected carbohydrate **S4** (1 equiv.) was added. The flask was evacuated and backfilled with nitrogen three times, and then anhydrous pyridine was added under nitrogen atmosphere. The mixture was cooled down to 0°C and acetic anhydride or benzoyl chloride (~ 1.35 equiv./OH group) was added dropwise. The reaction was allowed to reach r.t. and stirred for 16 h. Then the mixture was quenched by adding water (20 mL) and stirred for 15 minutes. The mixture was poured in an extraction funnel and extracted with CH_2Cl_2 (3 x 10 mL), then the organic fraction was washed with an HCl 2 M aqueous solution (3 x 10 mL), saturated $CuSO_4$ aqueous solution (2 x 10 mL), H_2O (1 x 20 mL), saturated Na_2CO_3 aqueous solution (3 x 10 mL), and brine (1 x 10 mL). The organic layer was then dried with anhydrous $MgSO_4$, filtered, and concentrated *in vacuo* to give **1** that was used without further purification or purified by flash column chromatography.

1,2,3,4,6-Penta-*O*-benzoyl-α-D-glucopyranose 1c

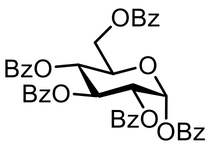

1c

Compound **1c** was synthesized according to general procedure B.7. using D-glucose (2.00 g, 11.1 mmol, 1 equiv.), benzoyl chloride (8.4 mL, 72.3 mmol, 6.5 equiv.) in anhydrous pyridine (12 mL). Product **1c** was obtained as a white solid (4.98 g, 64% yield). The spectroscopic data are consistent with those previously reported.[21]

^{1}H NMR (400 MHz, $CDCl_3$) δ 8.17 (d, *J* = 7.3 Hz, 2H), 8.03 (d, *J* = 7.5 Hz, 2H), 7.95 (d, *J* = 7.5 Hz, 2H), 7.88 (d, *J* = 7.4 Hz, 4H), 7.71 – 7.64 (m, 1H), 7.58 – 7.27 (m, 14H), 6.85 (d, *J* = 3.7 Hz, 1H), 6.32 (t, *J* = 10.0 Hz, 1H), 5.86 (t, *J* = 9.9 Hz, 1H), 5.68 (dd, *J* = 10.3, 3.8 Hz, 1H), 4.66 – 4.58 (m, 2H), 4.52 – 4.44 (m, 1H).

1-*O*-acetyl-2,3,4,6-tetra-*O*-benzyl-D-glucopyranose 1d

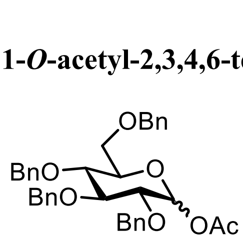

1d

Compound **1d** was synthesized according to general procedure B.7. using 2,3,4,6-tetra-*O*-benzyl-D-glucopyranose (2.00 g, 3.7 mmol, 1 equiv.), acetic anhydride (0.43 mL, 4.5 mmol, 1.2 equiv.) in anhydrous pyridine (12 mL). Product **1d** was obtained in a 4.3:1, α:β ratio as a colorless syrup (1.60 g, 74% yield). The spectroscopic data are consistent with those previously reported.[22]

^{1}H NMR (400 MHz, $CDCl_3$, major anomer) δ 7.40 – 7.22 (m, 18H), 7.18 – 7.08 (m, 2H), 6.35 (d, *J* = 3.5 Hz, 1H), 4.96 (d, *J* = 10.8 Hz, 1H), 4.86 – 4.79 (m, 2H), 4.70 – 4.57 (m, 3H), 4.42 – 4.45 (m, 2H), 3.94 (t, *J* = 9.4 Hz, 1H), 3.90 – 3.84 (m, 1H), 3.78 – 3.61 (m, 5H), 2.13 (s, 3H).

1,2,3,4,6-Penta-*O*-acetyl-D-mannopyranose 1i

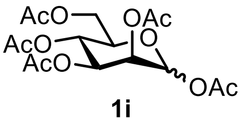

1i

Compound **1i** was synthesized according to general procedure B.7. using D-mannose (1.00 g, 5.55 mmol, 1 equiv.), acetic anhydride (4.2 mL, 44 mmol, 6.75 equiv.) in anhydrous pyridine (10 mL). Product **1i** was obtained as a mixture of anomers in a 5:1 α:β ratio as a colorless syrup (1.1 g, 50% yield). The spectroscopic data are consistent with those previously reported.[21]

^{1}H NMR (400 MHz, $CDCl_3$, anomer mixture) δ 6.09 (d, *J* = 2.1 Hz, 1H), 5.39 – 5.32 (m, 2H), 5.26 (d, *J* = 2.3 Hz, 1H), 4.28 (dd, *J* = 12.3, 4.8 Hz, 1H), 4.17 – 4.01 (m, 3H), 2.18 (s, 3H), 2.17 (s, 3H), 2.09 (s, 3H), 2.05 (s, 3H), 2.01 (s, 3H).

1,2,3,4,6-Penta-*O*-acetyl-D-galactopyranose 1j

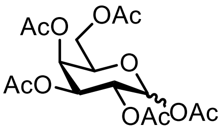

1j

Compound **1j** was synthesized according to general procedure B.7. using D-galactose (1.00 g, 5.55 mmol, 1 equiv.), acetic anhydride (4.2 mL, 44 mmol, 6.75 equiv.) in anhydrous pyridine (10 mL). Product **1j** was obtained as a mixture of anomers in a 1:1 α:β ratio as a colorless syrup (1.3 g, 60% yield). The spectroscopic data are consistent with those previously reported.[23]

^{1}H NMR (400 MHz, $CDCl_3$, anomer mixture) δ 6.38 (d, *J* = 1.9 Hz, 1H), 5.70 (d, *J* = 8.3 Hz, 1H), 5.50 (d, *J* = 1.4 Hz, 1H), 5.43 (dd, *J* = 3.4, 1.3 Hz, 1H), 5.38 – 5.29 (m, 3H), 5.08 (dd, *J* = 10.4, 3.4 Hz, 1H), 4.34 (dd, *J* = 7.4, 6.0 Hz, 1H), 4.21 – 4.01 (m, 5H), 2.16 (s, 3H), 2.16 (s, 3H), 2.16 (s, 3H), 2.12 (s, 3H), 2.04 (s, 9H), 2.02 (s, 3H), 2.00 (s, 3H), 1.99 (s, 3H).

1,2,3,4-Tetra-*O*-acetyl-D-lyxopyranose 1k

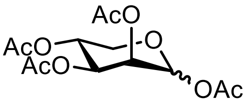

1k

Compound **1k** was synthesized according to general procedure B.7. using D-lyxose (1.00 g, 6.66 mmol, 1 equiv.), acetic anhydride (3.5 mL, 36.6 mmol, 5.5 equiv.) in anhydrous pyridine (12 mL). Product **1k** was obtained as a mixture of anomers in a 1:1.1 α:β ratio as a white solid (1.91 g, 90% yield). The spectroscopic data are consistent with those previously reported.[24]

^{1}H NMR (400 MHz, $CDCl_3$, anomer mixture) δ 6.01 (d, *J* = 3.4 Hz, 1H), 5.99 (d, *J* = 3.8 Hz, 1H), 5.40 – 5.34 (m, 2H), 5.26 – 5.15 (m, 3H), 5.04 (q, *J* = 6.3 Hz, 1H), 4.19 (dd, *J* = 12.6, 3.9 Hz, 1H), 4.00 (dd, *J* = 11.4, 5.0 Hz, 1H), 3.70 (dd, *J* = 11.7, 8.2 Hz, 1H), 3.61 (dd, *J* = 12.4, 5.3 Hz, 1H), 2.15 (s, 3H), 2.13 (s, 3H), 2.12 (s, 3H), 2.11 (s, 3H), 2.09 (s, 6H), 2.07 (s, 3H), 2.05 (s, 3H).

1,2,3,4-Tetra-*O*-acetyl-L-fucopyranose 1l

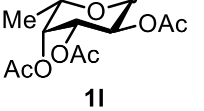

1l

Compound **1l** was synthesized according to general procedure B.7. using L-fucose (1.00 g, 6.09 mmol, 1 equiv.), acetic anhydride (3.17 mL, 35.5 mmol, 5.8 equiv.) in anhydrous pyridine (12 mL). The product **1l** was obtained as a mixture of anomers in a 1.4:1 α:β ratio

as a pale-yellow syrup (1.75 g, 86% yield). The spectroscopic data are consistent with those previously reported.[21]

^{1}H NMR (400 MHz, $CDCl_3$, anomer mixture) δ 6.34 (d, *J* = 2.6 Hz, 1H), 5.68 (d, *J* = 8.2 Hz, 1H), 5.39 – 5.29 (m, 4H), 5.27 (d, *J* = 3.2 Hz, 1H), 5.07 (dd, *J* = 10.4, 3.4 Hz, 1H), 4.27 (q, *J* = 6.5 Hz, 1H), 3.95 (q, *J* = 6.6 Hz, 1H), 2.18 (s, 3H), 2.18 (s, 3H), 2.14 (s, 3H), 2.11 (s, 3H), 2.03 (s, 3H), 2.01 (s, 3H), 2.00 (s, 3H), 1.99 (s, 3H), 1.22 (d, *J* = 6.3 Hz, 3H), 1.16 (d, *J* = 6.5 Hz, 3H).

1,2,3,4-Tetra-*O*-acetyl-L-rhamnopyranose 1m

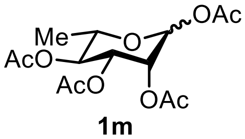

1m

Compound **1m** was synthesized according to general procedure B.7. using L-rhamnose monohydrate (1.00 g, 5.49 mmol, 1 equiv.), acetic anhydride (2.85 mL, 30.2 mmol, 5.5 equiv.) in anhydrous pyridine (12 mL). The product **1m** was obtained as a mixture of anomers in a 1:4.2 α:β ratio as a yellow syrup (1.59 g, 87% yield). The spectroscopic data are consistent with those previously reported.[21]

^{1}H NMR (400 MHz, $CDCl_3$, anomer mixture) δ 6.01 (d, *J* = 1.9 Hz, 1H), 5.83 (d, *J* = 1.3 Hz, 1H), 5.47 (bs, 1H), 5.30 (dd, *J* = 10.0, 3.6 Hz, 1H), 5.27 – 5.23 (m, 1H), 5.12 (t, *J* = 10.3 Hz, 1H), 5.09 – 5.06 (m, 2H), 3.93 (dq, *J* = 9.9, 6.2 Hz, 1H), 3.71 – 3.62 (m, 1H), 2.21 (s, 3H), 2.16 (s, 3H), 2.15 (s, 3H), 2.09 (s, 3H), 2.06 (s, 6H), 2.00 (s, 6H), 1.29 (d, *J* = 6.2 Hz, 3H), 1.23 (d, *J* = 6.2 Hz, 3H).

B.8. Synthetic procedure for methyl 2,4,7,8,9-penta-*O*-acetyl-5-(*N*-acetylacetamido)-3,5-dideoxy-D-*glycero*-β-D-*galacto*-non-2-ulopyranosonate 1p

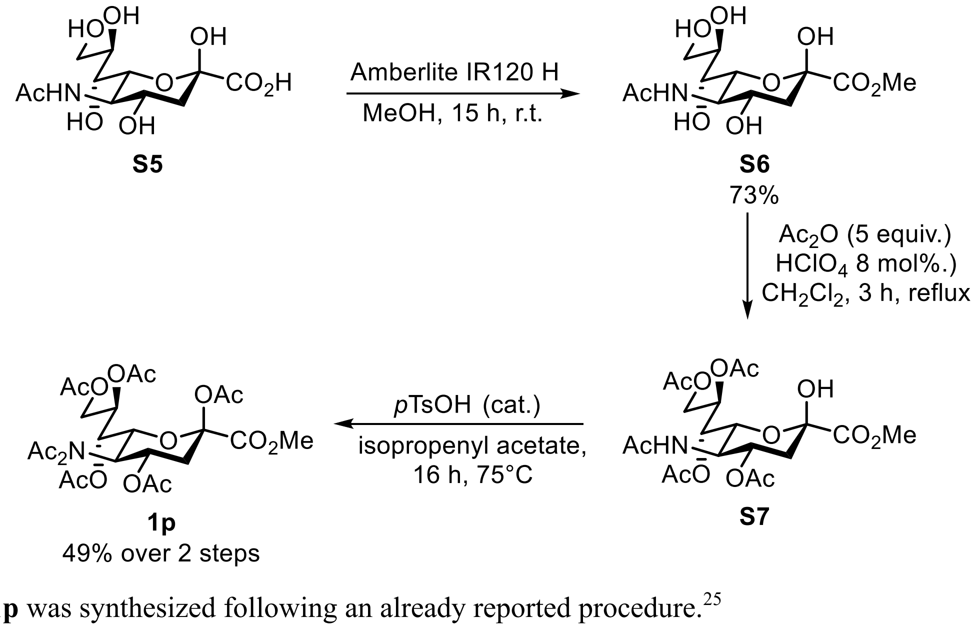

Compound **1p** was synthesized following an already reported procedure.[25]

Step 1: *N*-Acetylneuraminic acid **S5** (5 g, 16.2 mmol) and Amberlite™ IR-120 ion exchange resin (H^+ form, 5 g) were suspended in MeOH (75 mL) and stirred at room temperature for 15 h. The resin was removed by filtration and washed with MeOH (2×10 mL). The combined filtrates were concentrated under reduced

pressure to induce crystallization. The resulting solid was collected by filtration to afford *N*-acetylneuraminic acid methyl ester **S6** (3.8 g, 73%). The spectroscopic data are consistent with those previously reported.[25]

^{1}H NMR (400 MHz, D_2O) δ 4.82 – 4.71 (m, 1H), 4.13 – 4.00 (m, 2H), 3.93 (t, *J* = 10.1 Hz, 1H), 3.85 (s, 3H), 3.74 (t, *J* = 8.8 Hz, 1H), 3.63 (dd, *J* = 12.3, 5.8 Hz, 1H), 3.56 (d, *J* = 9.5 Hz, 1H), 2.32 (dd, *J* = 12.6, 4.7 Hz, 1H), 2.06 (s, 3H), 1.93 (t, *J* = 12.3 Hz, 1H).

Step 2: *N*-Acetylneuraminic acid methyl ester **S6** (2 g, 6.4 mmol) was suspended in CH_2Cl_2 (7.5 mL), and Ac_2O (3 mL, 32 mmol, 5.0 equiv.) was added. The mixture was stirred at room temperature, 70% $HClO_4$ (42 μL) was added dropwise over 30 min, during which the reaction mixture warmed to reflux. The mixture was stirred at reflux for 2.5 h, then MeOH (350 μL, 8.0 mmol) was added dropwise at room temperature, and stirring was continued for 1 h. The reaction mixture was diluted with CH_2Cl_2 (10 mL) and washed with H_2O (3×10 mL). The combined aqueous layers were extracted with CH_2Cl_2 (2×10 mL). The combined organic layers were washed with saturated $NaHCO_3$ aqueous solution (2×10 mL), dried over anhydrous Na_2SO_4, filtered, and the solvent removed under reduced pressure to afford a residue. The crude was partially purified by column chromatography (gradient $CHCl_3$:MeOH from 99:1 to 97:3). Compound **S7** was obtained as a mixture with unidentified side-products (2.1 g) and was used in the next step without further purification.

Step 3: To a suspension of crude **S7** (2 g) in isopropenyl acetate (6 mL) was added *p*-TsOH (700 mg, 0.4 mmol). The reaction mixture was stirred at 75°C for 16 h. Then, the mixture was cooled to room temperature, diluted with EtOAc (20 mL), and washed sequentially with saturated $NaHCO_3$ aqueous solution (10 mL), H_2O (20 mL), and brine (10 mL). The organic layer was dried over anhydrous Na_2SO_4, filtered, and the solvent evaporated under reduced pressure. The crude was purified by column chromatography (gradient toluene:acetone from 80:20 to 60:40) to give **2q** as a white solid (1.67 g, 49% over two steps). The spectroscopic data are consistent with those previously reported.[25]

^{1}H NMR (400 MHz, $CDCl_3$) δ 5.79 (td, *J* = 10.5, 5.1 Hz, 1H), 5.19 – 5.06 (m, 3H), 4.38 (d, *J* = 12.7 Hz, 1H), 4.20 (t, *J* = 10.4 Hz, 1H), 4.14 (dd, *J* = 12.4, 5.9 Hz, 1H), 3.79 (s, 3H), 2.68 (dd, *J* = 13.5, 5.4 Hz, 1H), 2.40 (s, 3H), 2.32 (s, 3H), 2.19 (s, 3H), 2.12 – 2.03 (m, 1H), 2.13 (s, 3H), 2.06 (s, 3H), 2.02 (s, 3H), 1.99 (s, 3H).

B.9. Synthetic procedure for 2,3,6,2',3',4',6'-hepta-*O*-acetyl-α-D-cellobiosyl iodide 2q

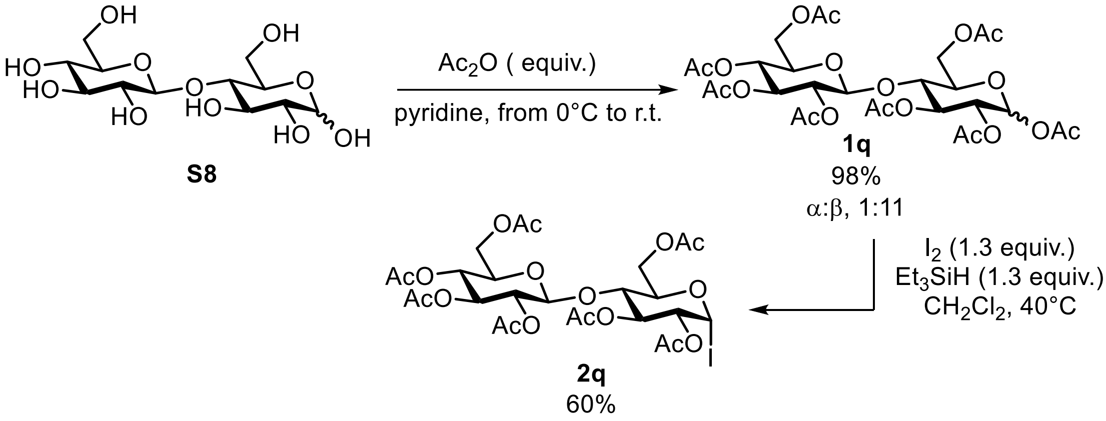

Step 1: 1,2,3,6,2',3',4',6'-octa-*O*-acetyl-β-D-cellobiose **1q** was prepared adapting an already reported procedure.[21] To a round bottom flask equipped with a magnetic stir bar **S8** (1.0 g, 2.9 mmol) was added and suspended in anhydrous pyridine (20 mL). The mixture was cooled down to 0°C and acetic anhydride (2.9 mL, 31 mmol, 10.4 equiv.) was added dropwise. The reaction was allowed to reach r.t. and stirred for 16 h. Then the mixture was quenched by adding water (20 mL) and stirred for 15 minutes. The mixture was poured in an extraction funnel and extracted with CH_2Cl_2 (3 x 10 mL), then the organic fraction was washed with an HCl 2 M aqueous solution (3 x 10 mL), saturated $CuSO_4$ aqueous solution (2 x 10 mL), H_2O (1 x 20 mL), saturated Na_2CO_3 aqueous solution (3 x 10 mL), and brine (1 x 10 mL). The organic layer was then dried with anhydrous $MgSO_4$, filtered, and concentrated *in vacuo* to give **1q** as a mixture of anomers in a 1:11 α:β ratio as a white solid (1.91 g, 98% yield), that was used without further purification. The spectroscopic data are consistent with those previously reported.[26]

^{1}H NMR (400 MHz, $CDCl_3$, main β anomer) δ 5.66 (d, J = 8.2 Hz, 1H), 5.23 (t, J = 9.0 Hz, 1H), 5.14 (t, J = 9.3 Hz, 1H), 5.10 – 5.01 (m, 2H), 4.92 (t, J = 8.7 Hz, 1H), 4.49 (t, J = 10.0 Hz, 2H), 4.36 (dd, J = 12.2, 4.5 Hz, 1H), 4.17 – 4.08 (m, 1H), 4.05 (d, J = 12.4 Hz, 1H), 3.82 (t, J = 9.4 Hz, 1H), 3.78 – 3.71 (m, 1H), 3.66 (d, J = 9.7 Hz, 1H), 2.12 (s, 3H), 2.09 (s, 6H), 2.03 (s, 9H), 2.01 (s, 3H), 1.98 (s, 3H).

Step 2: Compound **2q** was prepared following an already reported procedure.[27] To a round bottom flask equipped with a magnetic stir bar **1q** (1.0 g, 1.47 mmol, 1 equiv.) was added. The flask was evacuated and backfilled with nitrogen three times, then anhydrous CH_2Cl_2 (7.3 mL) was added, followed by iodine (485 mg, 1.9 mmol, 1.3 equiv.), and triethylsilane (310 μL, 1.9 mmol, 1.3 equiv.). The solution was stirred at 40°C for 3 h, then cooled down to room temperature, diluted with CH_2Cl_2 (100 mL), poured into a separatory funnel and washed with a 1:1 v/v mixture of saturated $NaHCO_3$ and a 10% w/v Na_2SO_3 aqueous solutions (100 mL in total) and then with H_2O (50 mL). The organic phase was dried over anhydrous Na_2SO_4, filtered, the solvent was removed under reduced pressure and the crude was purified by column chromatography (gradient Hex:EtOAc from 66:33 to 40:60) to give **2q** as a white solid (659 mg, 60%). The characterization data are in agreement with those previously reported.[28]

^{1}H NMR (400 MHz, $CDCl_3$) δ 6.92 (d, *J* = 4.4 Hz, 1H), 5.44 (t, *J* = 9.5 Hz, 1H), 5.15 (t, *J* = 9.3 Hz, 1H), 5.08 (t, *J* = 9.6 Hz, 1H), 4.94 (t, *J* = 8.5 Hz, 1H), 4.55 (d, *J* = 8.0 Hz, 1H), 4.52 (dd, *J* = 12.6, 2.1 Hz, 1H), 4.37 (dd, *J* = 12.5, 4.4 Hz, 1H), 4.18 (dd, *J* = 12.4, 4.2 Hz, 1H), 4.15 (dd, *J* = 10.1, 4.5 Hz, 1H), 4.06 (dd, *J* = 12.6, 2.3 Hz, 1H), 4.00 – 3.93 (m, 1H), 3.87 (t, *J* = 9.7 Hz, 1H), 3.68 (ddd, *J* = 9.8, 4.5, 2.3 Hz, 1H), 2.13 (s, 3H), 2.09 (s, 3H), 2.09 (s, 3H), 2.05 (s, 3H), 2.04 (s, 3H), 2.01 (s, 3H), 1.99 (s, 3H).

B.10. Preparation of [1.1.1]propellane (3) solution in Et_2O

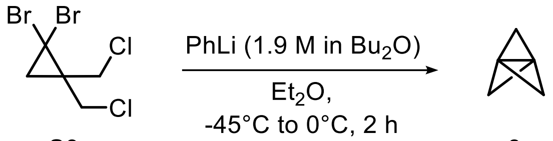

S9 **3**

The [1.1.1]propellane solution **3** was prepared following a described procedure:[29] a 250 mL flame dried round-bottom flask was charged with 1,1-dibromo-2,2-bis(chloromethyl)cyclopropane **S9** (10 gr, 33.6 mmol, 1.0 equiv.). The flask was evacuated and back-filled with nitrogen three times, and then anhydrous Et_2O (20 mL) was added. The solution was cooled to -45ºC in a dry ice/acetonitrile bath. Then, PhLi (1.9 M in Bu_2O, 35.6 mL, 67.4 mmol, 2.0 equiv.) was added dropwise with a syringe pump in 15 min (rate of addition: 2.37 mL/min) at -45ºC, and the resulting reaction mixture was stirred at the same temperature for 15 min. Then the cooling bath was replaced with an ice bath, and the reaction mixture was stirred at 0ºC for 2 h. The mixture was then distilled under vacuum at room temperature directly connecting the flask to a receiving vacuum trap immersed in a dry ice/isopropanol bath at -78°C. The co-distilled [1.1.1]propellane **3** was obtained as a solution in Et_2O:Bu_2O = 10:1 mixture (volume of distillate = 21.5 mL, [**3**] = 0.88 M, 56% yield). The solution was transferred to a vial and stored at -20ºC. No decomposition was observed over a period of 2 months. Occasionally, after prolonged storage at -20ºC or keeping the solution at room temperature for more than 30 min, the formation of a white precipitate was observed. However, in these cases no change in the ^{1}H NMR spectrum nor in the concentration of the [1.1.1]propellane **3** solution was observed. The white precipitate can be removed by simple filtration through a PTFE 0.45 μm filter. The concentration of [1.1.1]propellane **3** was determined by ^{1}H NMR in $CDCl_3$ using trichloroethylene as internal standard. Generally, $CDCl_3$ (0.5 mL), trichloroethylene (18 μL, 0.2 mmol), and an aliquot of the [1.1.1]propellane solution **3** (20 μL) were added in this order to an NMR tube. The ratio between internal standard (δ 6.45 ppm, 1H) and [1.1.1]propellane (δ 2.01 ppm, 6H) was used to determine the concentration of **3**. The concentration of [1.1.1]propellane **3** solutions used in this work ranged between 0.75 – 1.01 M. The spectroscopic data are in agreement with those reported in the literature.[29]

^{1}H NMR (400 MHz, $CDCl_3$ with Et_2O:Bu_2O = 10:1) δ 2.01 (s, 6H).

C. OPTIMIZATION OF THE REACTION CONDITIONS

Table S1. Optimization of the activation step[a]

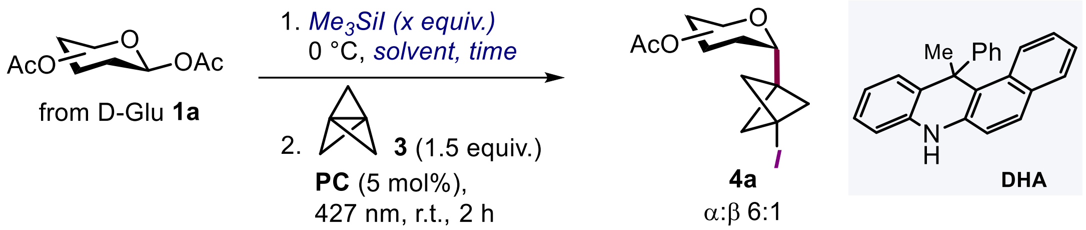

Entry	Time 1st step (h)	Me_3SiI (equiv.)	solvent	NMRy (%)[b]
1	0.5	1.0	CH_3CN	7
2	0.5	1.0	CH_2Cl_2	32
3	3	1.0	CH_2Cl_2	61
4	3	1.1	CH_2Cl_2	73
5	3	1.2	CH_2Cl_2	80
6	3	1.3	CH_2Cl_2	68
7	3	1.5	CH_2Cl_2	42
8	3	2.0	CH_2Cl_2	<5

[a]Reactions were performed using [1.1.1]propellane **3** as solution in Et_2O and 0.4 mL of solvent. [b]Determined by ^1H NMR analysis of the crude mixture using trichloroethylene as the internal standard. DHA: 12-methyl-12-phenyl-7,12-dihydrobenzo[*a*]acridine; r.t.: room temperature.

Comment: From this screening we observed a strong dependence on the activation time and most importantly on the stoichiometry of Me_3SiI.

Table S2. Evaluation of further parameters[a]

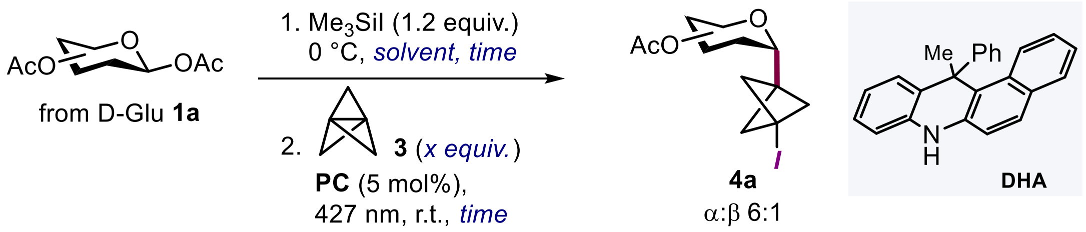

Entry	Time 1st step (h)	Time 2nd step (h)	3 (equiv.)	solvent	NMRy (%)[b]
1	3	2	1.5	CH_2Cl_2	80
2	3	2	2.0	CH_2Cl_2	77
3	3	2	2.5	CH_2Cl_2	69
4	2	2	1.5	CH_2Cl_2	79
5	1	2	1.5	CH_2Cl_2	75
6	0.5	2	1.5	CH_2Cl_2	63
7	3	1	1.5	CH_2Cl_2	52

8	3	0.5	1.5	CH_2Cl_2	9
9	3	2	1.5	CH_3CN	7
10	3	2	1.5	THF	<5
11	3	2	1.5	EtOAc	8
12	3	2	1.5	Toluene	47
13	3	2	1.5	DCE	>95

[a]Reactions were performed using [1.1.1]propellane **3** as solution in Et_2O and 0.4 mL of solvent. [b]Determined by ^1H NMR analysis of the crude mixture using trichloroethylene as the internal standard. DCE: 1,2-dichloroethane; DHA: 12-methyl-12-phenyl-7,12-dihydrobenzo[*a*]acridine; r.t.: room temperature.

*Comment: Increasing the amount of **3** or decreasing the reaction time was found to be detrimental. The reaction performed at best in chlorinated solvent, with DCE being the optimal one.*

Table S3. Control experiments[a]

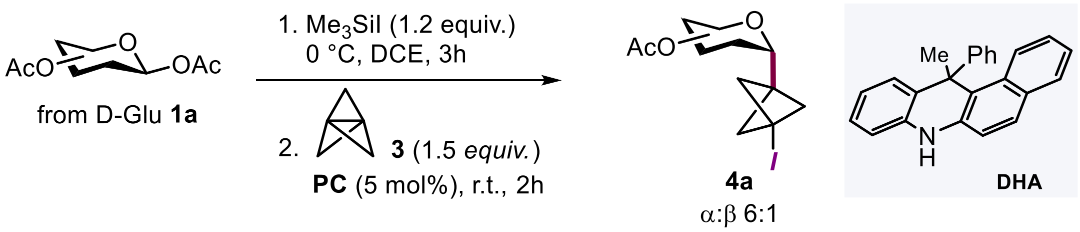

Entry	λ (nm)	PC	NMRy (%)[b]
1	427	**DHA**	>95
2	-	-	32
3[c]	-	-	42
4	390	-	50
5	427	-	71
6	427	*fac*-$Irppy_3$	36

[a]Reactions were performed using [1.1.1]propellane **3** as solution in Et_2O and 0.4 mL of solvent. [b]Determined by ^1H NMR analysis of the crude mixture using trichloroethylene as the internal standard. [c]Reaction performed in the absence of light at 40°C. DCE: 1,2-dichloroethane; DHA: 12-methyl-12-phenyl-7,12-dihydrobenzo[*a*]acridine; r.t.: room temperature.

*Comment: Control experiments revealed that, although the reaction can be initiated by thermal homolysis or by photolysis, optimal efficiency is achieved under photocatalytic conditions. The use of **DHA** enables to sustain an otherwise relatively inefficient radical chain propagation.*

*During our studies, we found that the ATRA addition to [1.1.1]propellane proceeds extremely rapidly when a pure sample of **2a** is used. In particular, quantitative formation of the addition product was observed within 15 min, regardless of whether the photocatalyst DHA was present. In contrast, when **2a** is generated in situ upon treatment of glycosyl ester **1a** with Me_3SiI, the formation of BCP C-glycoside **4a** proceeds significantly more slowly: after 15 min, only 20% yield of product was detected in the presence of DHA, while only trace*

amounts were detected in the absence of the photocatalyst. Although we are currently unable to identify the nature of the species responsible for this behavior, these observations suggest that inhibitors formed under the optimized conditions negatively affects the efficiency of chain propagation.

Table S4. Influence of the anomeric configuration of the substrates[a]

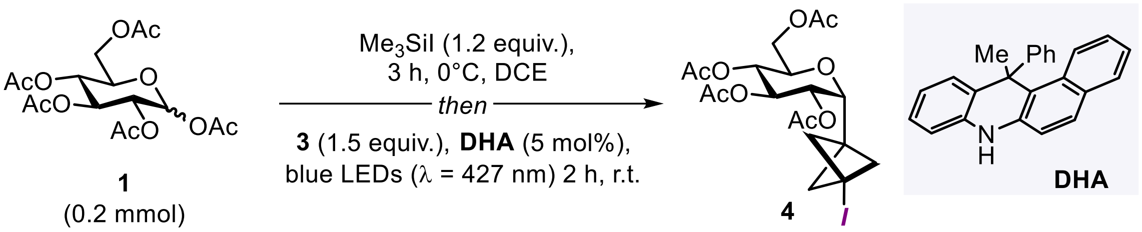

Entry	Anomeric configuration of 1	1st step reaction time (h)	Conversion of 1 after 1st step	4 (%)[b]
1	β	3	quant.	>98
2	α	3	49	17
3[c]	α	6	78	40

[a]Reactions were performed using [1.1.1]propellane **3** as solution in Et_2O and 0.4 mL of solvent. [b]Determined by ^{1}H NMR analysis of the crude mixture using trichloroethylene as the internal standard.

*Comment: these experiments demonstrate that the anomeric configuration of peracetylated glucose has a strong influence on its reactivity. On the other hand, our data suggest that this effect might be less pronounced for other types of substrates. Indeed, the related D-Man, D-Lyxo, and D-Rha (**1i,k,m**) all showed very good reactivity despite they differed significantly for their anomeric configuration (α:β, 5:1, 1:1, and 1:4 respectively).*

D. SYNTHESIS OF *C*-GLYCOSYL IODO BCPs

D.1. General procedure for the synthesis of *C*-glycosyl iodo BCPs 4 from unactivated saccharides

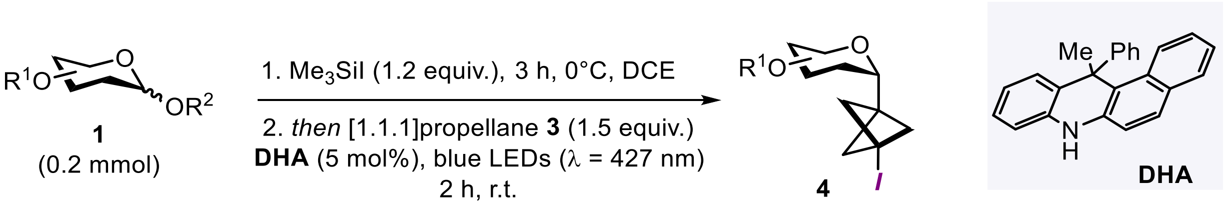

A 4 mL screw-cap vial with a PTFE/silicone septum and a magnetic stir bar was charged with glycoside **1** (0.2 mmol, 1 equiv.). The vial was sealed, evacuated under vacuum and back-filled with N_2 three times. Then N_2 sparged anhydrous 1,2-dichlorethane (0.25 M) was added, the vail was placed in an ice-water bath, and iodotrimethylsilane (1.2 equiv.) was added. The vial was sealed with parafilm, and the reaction mixture was

stirred at 0°C for 3 h, unless otherwise stated. Then, in a separate vial, **DHA** (5 mol%) was dissolved in a propellane **3** solution in Et_2O (1.5 equiv.) and the mixture was added to the reaction. The mixture was stirred for 2 h under the irradiation of a Kessil lamp PR160L (λ_{max} 427 nm, 45W, 50% intensity), unless otherwise stated (see set-up in Figure S2, Section A). After the irradiation period, the solvent was removed under reduced pressure and the crude mixture was analysed by ^{1}H NMR to determine the diastereomeric ratio. The crude product was purified by flash column chromatography on silica gel to afford the iodo BCP *C*-glycoside product **4** in the stated yield.

Characterization data:

1-(2,3,4,6-tetra-*O*-acetyl-α-D-glucopyranosyl)-3-iodobicyclo[1.1.1]pentane 4a

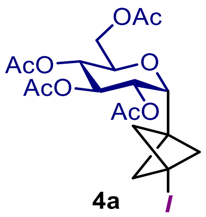

4a

Compound **4a** was synthesized according to general procedure D.1. using 1,2,3,4,6-penta-*O*-acetyl-β-D-glucopyranose **1a** (78 mg, 0.2 mmol), Me_3SiI (34 μL, 0.24 mmol), [1.1.1]propellane **3** solution (0.75 M in Et_2O, 400 μL, 0.3 mmol), **DHA** (3.2 mg, 0.01 mmol), and DCE (0.8 mL). Reaction time: 3 h then 2 h. The anomeric ratio was determined to be α:β, 6:1 by ^{1}H NMR analysis of the crude mixture, which was purified by flash column chromatography (eluent Hex:EtOAc, 80:20) to give compound **4a** as a yellow solid (93 mg, 89% yield). The spectroscopic data are in agreement with those reported in the literature.[30]

^{1}H NMR (400 MHz, $CDCl_3$, major α anomer) δ 5.30 (t, *J* = 9.1 Hz, 1H), 5.00 (dd, *J* = 9.5, 6.0 Hz, 1H), 4.87 (t, *J* = 9.0 Hz, 1H), 4.21 – 4.10 (m, 2H), 4.10 – 3.91 (m, 2H), 2.47 (d, *J* = 9.3 Hz, 3H), 2.38 (d, *J* = 9.3 Hz, 3H), 2.05 (s, 3H), 2.02 (s, 3H), 1.99 (s, 3H), 1.99 (s, 3H).

^{13}C NMR (101 MHz, $CDCl_3$) δ 170.6, 169.9, 169.5, 169.5, 71.0, 70.9, 70.4, 69.6, 68.5, 62.3, 61.2, 47.3, 20.8, 20.7, 20.7, 20.7, 5.8.

HRMS (ESI) *m*/*z*: $[M + Na]^+$ Calcd for $C_{19}H_{25}IO_9Na$ 547.0435; Found 547.0460.

2 mmol scale reaction – The reaction was performed in a 25 mL round bottom flask with minimum modifications of general procedure D.1 using 1,2,3,4,6-penta-*O*-acetyl-α-D-glucopyranose **1a** (780 mg, 2.00 mmol), Me_3SiI (340 μL, 2.40 mmol), [1.1.1]propellane **3** solution (1.01 M in Et_2O, 3.0 mL, 3.0 mmol), **DHA** (32 mg, 0.1 mmol), and DCE (8 mL). Reaction time: 6 h then 16 h. The mixture was irradiated using two Kessil lamps PR160L (λ_{max} 427 nm, 45W, 50% intensity) (see set-up in Figure S2, Section A). After the irradiation period, the solvent was removed under reduced pressure. The anomeric ratio was determined to be α:β, 6:1 by ^{1}H NMR analysis of the crude mixture, which was purified by flash column chromatography (eluent Hex:EtOAc, 80:20) to give compound **4a** as a yellow solid (540 mg, 51% yield).

1-iodo-3-(2,3,4,6-tetra-*O*-pivaloyl-α-D-glucopyranosyl)bicyclo[1.1.1]pentane 4b

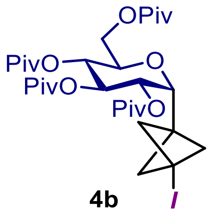

4b

Compound **4b** was synthesized according to general procedure D.1. using 1,2,3,4,6-penta-*O*-pivaloyl-β-D-glucopyranose **1b** (120 mg, 0.2 mmol), Me_3SiI (34 μL, 0.24 mmol), [1.1.1]propellane **3** solution (0.75 M in Et_2O, 400 μL, 0.3 mmol), **DHA** (3.2 mg, 0.01 mmol), and DCE (0.8 mL). Reaction time: 6 h then 2 h. The anomeric ratio was determined to be α:β, >20:1 by ^{1}H NMR analysis of the crude mixture, which was purified by flash column chromatography (gradient Hex:EtOAc from 98:2 to 90:10) to give compound **4b** as a white solid (102 mg, 74% yield).

^{1}H NMR (400 MHz, $CDCl_3$) δ 5.46 (t, *J* = 9.7 Hz, 1H), 5.09 (dd, *J* = 10.2, 6.2 Hz, 1H), 4.99 (t, *J* = 9.4 Hz, 1H), 4.18 (d, *J* = 6.1 Hz, 1H), 4.09 (d, *J* = 10.7 Hz, 1H), 4.04 – 3.95 (m, 2H), 2.54 (d, *J* = 9.4 Hz, 3H), 2.47 (d, *J* = 9.2 Hz, 3H), 1.22 (s, 9H), 1.18 (s, 9H), 1.16 (s, 9H), 1.13 (s, 9H).

^{13}C NMR (101 MHz, $CDCl_3$) δ 178.2, 177.4, 177.3, 176.7, 71.7, 71.3, 71.2, 69.6, 68.6, 62.8, 61.8, 47.6, 39.1, 39.0, 38.9, 38.9, 27.6, 27.4, 27.3, 27.2, 5.9.

HRMS (ESI) *m/z*: $[M + Na]^+$ Calcd for $C_{31}H_{49}IO_9Na$ 715.2313; Found 715.2334.

$\boldsymbol{\alpha_D^{25}}$= +74.5 (c 0.20, CH_2Cl_2)

1-(2,3,4,6-tetra-*O*-benzoyl-α-D-glucopyranosyl)-3-iodobicyclo[1.1.1]pentane 4c

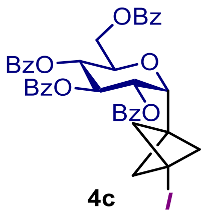

4c

Compound **4c** was synthesized with slight modification of general procedure D.1. A 4 mL screw-cap vial with a PTFE/silicone septum and a magnetic stir bar was charged with 1,2,3,4,6-penta-*O*-benzoyl-α-D-glucopyranose **1c** (140 mg, 0.2 mmol) and ZnI_2 (12 mg, 0.04 mmol, 20 mol%).[31] The vial was sealed, evacuated under vacuum and back-filled with N_2 three times. Then N_2 sparged anhydrous 1,2-dichlorethane (0.8 mL) was added, the vail was placed in an ice-water bath, and iodotrimethylsilane (30 μL, 0.2 mmol) was added. The vial was sealed with parafilm, and the reaction mixture was stirred at 0°C for 3 h, unless otherwise stated. Then, in a separate vial, **DHA** (3.2 mg, 0.01 mmol) was dissolved in a propellane **3** solution in Et_2O (0.75 M in Et_2O, 400 μL, 0.3 mmol) and the mixture was added to the reaction. The mixture was stirred for 2 h under the irradiation of a Kessil Lamp PR160L (λ_{max} 427 nm, 45W, 50% intensity) (see set-up in Figure S2, Section A). After the irradiation period, the solvent was removed under reduced pressure and the crude mixture was analyzed by ^{1}H NMR using trichloroethylene as internal standard revealing a 21% yield. The anomeric ratio could not be determined. Due to the low yield, the product was not purified.

1-(2,3,4,6-tetra-*O*-benzyl-α-D-glucopyranosyl)-3-iodobicyclo[1.1.1]pentane 4d

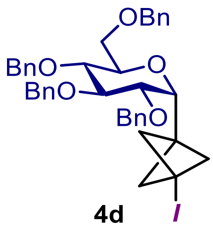

4d

Compound **4d** was synthesized according to general procedure D.1. using 1-*O*-acetyl-2,3,4,6-tetra-*O*-benzyl-α-D-glucopyranose **1d** (117 mg, 0.2 mmol), Me_3SiI (34 μL, 0.24 mmol), [1.1.1]propellane **3** solution (0.75 M in Et_2O, 400 μL, 0.3 mmol), **DHA** (3.2 mg, 0.01 mmol), and DCE (0.8 mL). Reaction time: 3 h then 2 h. The anomeric ratio was determined to be α:β, 8:1 by ^{1}H NMR analysis of the crude mixture, which was purified

by flash column chromatography (eluent Hex:EtOAc, 80:20) to give compound **4d** as a yellow oil (87 mg, 61% yield).

^{1}H NMR (400 MHz, CDCl$_3$) δ 7.36 – 7.26 (m, 18H), 7.14 (m, 2H), 4.93 (d, *J* = 10.9 Hz, 1H), 4.81 (d, *J* = 11.0 Hz, 2H), 4.65 (d, *J* = 11.5 Hz, 1H), 4.61 – 4.54 (m, 2H), 4.48 (dd, *J* = 11.5, 5.6 Hz, 2H), 4.04 (d, *J* = 5.5 Hz, 1H), 3.83 – 3.69 (m, 3H), 3.66 – 3.58 (m, 2H), 3.52 (dd, *J* = 9.8, 8.0 Hz, 1H), 2.50 (d, *J* = 9.3 Hz, 3H), 2.44 (d, *J* = 9.3 Hz, 3H).

^{13}C NMR (101 MHz, CDCl$_3$, one aromatic signal is overlapped) δ 138.6, 138.3, 138.1, 138.0, 128.6, 128.5, 128.5, 128.1, 128.03, 128.02, 127.99, 127.95, 127.9, 127.8, 127.8, 82.1, 80.6, 77.9, 75.6, 75.1, 73.7, 73.6, 73.5, 72.6, 69.1, 62.2, 48.3, 7.5.

HRMS (ESI) *m/z*: [M + Na]$^+$ Calcd for $C_{39}H_{41}IO_5Na$ 739.1891; Found 739.1875.

α_D^{25}= +58.2 (c 0.27, CH_2Cl_2)

1-iodo-3-(2,3,4,6-tetra-*O*-methyl-α-D-glucopyranosyl)bicyclo[1.1.1]pentane 4e

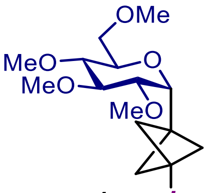

4e

Compound **4e** was synthesized according to general procedure D.1. using methyl 2,3,4,6-tetra-*O*-methyl-α-D-glucopyranoside **1e** (50 mg, 0.2 mmol), Me$_3$SiI (34 µL, 0.24 mmol), [1.1.1]propellane **3** solution (0.75 M in Et$_2$O, 400 µL, 0.3 mmol), **DHA** (3.2 mg, 0.01 mmol), and DCE (0.8 mL). Reaction time: 3 h then 2 h. The anomeric ratio was determined to be α:β, 12:1 by ^{1}H NMR analysis of the crude mixture, which was purified by flash column chromatography (gradient Hex: EtOAc from 80:20 to 70:30) to give compound **4e** as a brown solid (31 mg, 38% yield).

^{1}H NMR (400 MHz, CDCl$_3$) δ 4.05 (d, *J* = 5.7 Hz, 1H), 3.60 (s, 3H), 3.63 – 3.53 (m, 2H), 3.51 (s, 3H), 3.49 – 3.43 (m, 1H), 3.38 (s, 3H), 3.37 (s, 3H), 3.36 – 3.27 (m, 2H), 3.06 (t, *J* = 8.9 Hz, 1H), 2.48 (d, *J* = 9.2 Hz, 3H), 2.40 (d, *J* = 9.3 Hz, 3H).

^{13}C NMR (101 MHz, CDCl$_3$) δ 83.6, 82.3, 79.5, 73.3, 72.0, 71.7, 62.0, 60.8, 60.5, 59.4, 58.7, 48.3, 7.5.

HRMS (ESI) *m/z*: [M + Na]$^+$ Calcd for $C_{15}H_{25}IO_5Na$ 435.0639; Found 435.0630.

α_D^{25}= +89.4 (c 0.18, CH_2Cl_2)

1-α-D-glucopyranosyl-3-iodobicyclo[1.1.1]pentane 4f

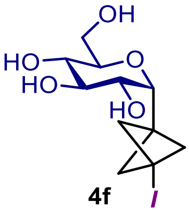

4f

Compound **4f** was synthesized with modification of general procedure D.1. using 1,2,3,4,6-penta-*O*-trimethylsilyl-D-glucopyranose **1f** (108 mg, 0.2 mmol), Me$_3$SiI (34 µL, 0.24 mmol), [1.1.1]propellane **3** solution (0.75 M in Et$_2$O, 400 µL, 0.3 mmol), **DHA** (3.2 mg, 0.01 mmol), and DCE (0.8 mL). Reaction time: 6 h then 2 h. The anomeric ratio was determined to be α:β, 14:1 by ^{1}H NMR analysis of the crude mixture. Then, the solvent was evaporated under reduced pressure, the residue was dissolved in MeOH (5 mL), trifluoroacetic acid (TFA) (300 µL, 0.4 mmol) was added dropwise, and the mixture stirred at r.t. for 2 h. Upon complete deprotection (assessed by TLC analysis) the solvent was removed under reduced pressure, and the crude mixture was

purified by flash column chromatography (gradient DCM:MeOH from 95:5 to 90:10) to give compound **4f** as a yellow oil (51 mg, 71% yield).

^{1}H NMR (400 MHz, CD_3OD) δ 3.90 (d, *J* = 5.6 Hz, 1H), 3.80 – 3.74 (m, 1H), 3.65 – 3.54 (m, 4H), 3.19 (t, *J* = 8.4 Hz, 1H), 2.49 (dd, *J* = 9.3, 1.7 Hz, 3H), 2.43 (d, *J* = 9.3, 1.7 Hz, 3H).

^{13}C NMR (101 MHz, CD_3OD) δ 76.9, 75.5, 74.9, 73.6, 72.0, 63.3, 63.1, 53.6, 8.6.

HRMS (ESI) *m*/*z*: $[M + Na]^+$ Calcd for $C_{11}H_{17}IO_5Na$ 379.0013; Found 379.0007.

α_D^{25} = +14.7 (c 0.55, MeOH)

1-iodo-3-(2,3:5,6-di-*O*-isopropylidene-α-D-mannofuranosyl)bicyclo[1.1.1]pentane 4g

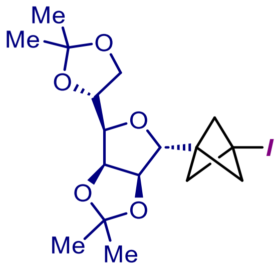

4g

Compound **4g** was synthesized according to general procedure D.1. using 1-*O*-acetyl-2,3:5,6-di-*O*-isopropylidene-α-D-mannofuranose **1g** (61 mg, 0.2 mmol), Me_3SiI (34 µL, 0.24 mmol), [1.1.1]propellane **3** solution (0.75 M in Et_2O, 400 µL, 0.3 mmol), **DHA** (3.2 mg, 0.01 mmol), and DCE (0.8 mL). Reaction time: 3 h then 2 h. The anomeric ratio was determined to be α:β, >20:1 by ^{1}H NMR analysis of the crude mixture, which was purified by flash column chromatography (eluent Hex:EtOAc, 90:10) to give compound **4g** as a brown oil (75 mg, 86% yield).

^{1}H NMR (400 MHz, $CDCl_3$) δ 4.73 (dd, *J* = 6.0, 3.7 Hz, 1H), 4.59 (dd, *J* = 6.1, 1.2 Hz, 1H), 4.32 (ddd, *J* = 7.5, 6.2, 4.4 Hz, 1H), 4.07 (dd, *J* = 8.7, 6.3 Hz, 1H), 4.03 – 3.96 (m, 2H), 3.75 (dd, *J* = 7.5, 3.8 Hz, 1H), 2.31 (dd, *J* = 9.4, 1.8 Hz, 3H), 2.25 (dd, *J* = 9.3, 1.8 Hz, 3H), 1.46 (s, 3H), 1.42 (s, 3H), 1.35 (s, 3H), 1.32 (s, 3H).

^{13}C NMR (101 MHz, $CDCl_3$) δ 113.1, 109.4, 83.4, 82.8, 81.9, 81.1, 73.5, 67.0, 59.4, 48.0, 27.0, 26.3, 25.2, 24.9, 6.6.

HRMS (ESI) *m*/*z*: Not found.

α_D^{25} = +18.6 (c 0.48, CH_2Cl_2)

1-iodo-3-(2,3:4,6-di-*O*-isopropylidene-α-D-mannopyranosyl)-1-iodobicyclo[1.1.1]pentane 4h

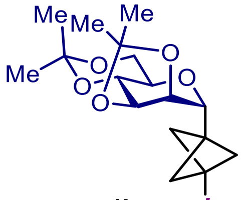

4h

Compound **4h** was synthesized according to general procedure D.1. using 1-*O*-acetyl-2,3:4,6-di-*O*-isopropylidene-α-D-mannopyranose **1h** (61 mg, 0.2 mmol), Me_3SiI (34 µL, 0.24 mmol), [1.1.1]propellane **3** solution (0.75 M in Et_2O, 400 µL, 0.3 mmol), **DHA** (3.2 mg, 0.01 mmol), and DCE (0.8 mL). Reaction time: 3 h then 2 h. The crude mixture was analyzed by ^{1}H NMR revealing a 75% yield with an anomeric ratio of α:β >20:1. The crude mixture was purified first by flash column chromatography (eluent Hex:EtOAc, 90:10) and then by HPLC (gradient H_2O:CH_3CN from 95:5 to 80:20) to give compound **4h** as a brown oil (38 mg, 44% yield).

^{1}H NMR (400 MHz, $CDCl_3$) δ 4.14 – 4.02 (m, 2H), 3.90 – 3.81 (m, 3H), 3.65 (t, *J* = 10.5 Hz, 1H), 3.36 (td, *J* = 10.3, 5.5 Hz, 1H), 2.35 (d, *J* = 9.2 Hz, 3H), 2.32 (d, *J* = 9.2 Hz, 3H), 1.49 (s, 3H), 1.49 (s, 3H), 1.41 (s, 3H), 1.34 (s, 3H).

^{13}C NMR (101 MHz, $CDCl_3$) δ 109.8, 99.7, 75.6, 74.4, 72.9, 72.4, 65.0, 62.8, 59.7, 48.5, 29.1, 27.9, 25.7, 19.0, 6.7.

HRMS (ESI) *m*/*z*: Not found.

α_D^{25}= +8.2 (c 0.29, CH_2Cl_2)

1-(2,3,4,6-tetra-*O*-acetyl-α-D-mannopyranosyl)-3-iodobicyclo[1.1.1]pentane 4i

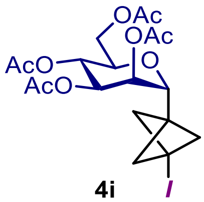

4i

Compound **4i** was synthesized according to general procedure D.1. using 1,2,3,4,6-penta-*O*-acetyl-D-mannopyranose **1i** (78 mg, 0.2 mmol), Me_3SiI (34 μL, 0.24 mmol), [1.1.1]propellane **3** solution (0.75 M in Et_2O, 400 μL, 0.3 mmol), **DHA** (3.2 mg, 0.01 mmol), and DCE (0.8 mL). Reaction time: 6 h then 2 h. The anomeric ratio was determined to be α:β, >20:1 by ^{1}H NMR analysis of the crude mixture, which was purified by flash column chromatography (eluent Hex:EtOAc, 80:20) to give compound **4i** as a brown oil (80 mg, 76% yield). The spectroscopic data are in agreement with those reported in the literature.[30]

^{1}H NMR (400 MHz, CDCl$_3$) δ 5.18 – 5.11 (m, 2H), 5.07 (t, *J* = 7.4 Hz, 1H), 4.38 (dd, *J* = 12.1, 7.1 Hz, 1H), 4.02 (dd, *J* = 12.1, 3.1 Hz, 1H), 3.98 – 3.91 (m, 2H), 2.41 (d, *J* = 9.3 Hz, 3H), 2.33 (d, *J* = 9.3 Hz, 3H), 2.07 (s, 3H), 2.06 (s, 3H), 2.06 (s, 3H), 2.02 (s, 3H).

^{13}C NMR (101 MHz, CDCl$_3$) δ 170.6, 170.0, 169.9, 169.7, 72.7, 72.6, 68.6, 68.4, 66.9, 62.1, 60.1, 47.2, 21.0, 20.9, 20.8, 20.8, 5.4.

HRMS (ESI) *m*/*z*: [M + Na]$^+$ Calcd for $C_{19}H_{25}IO_9Na$ 547.0435; Found 547.0460.

1-(2,3,4,6-tetra-*O*-acetyl-α-D-galactopyranosyl)-3-iodobicyclo[1.1.1]pentane 4j

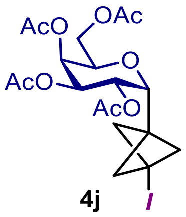

4j

Compound **4j** was synthesized according to general procedure D.1. using 1,2,3,4,6-penta-*O*-acetyl-D-galactopyranose **1j** (78 mg, 0.2 mmol), Me_3SiI (34 μL, 0.24 mmol), [1.1.1]propellane **3** solution (0.75 M in Et_2O, 400 μL, 0.3 mmol), **DHA** (3.2 mg, 0.01 mmol), and DCE (0.8 mL). Reaction time: 6 h then 2 h. The anomeric ratio was determined to be α:β, >20:1 by ^{1}H NMR analysis of the crude mixture, which was purified by flash column chromatography (eluent Hex:EtOAc, 80:20) to give compound **4j** as a brown oil (82 mg, 78% yield). The spectroscopic data are in agreement with those reported in the literature.[30]

^{1}H NMR (400 MHz, CDCl$_3$) δ 5.38 (d, *J* = 2.3 Hz, 1H), 5.22 (d, *J* = 2.6 Hz, 2H), 4.28 (dd, *J* = 11.5, 7.9 Hz, 1H), 4.24 – 4.20 (m, 1H), 4.17 (ddd, *J* = 7.6, 4.8, 2.8 Hz, 1H), 3.98 (dd, *J* = 11.4, 4.7 Hz, 1H), 2.43 (dd, *J* = 9.3, 1.8 Hz, 3H), 2.36 (dd, *J* = 9.3, 1.8 Hz, 3H), 2.10 (s, 3H), 2.08 (s, 3H), 2.06 (s, 3H), 2.03 (s, 3H).

^{13}C NMR (101 MHz, CDCl$_3$) δ 170.7, 170.1, 169.9, 169.8, 70.3, 70.2, 68.4, 67.4, 67.4, 61.5, 61.1, 47.2, 21.0, 20.9, 20.8, 20.7, 5.9.

HRMS (ESI) *m*/*z*: [M + Na]$^+$ Calcd for $C_{19}H_{25}IO_9Na$ 547.0435; Found 547.0460.

1-(2,3,4-tri-*O*-acetyl-α-D-xylopyranosyl)-3-iodobicyclo[1.1.1]pentane 4k

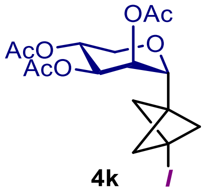

4k

Compound **4k** was synthesized according to general procedure D.1. using 1,2,3,4-tetra-*O*-acetyl-D-lyxopyranose **1k** (64 mg, 0.2 mmol), Me_3SiI (34 μL, 0.24 mmol), [1.1.1]propellane **3** solution (0.75 M in Et_2O, 400 μL, 0.3 mmol), **DHA** (3.2 mg, 0.01 mmol), and DCE (0.8 mL). Reaction time: 3 h then 2 h. The anomeric ratio was determined to be α:β, >20:1 by ^{1}H NMR analysis of the crude mixture, which was purified by flash column chromatography (eluent Hex:EtOAc, 80:20) to give compound **4k** as a yellow oil (70 mg, 77% yield).

^{1}H NMR (400 MHz, $CDCl_3$) δ 5.27 (t, *J* = 3.6 Hz, 1H), 4.90 (dd, *J* = 9.8, 3.2 Hz, 1H), 4.81 – 4.76 (m, 1H), 3.85 (d, *J* = 13.1 Hz, 1H), 3.78 (d, *J* = 13.2 Hz, 1H), 3.64 (d, *J* = 9.8 Hz, 1H), 2.31 (d, *J* = 9.3 Hz, 3H), 2.21 (d, *J* = 9.2 Hz, 3H), 2.13 (s, 3H), 2.11 (s, 3H), 1.99 (s, 3H).

^{13}C NMR (101 MHz, $CDCl_3$, one CH_3 signal is overlapped) δ 169.9, 169.5, 169.4, 72.0, 69.3, 68.0, 66.9, 65.6, 59.2, 47.8, 21.2, 20.9, 6.7.

HRMS (ESI) *m*/*z*: [M + Na]$^+$ Calcd for $C_{16}H_{21}IO_7Na$ 475.0224; Found 475.0235.

α_D^{25}= −19.7 (c 0.40, CH_2Cl_2)

1-(2,3,4-tri-*O*-acetyl-α-L-fucopyranosyl)-3-iodobicyclo[1.1.1]pentane 4l

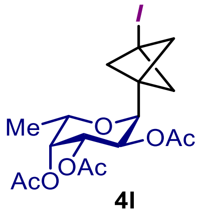

4l

Compound **4l** was synthesized according to general procedure D.1. using 1,2,3,4-tetra-*O*-acetyl-L-fucopyranose **1l** (67 mg, 0.2 mmol), Me_3SiI (34 μL, 0.24 mmol), [1.1.1]propellane **3** solution (0.75 M in Et_2O, 400 μL, 0.3 mmol), **DHA** (3.2 mg, 0.01 mmol), and DCE (0.8 mL). Reaction time: 3 h then 2h. The anomeric ratio was determined to be α:β, >20:1 by ^{1}H NMR analysis of the crude mixture, which was purified by flash column chromatography (eluent Hex:EtOAc, 80:20) to give compound **4l** as a brown oil (79 mg, 85% yield).

^{1}H NMR (400 MHz, $CDCl_3$) δ 5.32 – 5.20 (m, 3H), 4.18 (d, *J* = 5.6 Hz, 1H), 4.07 (q, *J* = 6.6 Hz, 1H), 2.48 (dd, *J* = 9.2, 1.7 Hz, 3H), 2.38 (dd, *J* = 9.2, 1.8 Hz, 3H), 2.14 (s, 3H), 2.06 (s, 3H), 2.00 (s, 3H), 1.12 (d, *J* = 6.4 Hz, 3H).

^{13}C NMR (101 MHz, $CDCl_3$) δ 170.6, 170.1, 169.9, 71.2, 70.6, 68.5, 68.0, 67.8, 61.6, 47.7, 21.0, 20.9, 20.8, 16.3, 6.0.

HRMS (ESI) *m*/*z*: [M + Na]$^+$ Calcd for $C_{17}H_{23}IO_7Na$ 489.0381; Found 489.0396.

α_D^{25}= −91.6 (c 0.37, CH_2Cl_2)

1-(2,3,4-tri-*O*-acetyl-α-L-rhamnopyranosyl)-3-iodobicyclo[1.1.1]pentane 4m

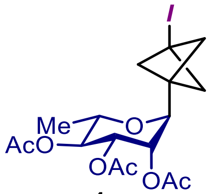

4m

Compound **4m** was synthesized according to general procedure D.1. using 1,2,3,4-tetra-*O*-acetyl-L-rhamnopyranose **1m** (67 mg, 0.2 mmol), Me_3SiI (34 μL, 0.24 mmol), [1.1.1]propellane **3** solution (0.75 M in Et_2O, 400 μL, 0.3 mmol), **DHA** (3.2 mg, 0.01 mmol), and DCE (0.8 mL). Reaction time: 3 h then 2 h. The anomeric ratio was determined to be α:β, >20:1 by ^{1}H NMR analysis of the crude mixture, which was purified

by flash column chromatography (eluent Hex:EtOAc, 80:20) to give compound **4m** as a brown oil (83 mg, 89% yield).

^{1}H NMR (400 MHz, CDCl$_3$) δ 5.18 (t, *J* = 3.2 Hz, 1H), 5.11 (dd, *J* = 8.9, 3.3 Hz, 1H), 4.99 (t, *J* = 8.4 Hz, 1H), 3.89 – 3.80 (m, 2H), 2.46 (d, *J* = 9.4 Hz, 3H), 2.39 (m, d, *J* = 9.4 Hz, 3H), 2.10 (s, 3H), 2.06 (s, 3H), 2.02 (s, 3H), 1.23 (d, *J* = 6.3 Hz, 3H).

^{13}C NMR (101 MHz, CDCl$_3$) δ 170.3, 170.3, 170.0, 73.8, 71.0, 70.4, 69.2, 69.0, 60.6, 47.5, 21.1, 21.0, 20.9, 17.7, 5.5.

HRMS (ESI) *m*/*z*: $[M + Na]^+$ Calcd for $C_{17}H_{23}IO_7Na$ 489.0381; Found 489.0359.

α_D^{25}= −66.4 (c 0.13, CH_2Cl_2)

1-(2,3-di-*O*-acetyl-5-deoxy–β-D-ribofuranosyl)-3-iodobicyclo[1.1.1]pentane 4n

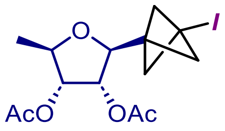

4n

Compound **4n** was synthesized according to general procedure D.1. using 1,2,3-tri-*O*-acetyl-5-deoxy-β-D-ribofuranose **1n** (52 mg, 0.2 mmol), Me_3SiI (34 µL, 0.24 mmol), [1.1.1]propellane **3** solution (0.75 M in Et_2O, 400 µL, 0.3 mmol), **DHA** (3.2 mg, 0.01 mmol), and DCE (0.8 mL). Reaction time: 3 h then 2 h. The anomeric ratio was determined to be α:β, 1:10 by ^{1}H NMR analysis of the crude mixture, which was purified by flash column chromatography (eluent Hex:EtOAc, 90:10) to give compound **4n** as a brown solid (34 mg, 43% yield).

^{1}H NMR (400 MHz, CDCl$_3$) δ 4.98 (t, *J* = 5.5 Hz, 1H), 4.70 (t, *J* = 5.9 Hz, 1H), 4.01 (quint., *J* = 6.2 Hz, 1H), 3.90 (d, *J* = 5.3 Hz, 1H), 2.31 (d, *J* = 9.3 Hz, 3H), 2.25 (d, *J* = 9.3 Hz, 3H), 2.06 (s, 3H), 2.05 (s, 3H), 1.26 (d, *J* = 6.5 Hz, 3H).

^{13}C NMR (101 MHz, CDCl$_3$) δ 170.0, 169.8, 79.7, 77.2, 75.8, 72.4, 58.5, 47.6, 20.8, 20.7, 19.0, 7.1.

HRMS (ESI) *m*/*z*: $[M + H]^+$ Calcd for $C_{14}H_{20}IO_5$ 395.0350; Found 395.0311.

α_D^{25}= −14.8 (c 0.18, CH_2Cl_2)

1-(2,3,5-tri-*O*-acetyl–β-D-ribofuranosyl)-3-iodobicyclo[1.1.1]pentane 4o

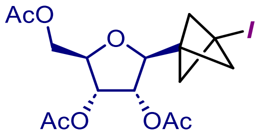

4o

Compound **4o** was synthesized according to general procedure D.1. using tetra-*O*-acetyl-β-D-ribofuranose **1o** (64 mg, 0.2 mmol), Me_3SiI (34 µL, 0.24 mmol), [1.1.1]propellane **3** solution (0.75 M in Et_2O, 400 µL, 0.3 mmol), **DHA** (3.2 mg, 0.01 mmol), and DCE (0.8 mL). Reaction time: 3 h then 2 h. The anomeric ratio was determined to be α:β, 1:13 by ^{1}H NMR analysis of the crude mixture, which was purified by flash column chromatography (eluent Hex:EtOAc, 80:20) to give compound **4o** as a brown oil (58 mg, 64% yield).

^{1}H NMR (400 MHz, CDCl$_3$) δ 5.03 (t, *J* = 5.3 Hz, 1H), 4.99 (t, *J* = 5.5 Hz, 1H), 4.27 (dd, *J* = 11.9, 3.0 Hz, 1H), 4.16 – 4.11 (m, 1H), 4.08 (dd, *J* = 11.9, 4.3 Hz, 1H), 3.97 (d, *J* = 5.6 Hz, 1H), 2.30 (dd, *J* = 9.4, 1.7 Hz, 3H), 2.24 (dd, *J* = 9.3, 1.7 Hz, 3H), 2.09 (s, 3H), 2.06 (s, 3H), 2.05 (s, 3H).

^{13}C NMR (101 MHz, CDCl$_3$) δ 170.6, 169.8, 169.7, 79.5, 79.2, 72.2, 71.7, 63.5, 58.4, 47.3, 20.9, 20.7, 20.7, 6.8.

HRMS (ESI) *m*/*z*: $[M + Na]^+$ Calcd for $C_{16}H_{21}IO_7Na$ 475.0224; Found 475.0213.

α_D^{25}= +14.7 (c 0.21, CH_2Cl_2)

1-iodo-3-(Methyl 4,7,8,9-tetra-*O*-acetyl-5-(*N*-acetylacetamido)-3,5-dideoxy-D-*glycero*-β-D-*galacto*-non-2-ulopyranosyl)onate)bicyclo[1.1.1]pentane 4p

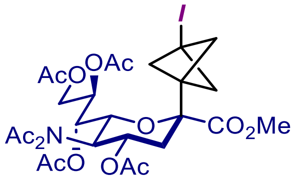

4p

Compound **4p** was synthesized according to general procedure D.1. using methyl 2,4,7,8,9-penta-*O*-acetyl-5-(*N*-acetylacetamido)-3,5-dideoxy-D-*glycero*-β-D-*galacto*-non-2-ulopyranosonate **1p** (115 mg, 0.2 mmol), Me_3SiI (34 μL, 0.24 mmol), [1.1.1]propellane **3** solution (0.75 M in Et_2O, 400 μL, 0.3 mmol), **DHA** (3.2 mg, 0.01 mmol), and DCE (0.8 mL). Reaction time: 6 h then 2 h. The crude mixture was analyzed by ^{1}H NMR revealing a 70% yield with an anomeric ratio of α:β, 1:>20. The crude mixture was purified first by flash column chromatography (gradient Hex:EtOAc from 80:20 to 60:40) and then by HPLC (gradient H_2O:CH_3CN from 95:5 to 70:30) to give compound **4p** as a yellow oil (75 mg, 53% yield).

^{1}H NMR (400 MHz, $CDCl_3$) δ 5.59 (td, *J* = 9.7, 4.9 Hz, 1H), 5.14 (bs, 2H), 4.96 (dd, *J* = 9.9, 1.6 Hz, 1H), 4.68 (d, *J* = 6.16 Hz, 1H), 4.17 – 4.03 (m, 2H), 3.73 (s, 3H), 2.50 (dd, *J* = 13.8, 5.0 Hz, 1H), 2.43 (dd, *J* = 9.4, 1.8 Hz, 3H), 2.41 – 2.34 (m, 6H), 2.27 (s, 3H), 2.10 (s, 3H), 2.05 (s, 3H), 2.02 (s, 3H), 1.96 (s, 3H), 1.88 (dd, *J* = 13.8, 9.8 Hz, 1H).

^{13}C NMR (101 MHz, $CDCl_3$, one *C*H_3 signal is overlapped) δ 174.9, 173.5, 170.7, 170.4, 170.3, 170.0, 169.8, 78.6, 72.1, 70.1, 68.7, 67.5, 62.3, 60.1, 57.6, 52.6, 49.5, 34.9, 28.0, 25.9, 21.1, 21.0, 20.9, 4.1.

HRMS (ESI) *m*/*z*: $[M + Na]^+$ Calcd for $C_{27}H_{36}INO_{13}Na$ 732.1124; Found 732.1155.

α_D^{25}= +26.5 (c 0.24, CH_2Cl_2)

D.2. Synthesis of 1-(2,3,6,2',3',4',6'-hepta-*O*-acetyl-α-D-cellobiopyranosyl)-3-iodobicyclo[1.1.1]pentane 4q

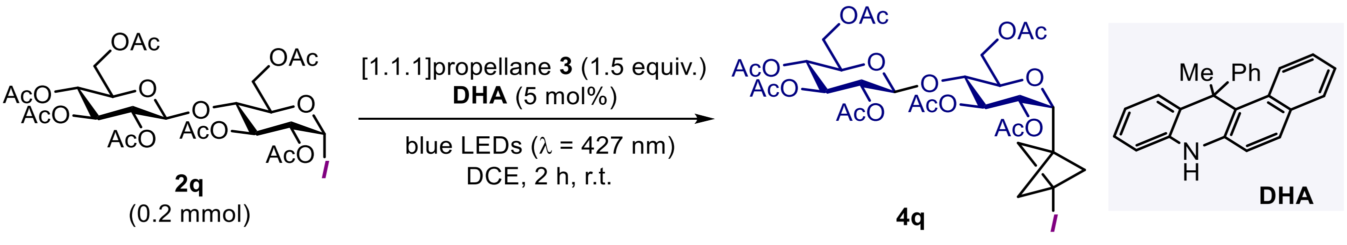

A 4 mL screw-cap vial with a PTFE/silicone septum and a magnetic stir bar was charged with glycoside 2,3,6,2',3',4',6'-hepta-*O*-acetyl-α-D-cellobiosyl iodide **2q** (149 mg, 0.2 mmol) and **DHA** (3.2 mg, 0.01 mmol, 5 mol%). The vial was sealed, evacuated under vacuum and back-filled with N_2 three times. Then N_2 sparged anhydrous 1,2-dichlorethane (800 μL) and a propellane **3** solution in Et_2O (1.01 M in Et_2O, 300 μL, 0.3 mmol, 1.5 equiv.) were added. The vial was sealed with parafilm, and the reaction mixture was stirred for 2 h under the irradiation of a Kessil Lamp PR160L (λ_{max} 427 nm, 45W, 50% intensity) (see set-up in Figure S2, Section A). The crude mixture was analyzed by ^{1}H NMR revealing a 71% yield and a α:β, 5:1 anomeric ratio. The crude mixture was purified by flash column chromatography (eluent Hex: EtOAc 70%:30%) to give compound

4q as a yellow solid (86 mg, 53% yield) isolated along with 18% of protodeiodinated *C*-glycosyl BCP. The spectroscopic data are in agreement with those reported in the literature.[30]

^{1}H NMR (400 MHz, CDCl$_3$) δ 5.34 (t, *J* = 7.5 Hz, 1H), 5.15 (t, *J* = 9.4 Hz, 1H), 5.06 (t, *J* = 9.6 Hz, 1H), 5.00 – 4.87 (m, 2H), 4.54 (dd, *J* = 8.1, 3.3 Hz, 1H), 4.40 – 4.26 (m, 2H), 4.16 – 4.00 (m, 3H), 3.91 (ddd, *J* = 8.8, 6.5, 2.6 Hz, 1H), 3.68 (ddd, *J* = 10.3, 4.6, 2.4 Hz, 1H), 3.56 (t, *J* = 7.7 Hz, 1H), 2.45 (dd, *J* = 9.3, 1.8 Hz, 3H), 2.37 (dd, *J* = 9.3, 1.9 Hz, 3H), 2.11 (s, 3H), 2.08 (s, 3H), 2.06 (s, 2H), 2.04 (s, 7H), 2.01 (s, 3H), 1.98 (s, 3H).

^{13}C NMR (101 MHz, CDCl$_3$) δ 170.7, 170.5, 170.4, 169.9, 169.6, 169.5, 169.3, 101.2, 76.8, 73.1, 72.2, 72.1, 71.7, 70.5, 69.9, 68.9, 68.0, 62.4, 61.8, 61.0, 51.9, 47.2, 20.9, 20.9, 20.8, 20.8, 20.7, 20.7, 20.7, 6.2.

D.3. Unsuccessful substrates

Generally, the following substrates gave no products or only trace amounts of product (below 5% yield), as judged by ^{1}H NMR. A few substrates gave products in low yields, as indicated in the Scheme.

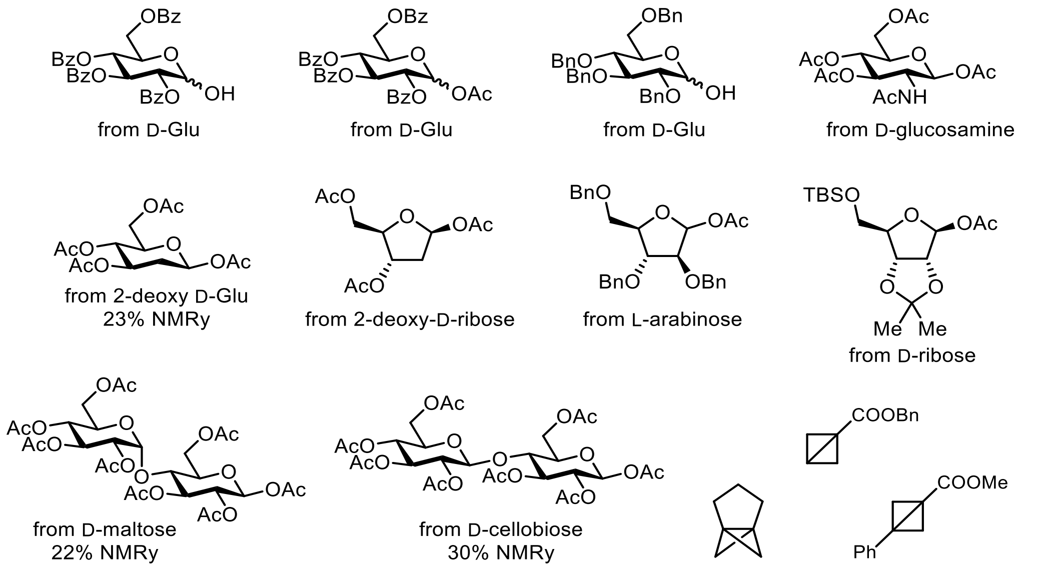

F. REACTION SCALE UP AND PRODUCT DERIVATIZATIONS

F.1. General procedure for derivatization via photocatalysis

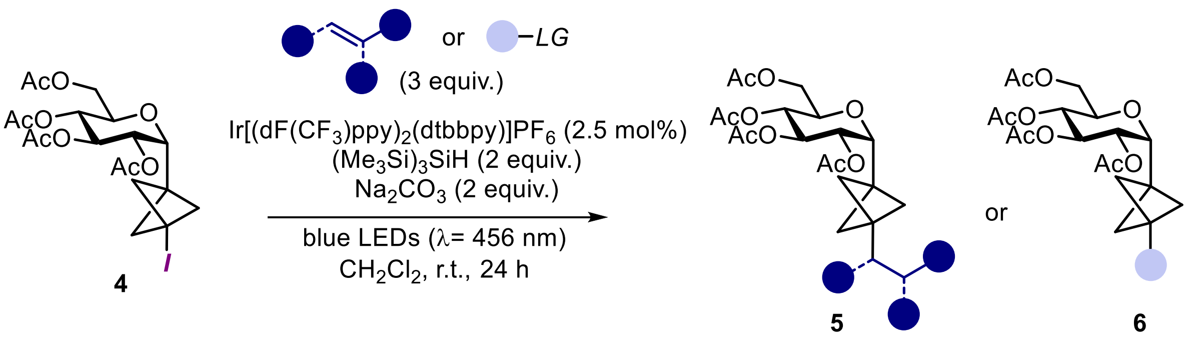

A 4 mL screw-cap vial equipped with a magnetic stir bar was charged with 1-(2,3,4,6-tetra-*O*-acetyl-α-D-glucopyranosyl)-3-iodobicyclo[1.1.1]pentane **4a** (0.10 mmol, 1.0 equiv., α:β, 6:1), radical acceptor (3.0

equiv.), Ir[(dF(CF_3)ppy)$_2$(dtbbpy)]PF_6 (2.5 mol%), and Na_2CO_3 (0.20 mmol, 2.0 equiv.). The vial was sealed with a PTFE/silicone septum, evacuated under vacuum, and back-filled with N_2 three times. Anhydrous CH_2Cl_2 (0.15 M), previously sparged with N_2, was added, followed by $(Me_3Si)_3SiH$ (0.20 mmol, 2.0 equiv.). The vial was sealed with parafilm and the reaction mixture was stirred at room temperature for 24 h under irradiation with blue LEDs (λ_{max} = 456 nm) (see set-up in Figure S2, Section A). After completion, the solvent was removed under reduced pressure and the crude mixture was purified by flash column chromatography on silica gel to afford the corresponding functionalized *C*-glycosyl BCP compounds in the stated yield.

Dimethyl 2-(3-(2,3,4,6-tetra-*O*-acetyl-α-D-glucopyranosyl)bicyclo[1.1.1]pentan-1-yl)succinate 5a

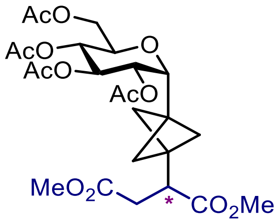

5a
1:1 d.r.

Compound **5a** was synthesized by modification of general procedure F.1. using 1-(2,3,4,6-tetra-*O*-acetyl-α-D-glucopyranosyl)-3-iodobicyclo[1.1.1]pentane **4a** (53 mg, 0.1 mmol), dimethyl fumarate (69 mg, 0.6 mmol), Ir[(dF(CF_3)ppy)$_2$(dtbbpy)]PF_6 (2.5 mol%, 2.8 mg), Na_2CO_3 (21 mg, 0.2 mmol), $(Me_3Si)_3SiH$ (62 µL, 0.2 mmol), and MeOH (600 µL) and H_2O (70 uL), both previously sparged with N_2. Reaction time: 24 h. The crude mixture was analysed by ^{1}H NMR to determine the d.r as 1:1 using trichloroethylene as internal standard. Then the solvent was removed under reduced pressure, and the crude residue was purified by flash column chromatography (gradient Hexane:EtOAc from 80:20 to 60:40) to give **5a** as a pale colorless oil (29 mg, 53% yield).

^{1}H NMR (400 MHz, $CDCl_3$, mixture of diastereoisomers) δ 5.40 (t, *J* = 9.3 Hz, 1H), 5.06 (dd, *J* = 9.8, 6.2 Hz, 1H), 4.93 (t, *J* = 9.1 Hz, 1H), 4.23 – 4.15 (m, 1H), 4.13 (d, *J* = 6.1 Hz, 1H), 4.10 – 3.98 (m, 2H), 3,72 (s, 3 H), 3,68 (s, 3 H), 3.03 (dd, *J* = 10.0, 5.0 Hz, 1H), 2.72 (dd, *J* = 16.8, 10.1 Hz, 1H), 2.40 (dd, *J* = 16.8, 5.0 Hz, 1H), 2.08 (s, 3H), 2.03 (bs, 9H), 1.89 (d, *J* = 9.5 Hz, 3H), 1.79 (d, *J* = 9.6 Hz, 3H).

^{13}C NMR (101 MHz, $CDCl_3$, mixture of diastereoisomers, two *C*=O, three CO_2CH_3, two CH_2, and seven $COCH_3$ are overlapped) δ 172.8, 172.7, 172.3, 170.8, 170.2, 170.2, 169.8, 169.8, 169.7, 71.0, 71.0, 70.8, 70.7, 70.7, 70.7, 70.1, 70.1, 68.9, 68.8, 62.5, 62.5, 52.1, 51.5, 42.7, 41.0, 38.4, 33.2, 20.9, 20.8, 20.80.

HRMS (ESI) *m/z*: $[M + H]^+$ Calcd for $C_{25}H_{35}O_{13}$ 543.2072; Found 543.2055.

α_D^{25}= +39.4 (c 0.46, CH_2Cl_2)

Methyl 2-(bis(tert-butoxycarbonyl)amino)-3-(3-(2,3,4,6-tetra-*O*-acetyl-α-D-glucopyranosyl)bicyclo[1.1.1]pentan-1-yl)propanoate 5b

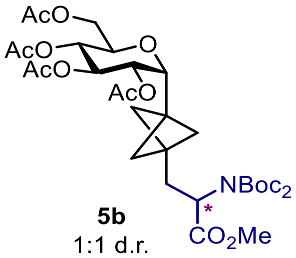

Compound **5b** was synthesized according to general procedure F.1. using 1-(2,3,4,6-tetra-*O*-acetyl-α-D-glucopyranosyl)-3-iodobicyclo[1.1.1]pentane **4a** (53 mg, 0.1 mmol), *N*,*N*-*tert*-butoxycarbonyldehydroalanine (91 mg, 0.3 mmol), Ir[(dF(CF_3)ppy)$_2$(dtbbpy)]PF_6 (2.5 mol%, 2.8 mg), Na_2CO_3 (21 mg, 0.2 mmol), $(Me_3Si)_3SiH$ (62 µL, 0.2 mmol), and CH_2Cl_2 (670 µL). Reaction time: 24 h. The crude mixture was analysed by ^{1}H NMR to determine the d.r as 1:1 using trichloroethylene as internal standard. Then the solvent was removed under reduced pressure, and the crude

residue was purified by flash column chromatography (eluent Hexane:EtOAc 70:30) to give **5b** as a yellow solid (36 mg, 51% yield).

^{1}H NMR (400 MHz, $CDCl_3$, mixture of diastereoisomers) δ 5.44 (td, *J* = 9.4, 3.3 Hz, 1H), 5.06 (dd, *J* = 9.9, 6.2 Hz, 1H), 4.98 – 4.88 (m, 2H), 4.22 – 4.09 (m, 2H), 4.08 – 4.00 (m, 2H), 3.71 (s, 3H), 2.36 (dd, *J* = 15.2, 4.7 Hz, 1H), 2.11 – 2.05 (m, 1H), 2.09 (s, 3H), 2.03 (s, 3H), 2.02 (s, 3H), 2.01 (s, 3H), 1.90 (t, *J* = 8.4 Hz, 3H), 1.82 – 1.76 (m, 3H), 1.51 (s, 18H).

^{13}C NMR (101 MHz, $CDCl_3$, mixture of diastereoisomers, one *C*=O, two *C*OOtBu, two *C*(CH_3)$_3$, three *C*H, one CH_2, two CO_2*C*H_3, three C(*C*H_3)$_3$ are overlapped) δ 171.4, 170.9, 170.8, 170.2, 170.1, 169.9, 169.9, 169.7, 169.7, 152.2, 152.1, 83.3, 83.3, 71.3, 71.1, 71.1, 70.5, 70.3, 69.0, 69.0, 62.6, 62.5, 56.4, 56.4, 52.8, 52.4, 39.8, 39.8, 39.2, 39.2, 31.7, 31.7, 28.2, 20.9, 20.9, 20.9, 20.8, 20.8, 20.8, 20.8.

HRMS (ESI) *m/z*: $[M + Na]^+$ Calcd for $C_{33}H_{49}NO_{15}Na$ 722.2994; Found 722.2921.

α_D^{25}= +24.4 (c 0.32, CH_2Cl_2)

***N*-((3-(2,3,4,6-tetra-*O*-acetyl-α-D-glucopyranosyl)bicyclo[1.1.1]pentan-1-yl)(phenyl)methyl)-4-methylbenzenesulfonamide 5c**

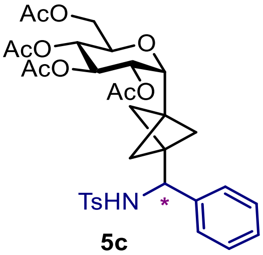

5c
1:1 d.r.

Compound **5c** was synthesized according to general procedure F.1. using 1-(2,3,4,6-tetra-*O*-acetyl-α-D-glucopyranosyl)-3-iodobicyclo[1.1.1]pentane **4a** (53 mg, 0.1 mmol), *N*-benzylidene-4-methylbenzenesulfonamide (78 mg, 0.3 mmol), Ir[(dF(CF_3)ppy)$_2$(dtbbpy)]PF_6 (2.5 mol%, 2.8 mg), Na_2CO_3 (21 mg, 0.2 mmol), $(Me_3Si)_3SiH$ (62 µL, 0.2 mmol), and CH_2Cl_2 (670 µL). Reaction time: 24 h. The crude mixture was analysed by ^{1}H NMR to determine the d.r as 1:1 using trichloroethylene as internal standard. Then the solvent was removed under reduced pressure, and the crude residue was purified by flash column chromatography (gradient Hexane: EtOAc, from 80:20 to 70:30) to give **5c** as a yellow solid (53 mg, 82% yield).

^{1}H NMR (400 MHz, $CDCl_3$, mixture of diastereoisomers) δ 7.54 – 7.48 (m, 2H), 7.19 – 7.12 (m, 3H), 7.09 (d, *J* = 8.4 Hz, 2H), 6.95 – 6.88 (m, 2H), 5.32 (t, *J* = 9.5 Hz, 1H), 5.12 – 4.98 (m, 2H), 4.91 (t, *J* = 9.2 Hz, 1H), 4.40 (dd, *J* = 7.7, 3.6 Hz, 1H), 4.14 (ddd, *J* = 12.6, 5.4, 2.7 Hz, 1H), 4.07 (d, *J* = 6.2 Hz, 1H), 3.98 (d, *J* = 12.2 Hz, 1H), 3.94 – 3.87 (m, 1H), 2.34 (s, 3H), 2.04 (s, 3H), 2.02 – 1.94 (m, 9H), 1.79 (d, *J* = 9.4 Hz, 1.5H), 1.74 – 1.67 (m, 3H), 1.60 (d, *J* = 9.2 Hz, 1.5H).

^{13}C NMR (101 MHz, $CDCl_3$, mixture of diastereoisomers, one *C*=O, two *C*H, one *C*q, one *C*H_3Ar, four CO_2*C*H_3 are overlapped) δ 170.8, 170.7, 170.1, 170.1, 169.7, 169.7, 169.6, 143.3, 143.3, 137.9, 137.8, 137.5, 137.5, 129.4, 129.4, 128.4, 127.5, 127.4, 127.2, 127.1, 126.7, 126.6, 70.9, 70.9, 70.8, 70.8, 70.6, 70.5, 70.1, 70.1, 68.7, 62.3, 62.3, 58.4, 58.4, 50.2, 50.1, 43.8, 43.7, 39.4, 21.5, 20.8, 20.8, 20.7, 20.7.

HRMS (ESI) *m/z*: $[M + H]^+$ Calcd for $C_{33}H_{40}NO_{11}S$ 658.2317; Found 658.2307.

α_D^{25}= +23.0 (c 0.20, CH_2Cl_2)

1-(2,3,4,6-tetra-*O*-acetyl-α-D-glucopyranosyl)-3-(2-(phenylsulfonyl)allyl)bicyclo[1.1.1]pentane 5d

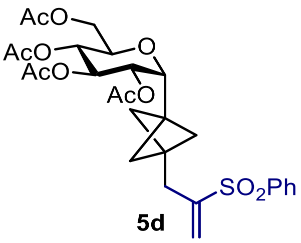

Compound **5d** was synthesized according to general procedure F.1. using 1-(2,3,4,6-tetra-*O*-acetyl-α-D-glucopyranosyl)-3-iodobicyclo[1.1.1]pentane **4a** (53 mg, 0.1 mmol), (prop-1-ene-2,3-diyldisulfonyl)dibenzene (97 mg, 0.3 mmol), Ir[(dF(CF_3)ppy)$_2$(dtbbpy)]PF_6 (2.5 mol%, 2.8 mg), Na_2CO_3 (21 mg, 0.2 mmol), $(Me_3Si)_3SiH$ (62 μL, 0.2 mmol), and CH_2Cl_2 (670 μL). Reaction time: 24 h. Then the solvent was removed under reduced pressure, and the crude residue was purified by flash column chromatography (gradient Hexane: EtOAc, from 90:10 to 70:30) to give **5d** as a yellow oil (27 mg, 47% yield).

^{1}H NMR (400 MHz, CDCl$_3$) δ 7.89 – 7.85 (m, 2H), 7.67 – 7.60 (m, 1H), 7.55 (t, *J* = 7.6 Hz, 2H), 6.43 (s, 1H), 5.75 (s, 1H), 5.37 (t, *J* = 9.4 Hz, 1H), 5.03 (dd, *J* = 9.9, 6.1 Hz, 1H), 4.92 (t, *J* = 9.2 Hz, 1H), 4.17 (dd, *J* = 12.1, 5.2 Hz, 1H), 4.08 (d, *J* = 6.2 Hz, 1H), 4.03 (dd, *J* = 12.1, 2.6 Hz, 1H), 3.99 (ddd, *J* = 9.5, 5.2, 2.5 Hz, 1H), 2.48 (s, 2H), 2.08 (s, 3H), 2.03 (s, 3H), 2.01 (s, 6H), 1.78 (d, *J* = 9.6 Hz, 3H), 1.68 (d, *J* = 9.5 Hz, 3H).

^{13}C NMR (101 MHz, CDCl$_3$) δ 170.8, 170.2, 169.8, 169.7, 147.7, 138.9, 133.8, 129.4, 128.6, 125.4, 71.1, 70.9, 70.5, 70.2, 68.9, 62.5, 52.6, 40.0, 39.0, 31.7, 20.9, 20.8, 20.8.

HRMS (ESI) *m/z*: $[M + H]^+$ Calcd for $C_{28}H_{35}O_{11}S$ 579.1895; Found 579.1888.

α_D^{25} = +43.3 (c 0.43, CH_2Cl_2)

2-(3-(2,3,4,6-tetra-*O*-acetyl-α-D-glucopyranosyl)bicyclo[1.1.1]pentan-1-yl)benzo[*d*]thiazole 6a

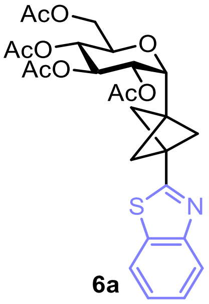

Compound **6a** was synthesized by modification of general procedure F.1. using 1-(2,3,4,6-tetra-*O*-acetyl-α-D-glucopyranosyl)-3-iodobicyclo[1.1.1]pentane **4a** (53 mg, 0.1 mmol), 2-(phenylsulfonyl)benzothiazole (83 mg, 0.3 mmol), Ir[(dF(CF_3)ppy)$_2$(dtbbpy)]PF_6 (2.5 mol%, 2.8 mg), Na_2CO_3 (21 mg, 0.2 mmol), $(Me_3Si)_3SiOH$ (62 μL, 0.2 mmol), and CH_2Cl_2 (670 μL). Reaction time: 24 h. Then the solvent was removed under reduced pressure, and the crude residue was purified by flash column chromatography (gradient Hexane: EtOAc, from 90:10 to 70:30) to give **6a** as a yellow solid (25 mg, 49% yield).

^{1}H NMR (400 MHz, CDCl$_3$) δ 8.01 (d, *J* = 8.1 Hz, 1H), 7.86 (d, *J* = 8.0 Hz, 1H), 7.47 (t, *J* = 8.2 Hz, 1H), 7.38 (t, *J* = 7.8 Hz, 1H), 5.47 (t, *J* = 9.2 Hz, 1H), 5.14 (dd, *J* = 9.6, 6.0 Hz, 1H), 4.98 (t, *J* = 8.9 Hz, 1H), 4.30 – 4.20 (m, 2H), 4.17 – 4.06 (m, 2H), 2.51 (d, *J* = 9.5 Hz, 3H), 2.41 (d, *J* = 9.5 Hz, 3H), 2.10 (s, 3H), 2.09 (s, 3H), 2.05 (s, 6H).

^{13}C NMR (101 MHz, CDCl$_3$) δ 170.8, 170.1, 169.8, 169.7, 168.6, 153.6, 135.4, 126.4, 125.3, 123.2, 121.8, 71.1, 70.9, 70.6, 70.1, 68.8, 62.6, 55.1, 41.2, 39.7, 20.9, 20.84, 20.81.

HRMS (ESI) *m/z*: $[M + H]^+$ Calcd for $C_{26}H_{30}NO_9S$ 532.1636; Found 532.1620.

α_D^{25} = +65.6 (c 0.63, CH_2Cl_2)

(3-(2,3,4,6-tetra-*O*-acetyl-α-D-glucopyranosyl)bicyclo[1.1.1]pentan-1-yl)(trifluoromethyl)sulfane 6b

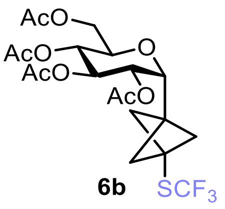

Compound **6b** was synthesized by modification of general procedure F.1. using 1-(2,3,4,6-tetra-*O*-acetyl-α-D-glucopyranosyl)-3-iodobicyclo[1.1.1]pentane **4a** (53 mg, 0.1 mmol), 2-((trifluoromethyl)thio)isoindoline-1,3-dione (74 mg, 0.3 mmol), Ir[(dF(CF$_3$)ppy)$_2$(dtbbpy)]PF$_6$ (2.5 mol%, 2.8 mg), Na_2CO_3 (21 mg, 0.2 mmol), $(Me_3Si)_3SiH$ (62 μL, 0.2 mmol), and CH_2Cl_2 (670 μL). Reaction time: 24 h. The crude mixture was analysed by ^{1}H NMR to assess the formation of **6b** in 63% yield. Then the solvent was removed under reduced pressure, and the crude residue was purified by flash column chromatography (gradient Hexane: EtOAc, from 90:10 to 80:20) and then by HPLC to give **6b** as a yellow solid (10 mg, 20% yield).

^{1}H NMR (400 MHz, CDCl$_3$) δ 5.34 (t, *J* = 9.0 Hz, 1H), 5.07 (dd, *J* = 9.4, 6.0 Hz, 1H), 4.93 (t, *J* = 8.9 Hz, 1H), 4.27 – 4.17 (m, 2H), 4.08 – 3.98 (m, 2H), 2.33 (d, *J* = 9.5 Hz, 3H), 2.24 (d, *J* = 9.5 Hz, 3H), 2.09 (s, 3H), 2.06 (s, 3H), 2.03 (s, 3H), 2.03 (s, 3H).

^{13}C NMR (101 MHz, CDCl$_3$) δ 170.7, 170.1, 169.7, 169.6, 130.5 (q, *J* =307.1 Hz), 71.1, 70.1, 70.3, 69.9, 68.6, 624, 57.0, 41.6 (q, *J* =1.3 Hz), 38.5 (q, *J* =1.7 Hz), 20.8, 20.8, 20.8.

^{19}F NMR (377 MHz, CDCl$_3$) δ -39.2.

HRMS (ESI) *m/z*: $[M + Na]^+$ Calcd for $C_{20}H_{25}F_3O_9SNa$ 521.1064; Found 521.1106.

α_D^{25}= +68.4 (c 0.43, CH_2Cl_2)

F.2. General procedure for derivatization via Li/I exchange and electrophile trapping

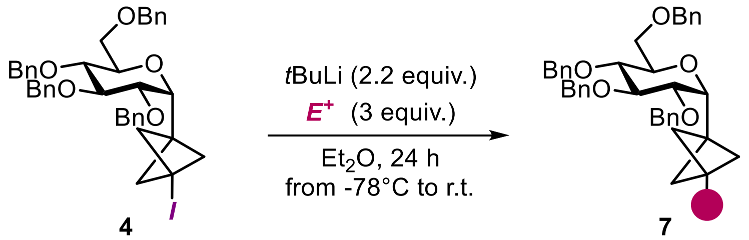

A 4 mL screw-cap vial with a PTFE/silicone septum and a magnetic stir bar was charged with 1-(2,3,4,6-tetra-*O*-benzyl-α-D-glucopyranosyl)-3-iodobicyclo[1.1.1]pentane **4d** (1.0 equiv.). The vial was sealed, evacuated under vacuum and back-filled with N_2 three times.and sealed with a PTFE/silicone septum. Anhydrous Et_2O (0.28 M) was added under N_2 atmosphere and the solution was cooled to −78°C (dry ice/acetone bath). Then *tert*-butyllithium (t-BuLi, 1.7 M in pentane, 2.2 equiv.) was added dropwise and the reaction mixture was stirred at −78°C for 2 h. The appropriate electrophile (3.0 equiv.) was then added dropwise at −78°C (if solid, the electrophile was added as a solution in anhydrous Et_2O). The reaction mixture was stirred for a additional 2 h at −78 °C and then allowed to warm to r.t.. The reaction was quenched with saturated NH_4Cl aqueous solution (2 mL) and the aqueous phase was extracted with CH_2Cl_2 (3x5 mL). The reunited organic phases were washed with H_2O (5 mL) and brine (5 mL), dried over anhydrous Na_2SO_4, filtered, and the solvent was removed by evaporation under reduced pressure. The crude product was purified by flash column chromatography on silica gel to afford the functionalized BCP *C*-glycosyl compounds in the stated yield.

Note: During lithiation/electrophile-trapping experiments with ethyl formate and benzophenone, we found the lithiated BCP intermediate to be prone to protonation, giving the corresponding dehalogenated side-product in 47 and 55% yield (determined by ^{1}H NMR spectroscopy). This also explains the good, although incomplete, deuterium incorporation observed for **7a** (84% yield, 73%D).

As expected, such lithiation/electrophile-trapping strategy is not compatible with per-acetylated derivatives: applying the same protocol to **4a** resulted in a complex reaction mixture. In such cases, access to **7a–c** would require an additional deprotection/protection sequence, thereby lengthening the synthetic route and inevitably reducing its overall efficiency.

1-(2,3,4,6-tetra-*O*-benzyl-α-D-glucopyranosyl)bicyclo[1.1.1]pentane-3-*d* 7a-*d₁*

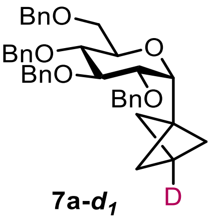

7a-*d*$_1$

Compound **7a-*d*$_1$** was synthesized following general procedure F.2. using 1-(2,3,4,6-tetra-*O*-benzyl-α-D-glucopyranosyl)-3-iodobicyclo[1.1.1]pentane **4d** (36 mg, 0.05 mmol), *t*-BuLi 1.7 M in pentane (65 µL, 0.11 mmol), MeOH-d_4 (8.2 µL, 0.15mmol), and anhydrous Et_2O (175 µL, 0.28 M). After work-up, the crude mixture was purified by flash column chromatography (gradient hexane:EtOAc from 90:10 to 80:20) to give **7a-*d*$_1$** as a yellow solid (25 mg, 84% yield, [73% D]).

^{1}H NMR (400 MHz, $CDCl_3$) δ 7.34 – 7.25 (m, 18H), 7.17 – 7.10 (m, 2H), 4.96 (d, *J* = 11.0 Hz, 1H), 4.85 – 4.78 (m, 2H), 4.69 – 4.56 (m, 3H), 4.52 – 4.44 (m, 2H), 3.99 (d, *J* = 6.3 Hz, 1H), 3.92 (t, *J* = 9.2 Hz, 1H), 3.85 (dt, *J* = 10.0, 3.1 Hz, 1H), 3.77 (dd, *J* = 9.6, 6.3 Hz, 1H), 3.68 – 3.60 (m, 2H), 3.53 (t, *J* = 9.4 Hz, 1H), 2.02 (d, *J* = 9.5 Hz, 3H), 1.94 (d, *J* = 9.5 Hz, 3H).

^{13}C NMR (101 MHz, $CDCl_3$, one aromatic *C*H is overlapped) δ 139.0, 138.6, 138.5, 138.3, 128.5, 128.5, 128.5, 128.2, 128.1, 128.0, 127.9, 127.8, 127.7, 127.7, 127.6, 82.3, 81.3, 78.4, 75.6, 75.2, 73.6, 73.4, 72.9, 72.5, 69.4, 52.8, 52.7, 45.5.

HRMS (ESI) *m*/*z*: $[M + Na]^+$ Calcd for $C_{39}H_{41}DO_5Na$ 614.2987; Found 614.2979.

α_D^{25} = +53.1 (c 0.48, CH_2Cl_2)

3-(2,3,4,6-tetra-*O*-benzyl-α-D-glucopyranosyl)bicyclo[1.1.1]pentane-1-carbaldehyde 7b

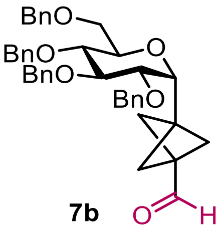

7b

Compound **7b** was synthesized following general procedure F.2. using 1-(2,3,4,6-tetra-*O*-benzyl-α-D-glucopyranosyl)-3-iodobicyclo[1.1.1]pentane **4d** (72 mg, 0.1 mmol), *t*-BuLi 1.7 M in pentane (130 µL, 0.22 mmol), ethyl formate (25 µL, 0.3 mmol), and anhydrous Et_2O (350 µL, 0.28 M). After work-up, the crude mixture was purified by flash column chromatography (gradient hexane:EtOAc from 90:10 to 80:20) to give **7b** as a yellow oil (25 mg, 27% yield).

^{1}H NMR (400 MHz, $CDCl_3$) δ 9.53 (s, 1H), 7.35 – 7.25 (m, 18H), 7.17 – 7.11 (m, 2H), 4.94 (d, *J* = 11.0 Hz, 1H), 4.81 (d, *J* = 11.1 Hz, 2H), 4.67 (d, *J* = 11.6 Hz, 1H), 4.64 – 4.55 (m, 2H), 4.52 – 4.45 (m, 2H), 4.03 (d, *J* = 6.0 Hz, 1H), 3.90 – 3.74 (m, 3H), 3.68 – 3.58 (m, 2H), 3.54 (dd, *J* = 9.9, 8.3 Hz, 1H), 2.23 (dd, *J* = 9.4, 1.8 Hz, 3H), 2.16 (dd, *J* = 9.4, 1.9 Hz, 3H).

^{13}C NMR (101 MHz, CDCl$_3$) δ 198.5, 138.7, 138.3, 138.2, 138.1, 128.6, 128.5, 128.5, 128.5, 128.1, 128.1, 128.0, 127.9, 127.9, 127.9, 127.8, 127.8, 82.3, 80.9, 78.1, 75.6, 75.2, 73.8, 73.7, 73.5, 72.1, 69.2, 52.6, 45.8, 41.1.

HRMS (ESI) *m/z*: $[M + H]^+$ Calcd for $C_{40}H_{43}O_6$ 619.3054; Found 619.3033.

α_D^{25}= +49.2 (c 0.42, CH_2Cl_2)

(3-(2,3,4,6-tetra-*O*-benzyl-α-D-glucopyranosyl)bicyclo[1.1.1]pentan-1-yl)diphenylmethanol 7c

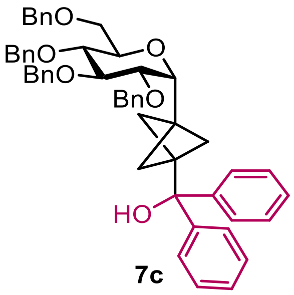

7c

Compound **7c** was synthesized following general procedure F.2. using 1-(2,3,4,6-tetra-*O*-benzyl-α-D-glucopyranosyl)-3-iodobicyclo[1.1.1]pentane **4d** (72 mg, 0.1 mmol), *t*-BuLi 1.7 M in pentane (130 µL, 0.22 mmol), benzophenone (49 mg, 0.3 mmol), and anhydrous Et_2O (350 µL, 0.28 M). After work-up, the crude mixture was purified by flash column chromatography (gradient hexane:EtOAc from 90:10 to 70:30) to give **7c** as a yellow solid (25 mg, 32% yield).

^{1}H NMR (400 MHz, CDCl$_3$) δ 7.45 (t, *J* = 7.4 Hz, 4H), 7.35 – 7.21 (m, 24H), 7.15– 7.08 (m, 2H), 4.94 (d, *J* = 11.0 Hz, 1H), 4.84 – 4.77 (m, 2H), 4.65 – 4.54 (m, 3H), 4.49 – 4.42 (m, 2H), 4.03 (d, *J* = 6.2 Hz, 1H), 3.86 (t, *J* = 9.2 Hz, 1H), 3.80 – 3.69 (m, 2H), 3.66 – 3.58 (m, 2H), 3.54 (t, *J* = 9.3 Hz, 1H), 2.03 (d, *J* = 9.5 Hz, 3H), 1.97 (d, *J* = 9.5 Hz, 3H).

^{13}C NMR (101 MHz, CDCl$_3$, three aromatic *C*H are overlapped) δ 144.9, 144.8, 138.9, 138.4, 138.4, 138.2, 128.5, 128.5, 128.5, 128.2, 128.1, 128.0, 128.0, 128.0, 127.8, 127.78, 127.8, 127.7, 127.0, 126.9, 126.9, 82.3, 80.9, 78.2, 76.7, 75.6, 75.2, 73.6, 73.4, 73.0, 72.3, 69.2, 51.5, 48.3, 40.4.

HRMS (ESI) *m/z*: $[M + Na]^+$ Calcd for $C_{52}H_{52}O_6Na$ 795.3656; Found 795.3611.

α_D^{25}= +37.7 (c 0.32, CH_2Cl_2)

G. MECHANISTIC INSIGHTS

Synthesis of 2,3,4,6-tetra-*O*-acetyl-α-D-glucosyl iodide 2a

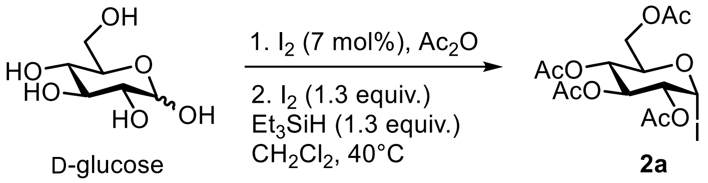

Compound **2a** was prepared following an already reported procedure.[29] To a round bottom flask equipped with a magnetic stir bar D-glucose (3.0 g, 16.8 mmol, 1 equiv.) was added. The flask was cooled down to 0°C, then Ac_2O (8.1 mL) and iodine (300 mg, 1.2 mmol, 7mol%) were added. The mixture was stirred at room temperature for 2 h, then diluted with CH_2Cl_2 (12 mL) and iodine (5.60 g, 21.9 mmol, 1.3 equiv.) and triethylsilane (3.5 mL, 21.9 mmol, 1.3 equiv.) were added. The mixture was stirred at 40 °C for 2 h then cooled to room temperature and diluted with CH_2Cl_2 (50 mL), then treated with saturated $Na_2S_2O_3$ aqueous solution

(20 mL). The layers were separated, and the aqueous layer was extracted with CH_2Cl_2 (2×40 mL). The combined organic layers were washed with brine, dried over anhydrous Na_2SO_4, filtered and concentrated in vacuo. Purification by column chromatography (eluent, Hex:Et_2O, 1:1) afforded **2a** (5.5 g, 71%) as a pale yellow solid. A second purification was required to obtain **2a** as a white solid that was suitable for electrochemical, photophysical characterization, and mechanistic investigation. The characterization data are in agreement with those previously reported.[29]

^{1}H NMR (400 MHz, $CDCl_3$) δ 6.99 (d, J = 4.3 Hz, 1H), 5.47 (t, J = 9.6 Hz, 1H), 5.18 (dd, J = 10.4, 9.3 Hz, 1H), 4.40 – 4.28 (m, 1H), 4.21 (dd, J = 9.9, 4.3 Hz, 1H), 4.12 (dd, J = 12.3, 1.7 Hz, 1H), 4.06 (dt, J = 10.5, 2.4 Hz, 1H), 2.10 (s, 3H), 2.09 (s, 3H), 2.06 (s, 3H), 2.03 (s, 3H).

G.1. UV-Vis absorption spectra

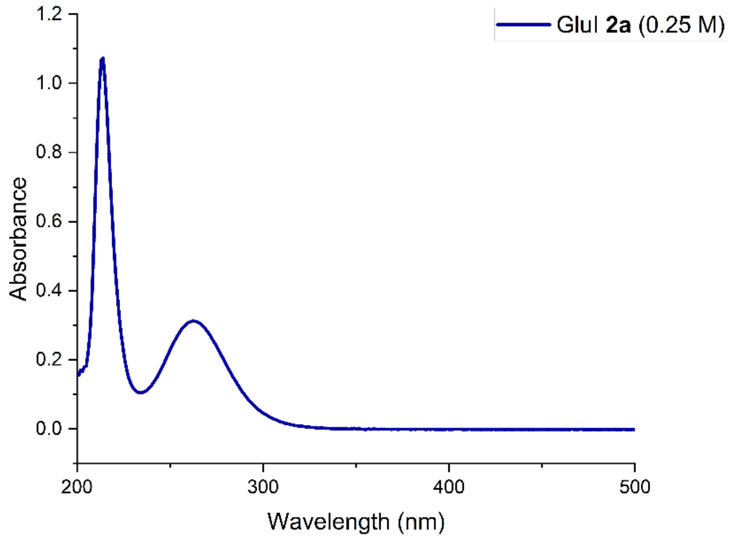

Figure S4. UV-Vis absorption spectra of 2,3,4,6-tetra-*O*-acetyl-α-D-glucopyranosyl iodide **2a** 0.25M solution in DCE:Et_2O, 1:1 solution. Recorded in quartz cuvettes, 1 mm path.

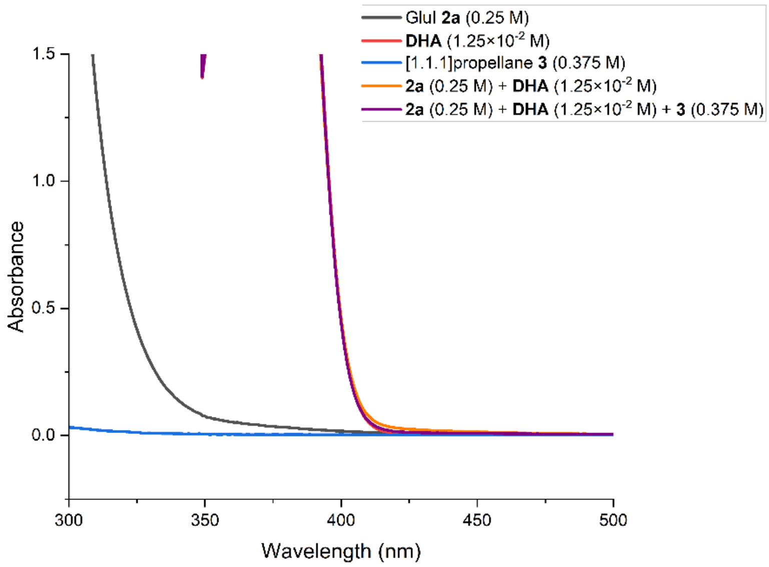

Figure S5. UV-Vis absorption spectra of glucopyranosyl iodide **2a** (black line), photocatalyst **DHA** (red line), [1.1.1]propellane **3** (blue line), the mixture of **2a** and **DHA** (orange line), and the reaction mixture (purple line). [**2a**] = 0.25 M; [**DHA**] = 1.25×10^{-2} M; [**3**] = 0.375 M in DCE:Et_2O, 1:1. Recorded in quartz cuvettes, 1 mm path.

Comment: To assess whether the reaction could be initiated by direct photolysis, we performed control experiments revealing that the process can indeed proceed in the absence of the photocatalyst, albeit with reduced efficiency (See Table S3). This observation is consistent with UV-Vis absorption studies of ***2a*** *at the reaction concentration (0.25M), which revealed weak, but not neglectable, absorption in the 350-450 nm range. We therefore believe that, under the optimized conditions, both irradiation of PC and direct excitation of* ***2a*** *contribute to radical initiation.*

G.2. Stern-Volmer quenching studies

Emission quenching studies were performed using a **DHA** solution (2.5×10^{-5} M in DCE:Et_2O, 1:1 mixture) without degassing. A 1 × 1 cm quarz cuvette was filled with 3 mL of the **DHA** solution. The emission was recorded (λexcitation = 390 nm) prior and after the addition of increasing amounts of a glucopyranosyl iodide **2a** stock solution (0.5 M in DCE:Et_2O, 1:1 mixture). The emission spectra revealed that **2a** is able to quench the photoexcited **DHA**, with the corresponding plot showing good linearity for the Stern−Volmer equation:

$$\frac{I_0}{I} = 1 + k_{SV}[Q]$$

Excited-state lifetime measurement for the **DHA** solution (2.5×10^{-5} M) in DCE:Et_2O, 1:1 mixture revealed a τ_0 value of 9.4 ns. From this, knowing the relationship $k_q = \frac{k_{SV}}{\tau_0}$, we obtained a k_q of 2.9×10^9 $M^{-1}s^{-1}$, which indicates that a quenching under diffusion-controlled rate can occur.

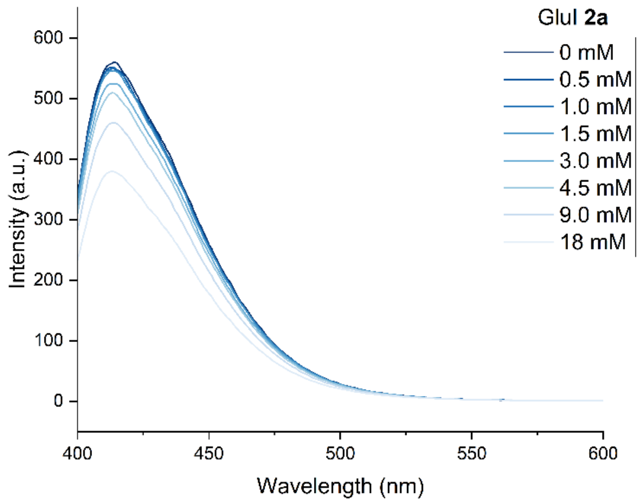

Figure S6. Emission spectra of a **DHA** solution (2.5 × 10^{-5} M in DCE:Et_2O, 1:1 mixture) upon increasing addition of glucopyranosyl iodide **2a** as quencher. Excitation wavelength λ = 390 nm.

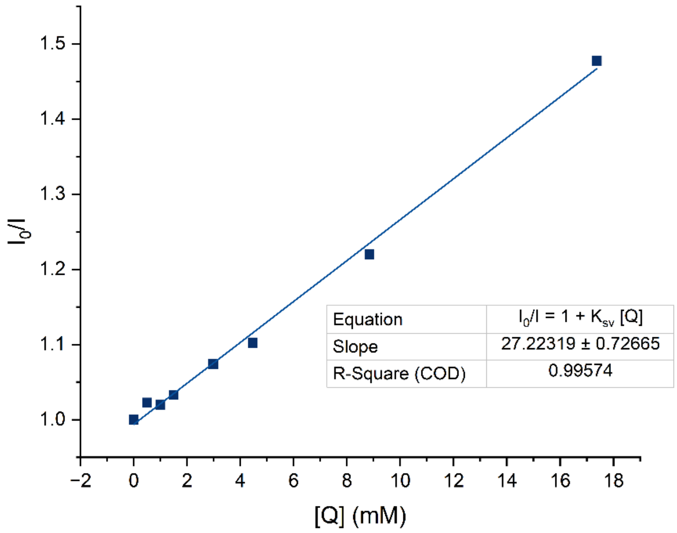

Figure S7. Stern-Volmer plot for the emission quenching of a **DHA** solution (2.5 × 10^{-5} M in DCE:Et_2O, 1:1 mixture) with glucopyranosyl iodide **2a** as quencher.

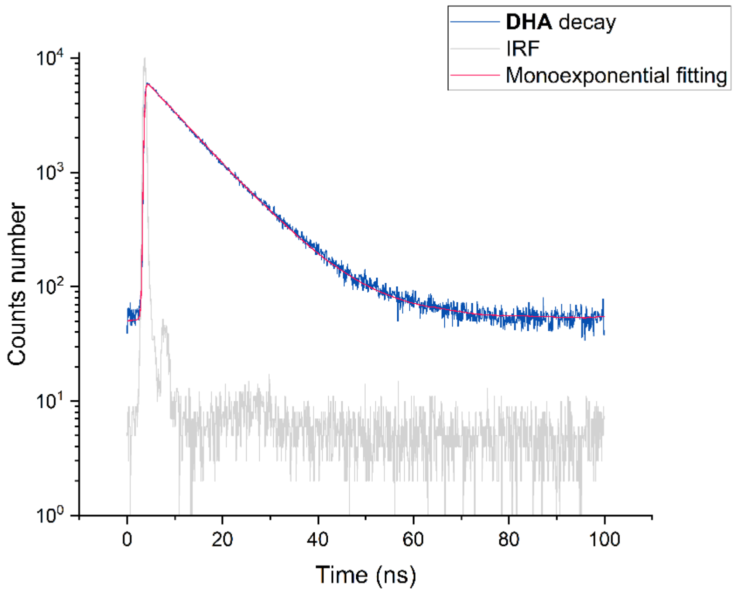

Figure S8. Emission decay of a **DHA** solution (2.5 × 10^{-5} M in DCE:Et_2O, 1:1 mixture); τ = 9.4 ns. Excitation: λ = 402.6 nm; Emission: λ = 430 nm.

G.3. Cyclic voltammetry measurements

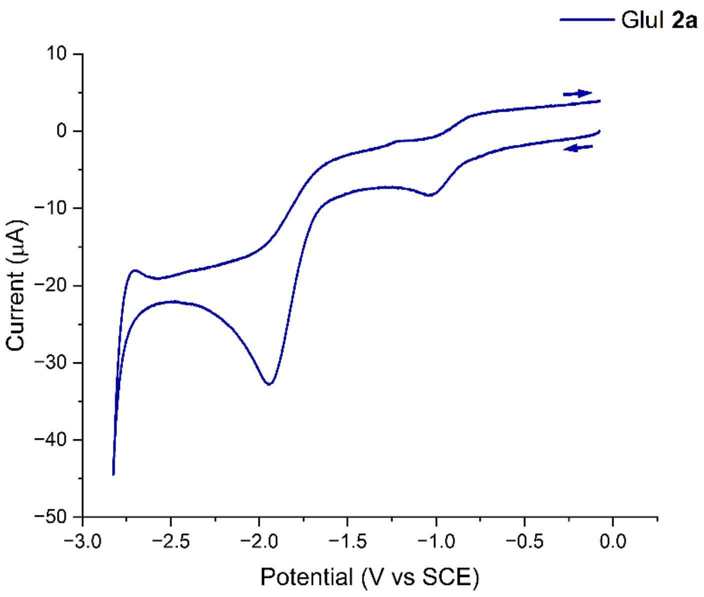

Figure S9. Cyclic voltammogram for compound 2,3,4,6-tetra-*O*-acetyl-α-D-glucopyranosyl iodide **2a** (1mM) in a Bu_4NPF_6 (0.1 M) solution in CH_3CN. The plot follows the IUPAC convention. Initial Potential: –0.08 V. Switching Potential: –2.83 V. Final potential: –0.08 V. Scan rate: 0.1 V/s. Glassy carbon working electrode; Ag/AgCl (3M, NaCl) reference electrode; Pt counter electrode. Irreversible reduction, $E_p^C = E_{red}$(**2a**/**2a**$^{\bullet-}$) = –1.94 V vs SCE; $E_{p1/2} = E_{red}$(**2a**/**2a**$^{\bullet-}$) = –1.80 V vs SCE. E_p^C refers to the cathodic peak potential, $E_{p1/2}$ refers to half-peak potential. The quasi-reversible wave at *E*= –0.89 V is ascribed to the reduction of residual O_2.[32]

G.4. TEMPO inhibition experiments

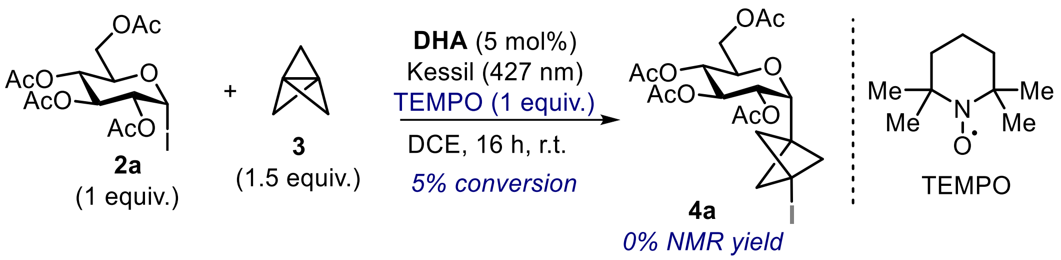

A 4 mL screw-cap vial with a PTFE/silicone septum and a magnetic stir bar was charged with TEMPO (15.6 mg, 0.1 mmol, 1 equiv.) and 2,3,4,6-tetra-*O*-acetyl-α-D-glucopyranosyl iodide **2a** (45.8 mg, 0.1 mmol, 1 equiv.), and **DHA** (1.6 mg, 5 μmol, 5 mol%). The vial was sealed, evacuated under vacuum and back-filled with Ar three times. Then, anhydrous DCE sparged with Ar (0.4 mL), and [1.1.1]propellane **3** solution (0.8 M in Et_2O, 180 μL, 0.15 mmol, 1.5 equiv.) were added in the order. The vial was sealed with parafilm, and the reaction mixture was stirred for 16 h at r.t. under the irradiation of a Kessil Lamp PR160L (λ_{max} 427 nm, 45W, 50% intensity) (see set-up in Figure S2, Section A). Then the crude mixture was analysed by ^{1}H NMR using trichloroethylene as internal standard to assess the conversion (5%) and yield (0%).

Comment: The reaction was completely inhibited when performed using TEMPO as radical scavenger. This observation indicates that the formation of ***4a*** *occurs via a radical pathway.*

G.5. Investigation on the compatibility of iodotrimethylsilane with [1.1.1]propellane 3

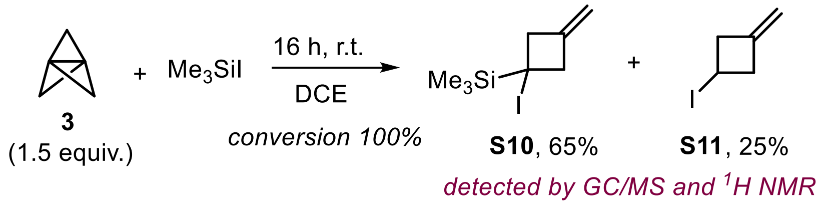

A 4 mL screw-cap vial with a PTFE/silicone septum and a magnetic stir bar was sealed, evacuated under vacuum and back-filled with N_2 three times. Then, anhydrous DCE sparged with N_2 (0.4 mL), iodotrimethylsilane (14 μL, 0.1 mmol, 1 equiv.), and [1.1.1]propellane **3** solution (0.83 M in Et_2O, 180 μL, 0.15 mmol, 1.5 equiv.) were added in the order. The vial was sealed with parafilm, and the reaction mixture was stirred for 3 h at r.t. under the irradiation of a Kessil Lamp PR160L (λ_{max} 427 nm, 45W, 50% intensity) (see set-up in Figure S2, Section A). Then the reaction crude was analysed by ^{1}H NMR using trichloroethylene as internal standard enabling to assess the formation of **S10** and **S11** in 65% and 25% yield, respectively. The spectroscopic data for **S11** are in agreement with a 1-substituted 3-methylenecyclobutane compound.[33] After evaporation of the solvent under reduced pressure, we were able to acquire a ^{1}H NMR spectrum of crude **S10**, while **S11** was no longer detectable, possibly due to its low boiling point. Compound **S10** was also detected by GC-MS analysis of the crude mixture.

^{1}H NMR (400 MHz, $CDCl_3$) δ 4.77 (quint., J = 2.7 Hz, 2H), 3.39 (d, J = 17.6 Hz, 2H), 3.25 (d, J = 17.6 Hz, 2H), 0.16 (s, J = 0.8 Hz, 9H).

GC-MS gradient 60°C for 1 min, then from 60°C to 290°C over 15 min, then 290°C for 3 min; τ = 4.29 min; *m*/*z* (%): 266.8 (0.07%), 139.1 (22%), 73.0 (100%).

Comment: This experiment reveals the high reactivity of Me_3SiI *to undergo electrophilic ring opening of propellane* ***3****. Indeed, precise fine-tuning of* Me_3SiI *stoichiometry is key to maximize the formation of glycosyl iodides while avoiding consumption of* ***3****. Compound* ***S11*** *likely arises from electrophilic addition of HI which is present in the batch of* Me_3SiI*. This experiment explains the re-quirement for in situ pre-activation of the substrate and the marked dependence of the reaction efficiency on* Me_3SiI *stoichiometry.*

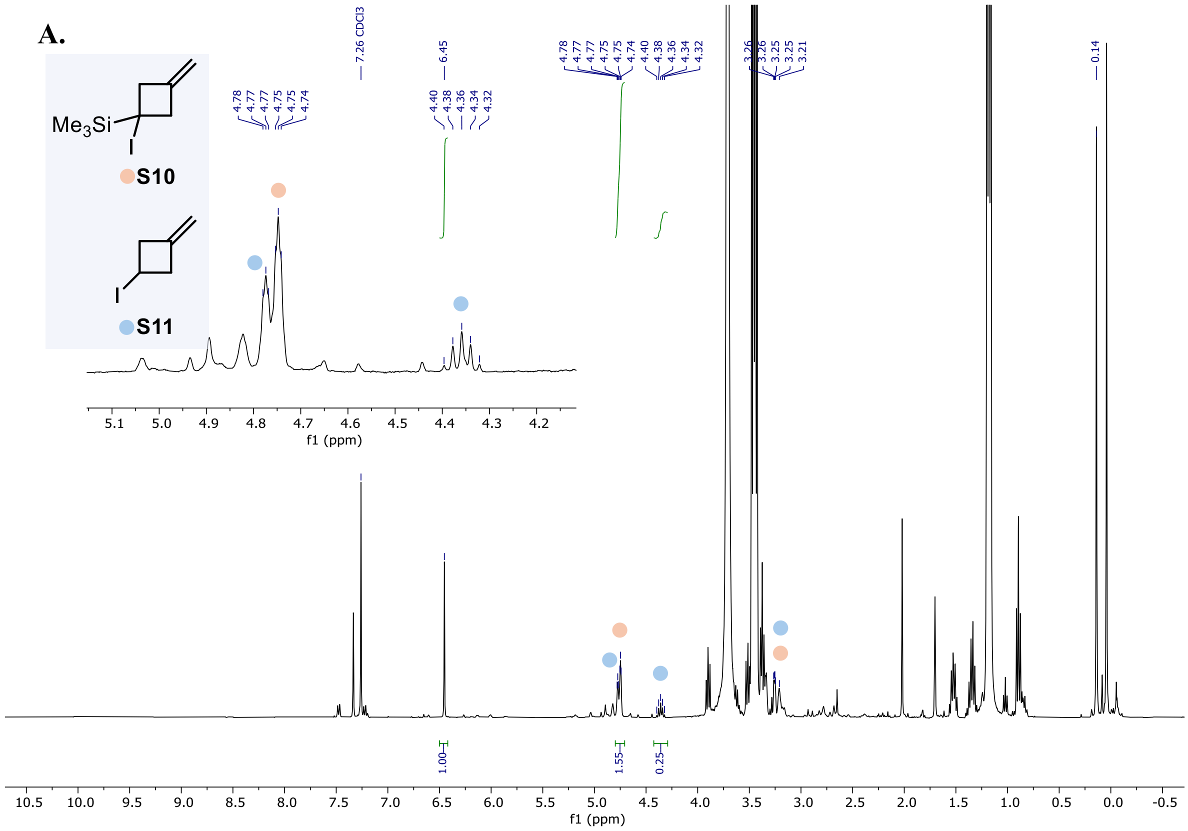

Figure S10. ^{1}H NMR analysis of the crude mixture from the reaction of [1.1.1]propellane **3** with iodotrimethylsilane, recorded in $CDCl_3$ as solvent using trichloroethylene (0.1 mmol) as internal standard. A) Identification of **S10** (65%) and **S11** (25%) (light orange and light blue circles, respectively.

B.

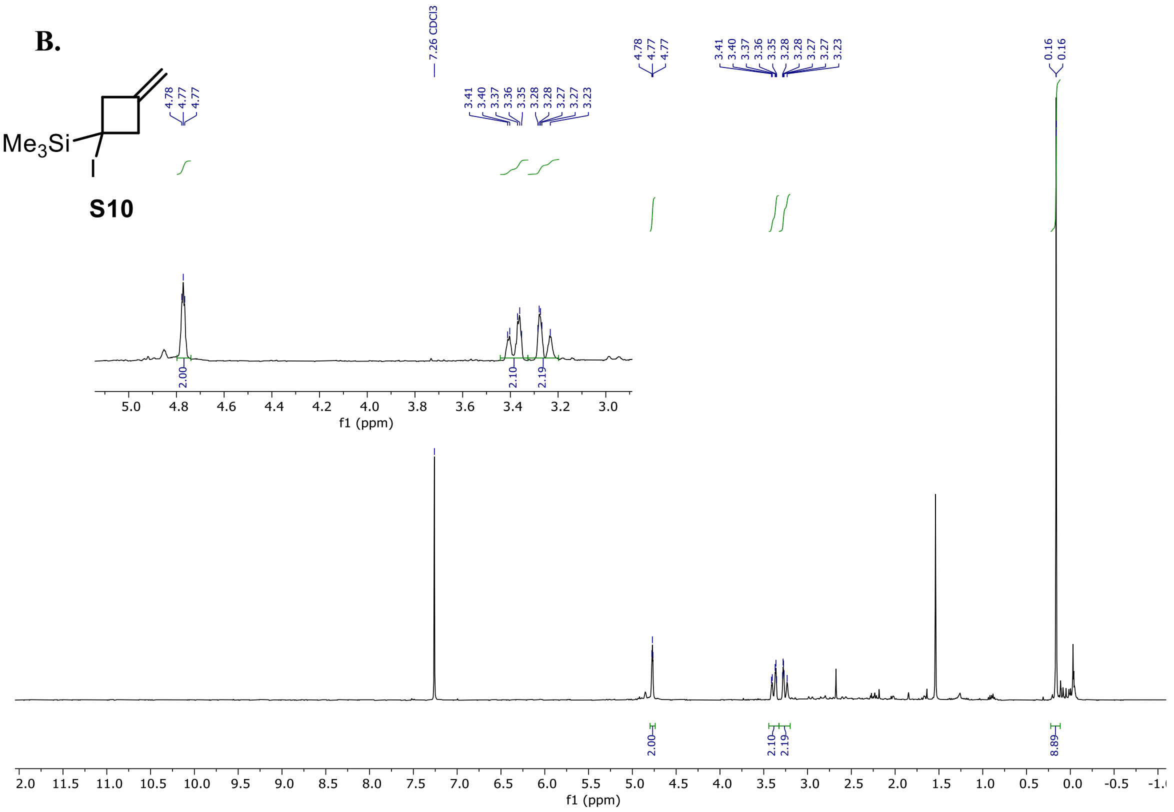

Figure S10 (Continued). ^{1}H NMR analysis of the crude mixture from the reaction of [1.1.1]propellane **3** with iodotrimethylsilane, recorded in $CDCl_3$ as solvent using trichloroethylene (0.1 mmol) as internal standard. A) Identification of **S10** (65%) and **S11** (25%) (light orange and light blue circles, respectively. B) ^{1}H NMR spectrum of crude **S10** after removing the solvent under reduced pressure.

G.6. Reaction profile

In agreement with previous studies by J. Gervay et al.,[34] we found **β-2a** to be in equilibrium with **α-2a**. More in detail, we found that when a solution of the model substrate **β-1a** (0.1 mmol) in DCE (0.4 mL) was treated with Me_3SiI (17 mL, 0.12 mmol) at 0°C, glycosyl iodide **β-2a** was formed selectively. To study the equilibration of **β-2a** towards **α-2a** under the optimized reaction conditions, the vial with the **β-2a** solution was irradiated using a Kessil lamp PR160L (λmax 427 nm, 45W, 50% intensity) (see set-up in Figure S2, Section A of the Supporting Information) and the reaction was sampled and monitored by ^{1}H NMR spectroscopy. We observed that **β-2a** is rapidly and quantitatively converted into the **α-2a** anomer within 30 minutes.

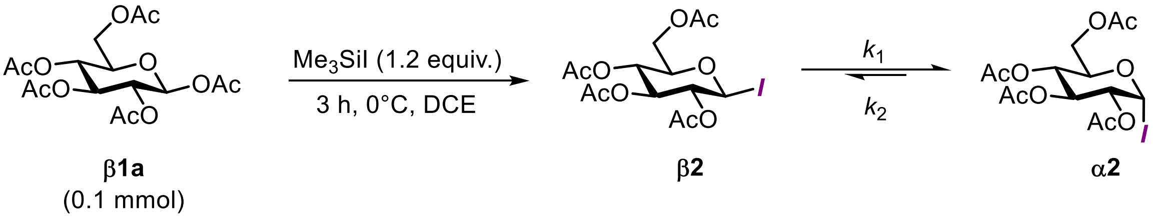

From these experimental data, knowing the following relationships:

$$Keq = \frac{[\alpha 2a]}{[\beta 2a]} \quad \text{and} \quad Keq = \frac{k_1}{k_2}$$

since at the equilibrium [**β-2a**] = 0, we have that $k_1 >> k_2$. Assuming that the equilibration reaction follows a first order rate law:

$$-\frac{d[\boldsymbol{\beta 2a}]}{dt} = k_1[\boldsymbol{\beta 2a}]$$

$$\ln[\boldsymbol{\beta 2a}] = -k_1 t + \ln[\boldsymbol{\beta 2a}]_0$$

Thus, by plotting the values of [**β-2a**] as a function of time, we obtain $k_1 = 1.9 \times 10^{-3}$ s^{-1}.

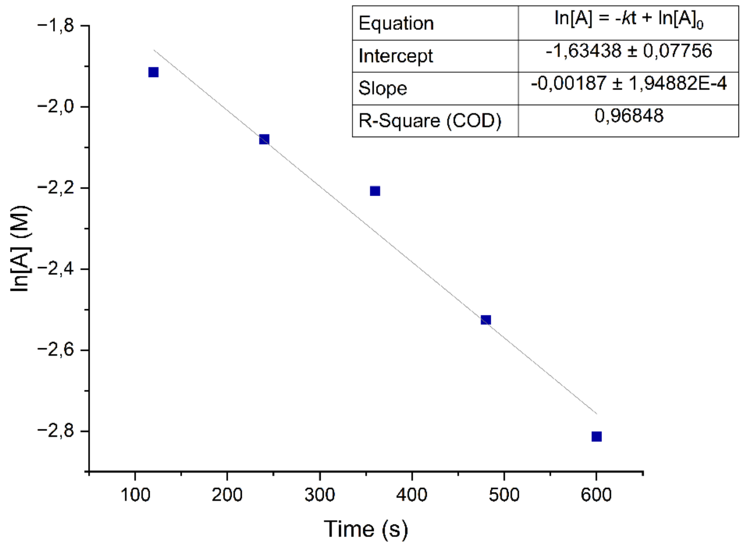

Figure S11. Linear fit for the logarithmic plot of the concentration of **β-2a** over time.

Due to this fast equilibrium ($t_{1/2}$ = 4 min), we were unable to individually study the reactivity of **β-2a** and **α-2a** under the optimized photocatalytic conditions. Nevertheless, we hypothesize that XAT abstraction can occur both from the α- and β-anomer. Previous studies from Giese et al. showed that configurationally stable α- and β-glycosyl chlorides reacts with comparable rates in Sn mediated XAT reactions.[35]

Similar considerations can be done for what concern the SET reduction of **2a**. Again, rapid equilibration of **β-2a** to **α-2a** prevented us from comparing the electrochemical properties of the two anomers and their ability to quench the excited state DHA*. From a theoretical point of view the thermodynamically less stable **β-2a** might be expected to display enhanced reactivity. On the other hand, SET and the subsequent cleavage of the C—I bond to generate the corresponding glycosyl radical could be more favorable for **α-2a**, owing to a more pronounced kinetic anomeric effect arising from better overlap between the *p* orbital of the endocyclic oxygen atom and the σ* orbital of the C—I bond. Overall, the relative reactivity of **β-2a** and **α-2a** is likely dictated by these two factors, which may either compensate each other or one may predominate.

The reaction profile was studied as follows: a reaction was performed according to general procedure D.1. using 1,2,3,4,6-penta-*O*-acetyl-β-D-glucopyranose **1a** (39 mg, 0.1 mmol), Me_3SiI (17 µL, 0.12 mmol), [1.1.1]propellane **3** solution (0.83 M in Et_2O, 180 µL, 0.15 mmol), **DHA** (1.6 mg, 5 µmol), and DCE (0.4 mL). Reaction time: 3 h then o.n.. During irradiation, the reaction progress was monitored by withdrawing 40 µL

aliquots via syringe, which were analyzed by ^{1}H NMR spectroscopy. Diagnostic peaks for α-**2a** and β-**2a** at δ 6.98, 5.75 ppm, respectively. Diagnostic peaks for **4** are doublets at δ 2.49, 2.40 ppm.

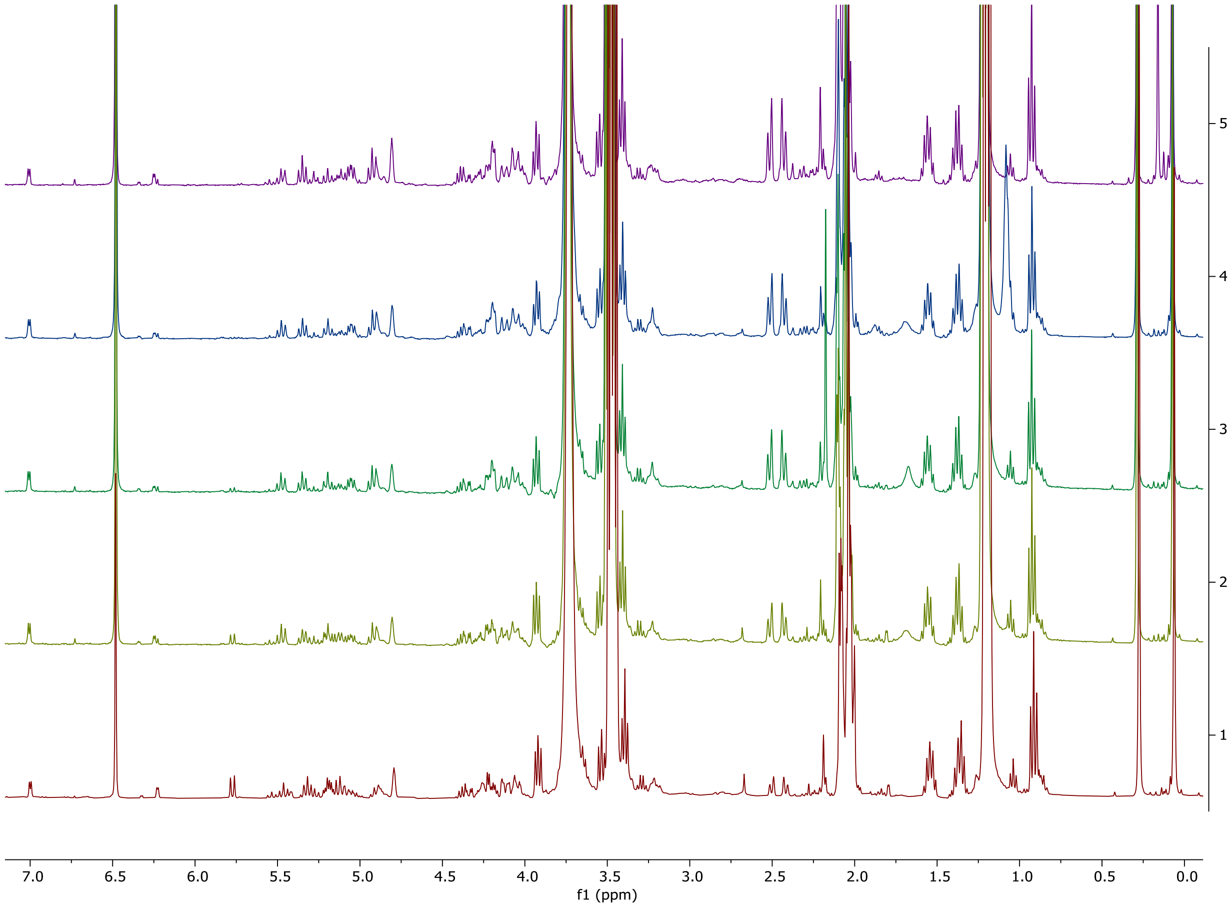

Figure S12. Monitoring of the model reaction by ^{1}H NMR analysis of the crude reaction mixture. Aliquots were withdrawn during irradiation at the following time points (bottom to top): 15, 35, 80, 180, 960 min.

The same procedure was repeated to monitor the profile of a reaction in the absence of the photocatalyst **DHA** (see Figure S13B below).

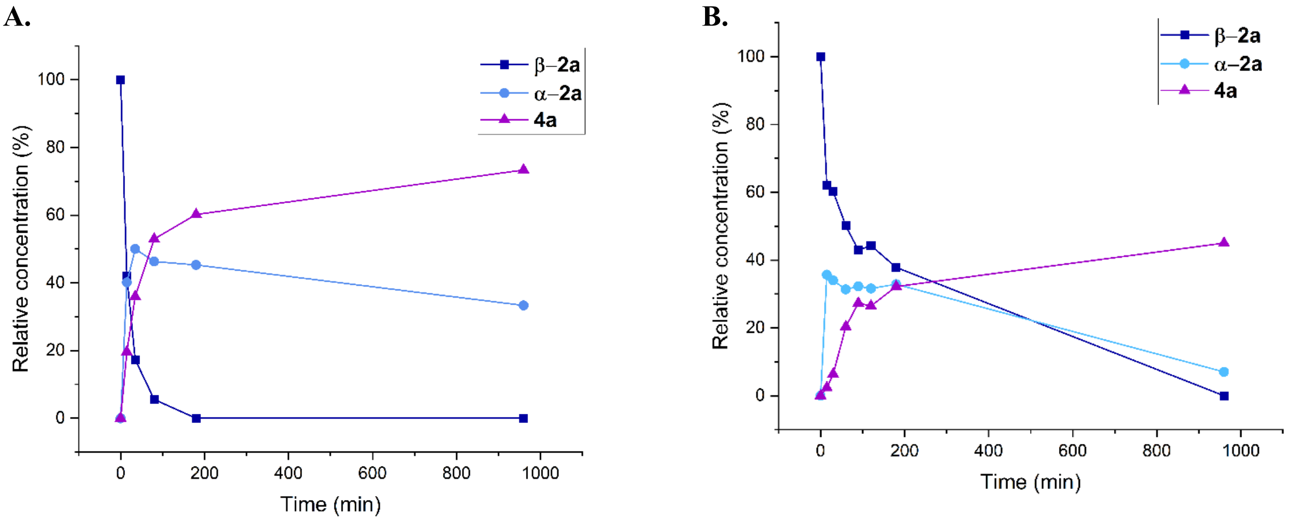

Figure S13. Monitoring of the A) model reaction and B) a reaction in the absence of **DHA** by ^{1}H NMR analysis of the crude reaction mixture.

I. REFERENCES

1. a) Aranzaes, J. R.; Daniel, M.-C.; Astruc, D. *Can. J. Chem.* **2006**, *84*, 288-299; b) Pavlishchuk, V. V.; Addison, A. W. *Inorganica Chim. Acta* **2000**, *298*, 97-102.
2. Wang, S.; Force, G.; Carpentier, J.-F.; Sarazin, Y.; Bour, C.; Gandon, V.; Lebœuf, D. *Org. Lett.* **2021**, *23*, 2565-2570.
3. Vaswani, R. G.; Chamberlin, A. R. *J. Org. Chem.* **2008**, *73*, 1661–1681.
4. Rossolini, T.; Leitch, J. A.; Grainger, R.; Dixon, D. J. *Org. Lett.* **2018**, *20*, 6794–6798.
5. Stark, D. G.; O'Riordan, T. J. C.; Smith, A. D. *Org. Lett.* **2014**, *16*, 6496–6499.
6. Pickford, H. D.; Nugent, J.; Owen, B.; Mousseau, J. J.; Smith, R. C.; Anderson, E. A. *J. Am. Chem. Soc.* **2021**, *143*, 9729–9736.
7. Ramirez, N. P.; Lana-Villarreal, T.; Gonzalez-Gomez, J. C. *Eur. J. Org. Chem.* **2020**, *2020*, 1539−1550.
8. Rodríguez, R. I.; Paut, J.; Armellin, G.; Visentini, S.; Cormier, G.; Droghetti, F.; Natali, M.; Bortolus, M.; Pelosi, G.; Dell'Amico, L. *Nat. Commun.* **2026** DOI: 10.1038/s41467-026-69464-3.
9. a) Fischer, E.; Fischer, H. *Ber. Dtsch. Chem. Ges.* **1910**, *43*, 2521−2536; b) Hudson, C. S.; Kunz, A. *J. Am. Chem. Soc.* **1925**, *47*, 2052–2055.
10. Michihata, N.; Kaneko, Y.; Kasai, Y.; Tanigawa, K.; Hirokane, T.; Higasa, S.; Yamada, H. *J. Org. Chem.* **2013**, *78*, 4319−4328.
11. Kunz, H.; Harreus, A. *Liebigs Ann. Chem.* **1982**, *1982*, 41−48.

12. Gulbe, K.; Luginina, J.; Jansons, E.; Kinens, A.; Turks, M. Beilstein *J. Org. Chem.* **2021**, *17*, 964–976.
13. Matwiejuk, M.; Thiem, J. *Eur. J. Org. Chem.* **2011**, *2011*, 5860–5878.
14. Cachatra, V.; Oliveira, M. C.; Lopez, O.; Fernandez-Bolaños, J. G.; Rauter, A. P. *Med. Chem. (Sharjah, United Arab Emirates)* **2023**, *19*, 263-275.
15. Uchiyama, T.; Shishikura, K.; Ogawa, K.; Ohshima, Y.; Miyairi, S. *Tetrahedron Lett.* **2016**, *57*, 5294–5296.
16. Yeh; C.-H.; Chang, Y.-J.; Lina, T.-J.; Wang, C.-C. *J. Am. Chem. Soc.* **2023**, *145*, 9003–9010.
17. Matassini, C.; Mirabella, S.; Goti, A.; Cardona, F. *Eur. J. Org. Chem.* **2012**, *2012*, 3920–3924.
18. Li, Y.; Wang, Z.; Li, L.; Tian, X.; Shao, F.; Li, C. *Angew. Chem. Int. Ed.* **2022**, *61*, e202110391.
19. Gelas, J.; Horton, D. *Carbohydr. Res.* **1978**, *67*, 371–387.
20. Deng, L.; Tsybina, P.; Gregg, K. J.; Mosi, R.; Zandberg, W. F.; Boraston, A. B.; Vocadlo, D. J. *Bioorg. Med. Chem.* **2013**, *21*, 4839–4845.
21. Doyle, L. M.; O'Sullivan, S.; Di Salvo, C.; McKinney, M.; McArdle, P.; Murphy, P. V. *Org. Lett.* **2017**, *19*, 5802–5805.
22. Szennyes, E.; Bokor, É.; Batta, G.; Docsa, T.; Gergely, P.; Somsák, L. *RSC Adv.* **2016**, *6*, 94787-94794.
23. Liao, W.-J.; Lin, S.-Y.; Kuo, Y.-S.; Liang, C.-F. *Org. Lett.* **2022**, *24*, 4207–4211.
24. Beahm, B. J.; Dehnert, K. W.; Derr, N. L.; Kuhn, J.; Eberhart, J. K.; Spillmann, D; Amacher, S. L.; Bertozzi, C. R., *Angew. Chem. Int. Ed.* **2014**, *53*, 3347–3352.
25. Pérez Figueroa, I.; Horváth, F.; Dékány, G.; Ágoston, K.; Ágoston, Á.; Bajza, I.; Boutet, J.; Hederos, M.; Kovács-Pénzes, P.; Kröger, L.; Röhrig, C.; Schroven, A.; Vrasidas, I.; Risinger, C. PRODUCTION OF 6'-*O*-SIALYLLACTOSE AND INTERMEDIATES, **2011**, WO2011100979.
26. Dey, S.; Giri, D.; Nandy, A.; Sau, A. *Green Chem.* **2025**, *27*, 4995–5000.
27. Wu, Z.; Cao, A.; Ding, W.; Zhu, T.; Shen, P. J. *Carbohydr. Chem.* **2016**, *35*, 355-366.
28. Mukhopadhyay, B.; Kartha, K. P. R:; Russella, D. A.; Field, R. A. *J. Org. Chem.* **2004**, *69*, 7758–7760.
29. Nugent, J.; Arroniz, C.; Shire, B. R.; Sterling, A. J.; Pickford, H. D.; Wong, M. L. J.; Mansfield, S. J.; Caputo, D. F. J.; Owen, B.; Mousseau, J. J.; Duarte, F.; Anderson, E. A. *ACS Catal.* **2019**, *9*, 9568−9574.
30. Liu, J.; Purushothaman, R.; Hinrichs, F.; Surke, M.; Warratz, S.; Ackermann, L. *J. Am. Chem. Soc.* **2025**, *147*, 34813–34822.
31. Murakami, T.; Sato, Y.; Shibakami, M. *Carbohydr. Res.* **2008**, *343*, 1297–1308.
32. a) Vasudevan, D.; Wendt, H. *J. Electroanal. Chem.* **1995**, *192*, 69-74; b) Wang, S.; Yang, J.; Li, D.; Yang, J. *Eur. J. Org. Chem.* **2021**, *2021*, e202101178.
33. Wiberg, K. B.; Walker, F. H. *J. Am. Chem. Soc.* **1982**, *104*, 5239-5240.
34. Gervay, J.; Nguyen, T. N.; Hadd, M. J., *Carbohydr. Res.* **1997**, *300*, 119-125.

35. Giese, B.; Dupuis, J., *Tetrahedron Lett.* **1984**, *25*, 1349-1352.

J. NMR SPECTRA

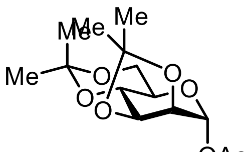

1h

^{1}H NMR

400 MHz, $CDCl_3$

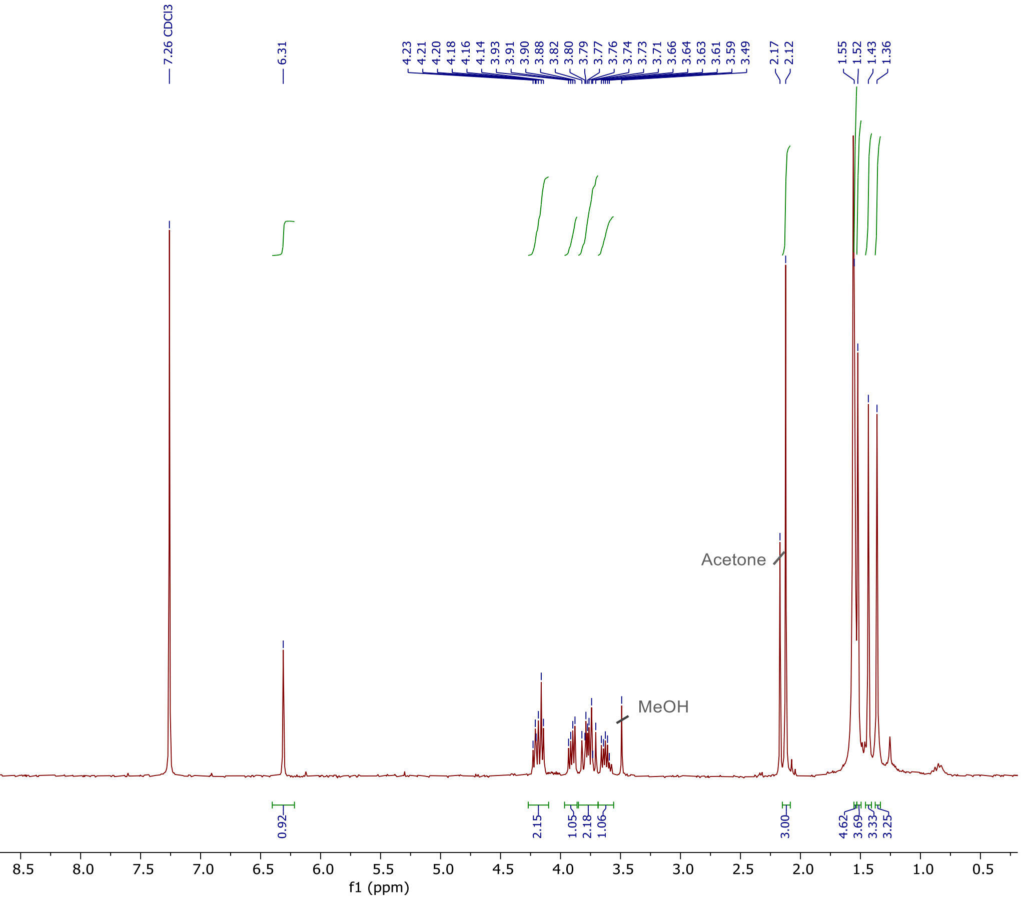

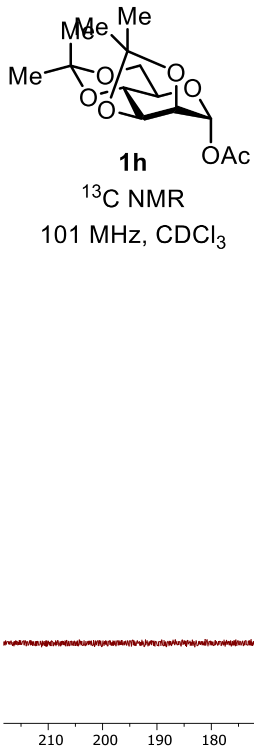

1h

^{13}C NMR

101 MHz, $CDCl_3$

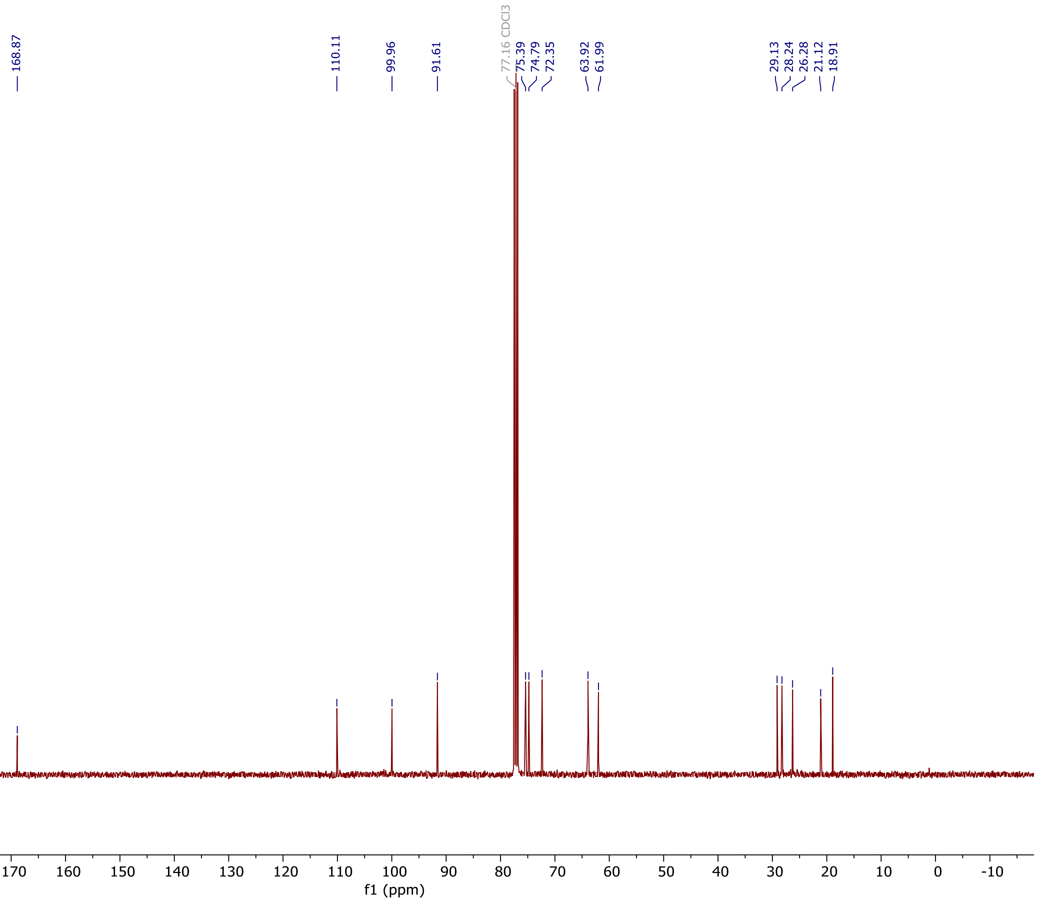

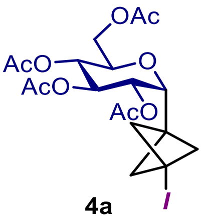

4a

^{1}H NMR

400 MHz, $CDCl_3$

α:β, 6:4

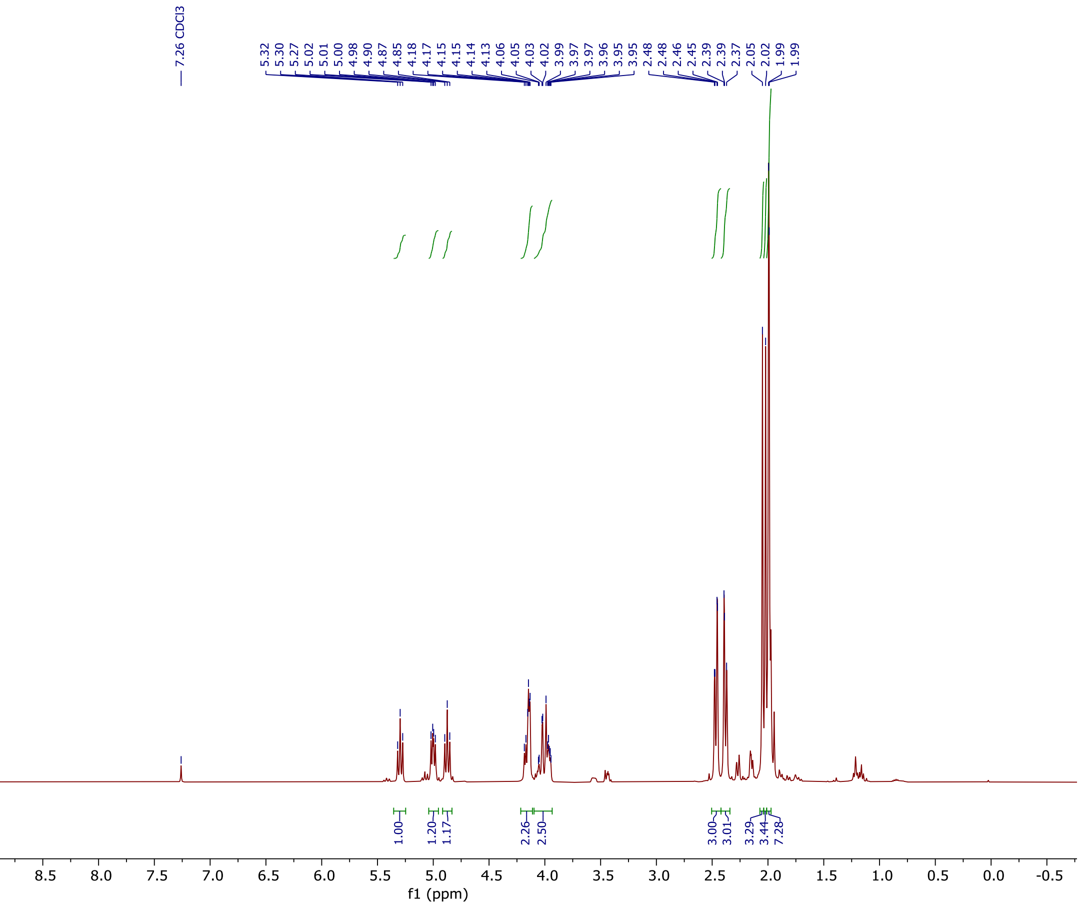

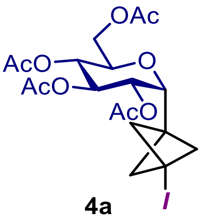

4a

^{13}C NMR

101 MHz, $CDCl_3$

α:β, 6:4

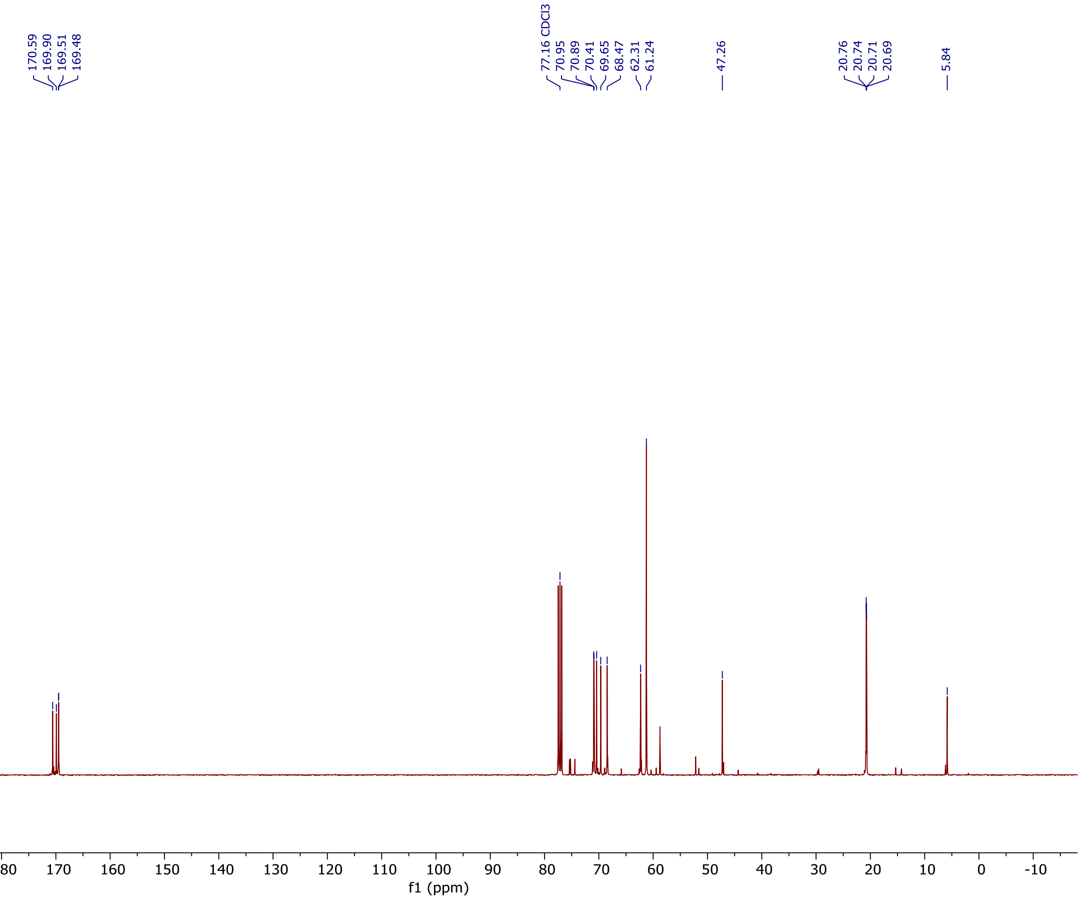

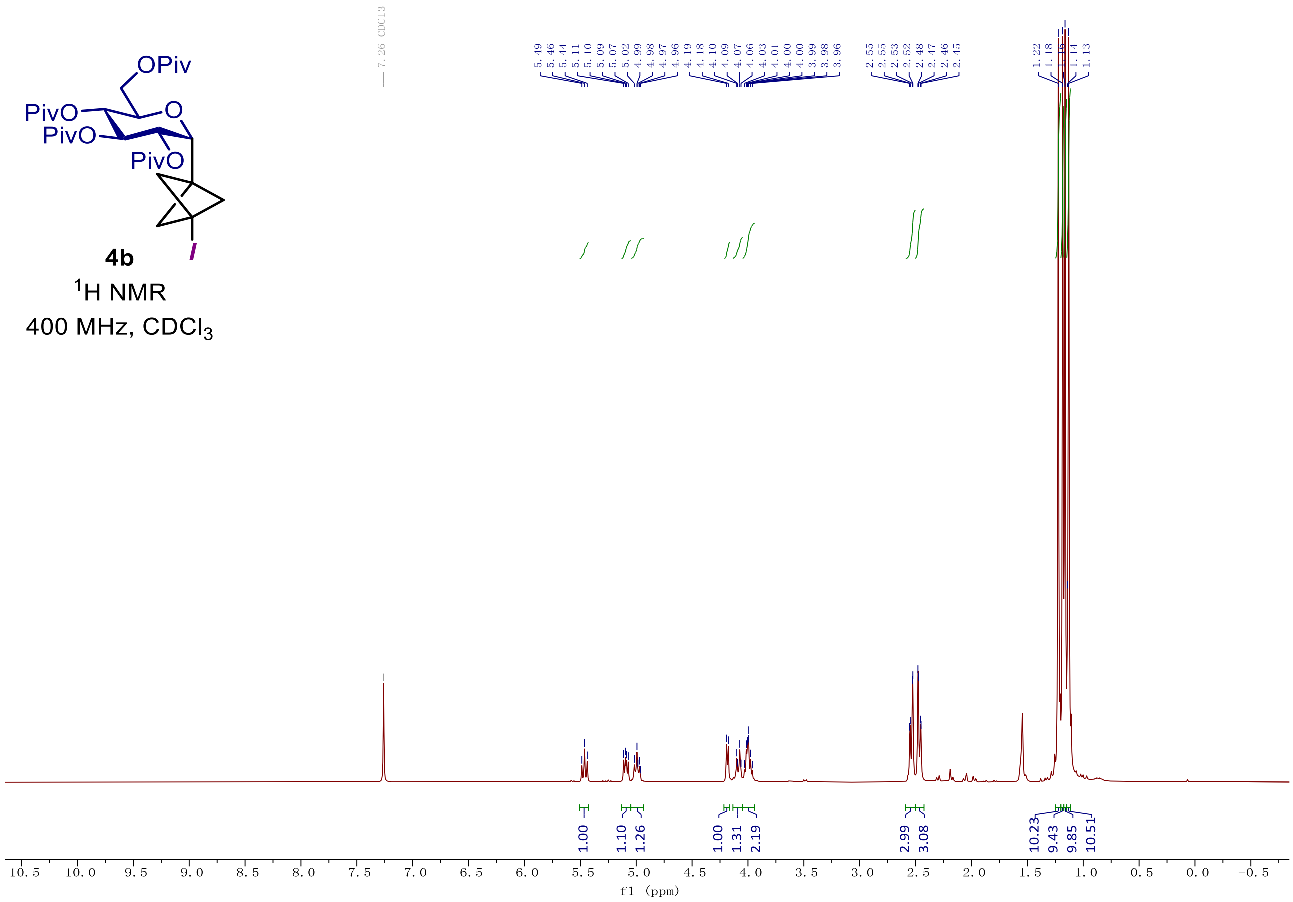
4b
^{1}H NMR
400 MHz, CDCl$_3$
f1 (ppm)

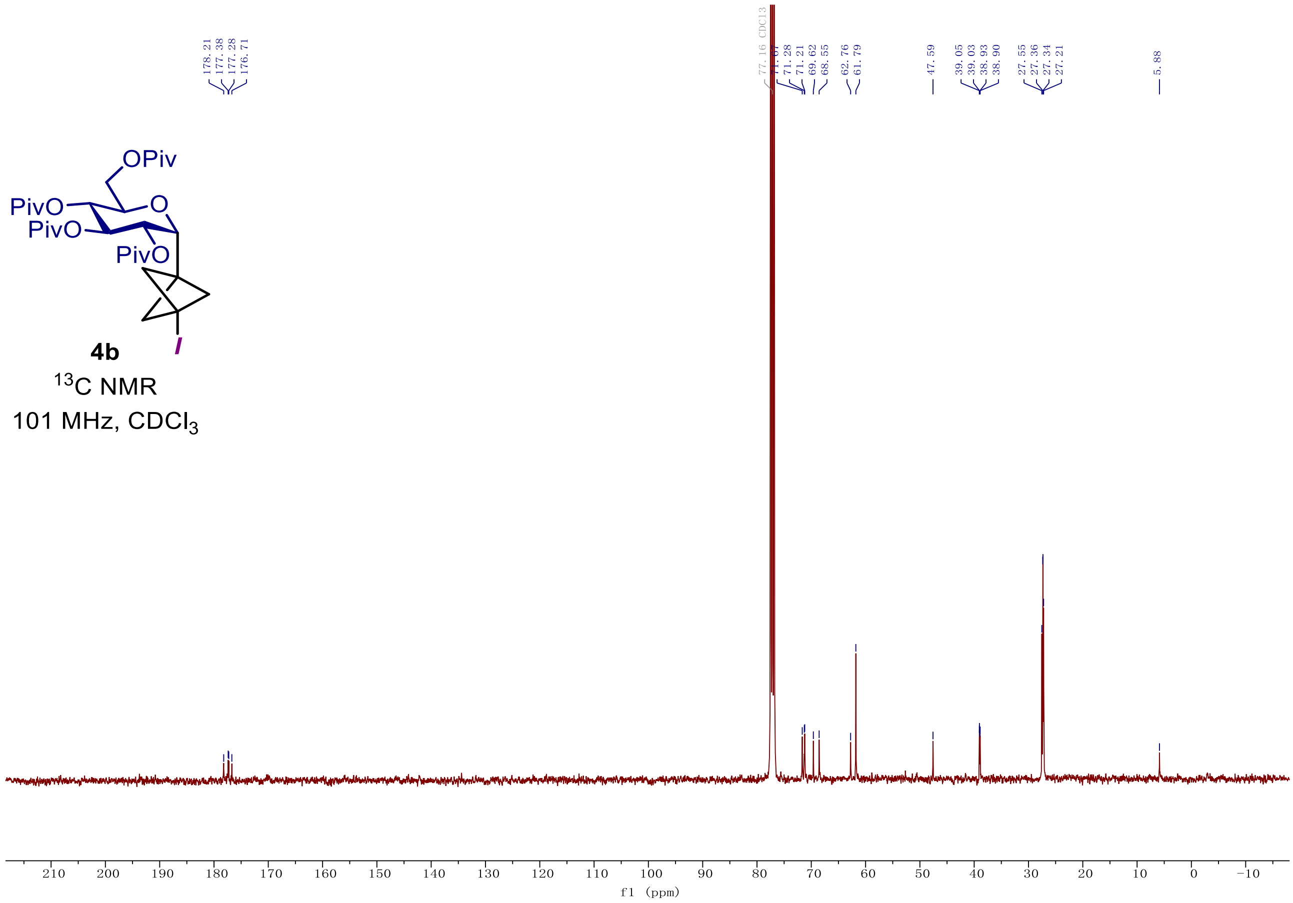
4b
^{13}C NMR
101 MHz, CDCl$_3$
f1 (ppm)

4d

^{1}H NMR

400 MHz, $CDCl_3$

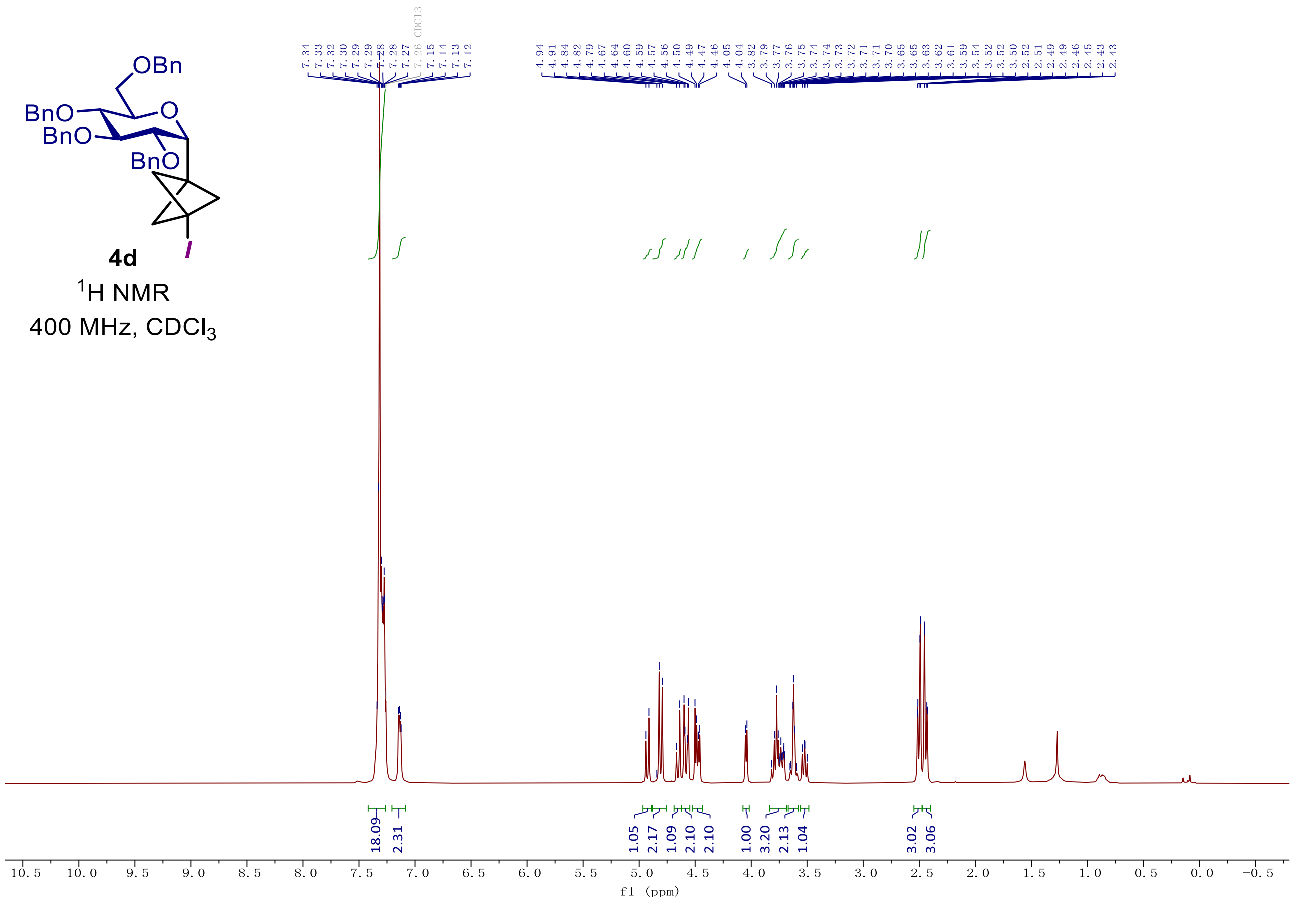

4d

^{13}C NMR

101 MHz, $CDCl_3$

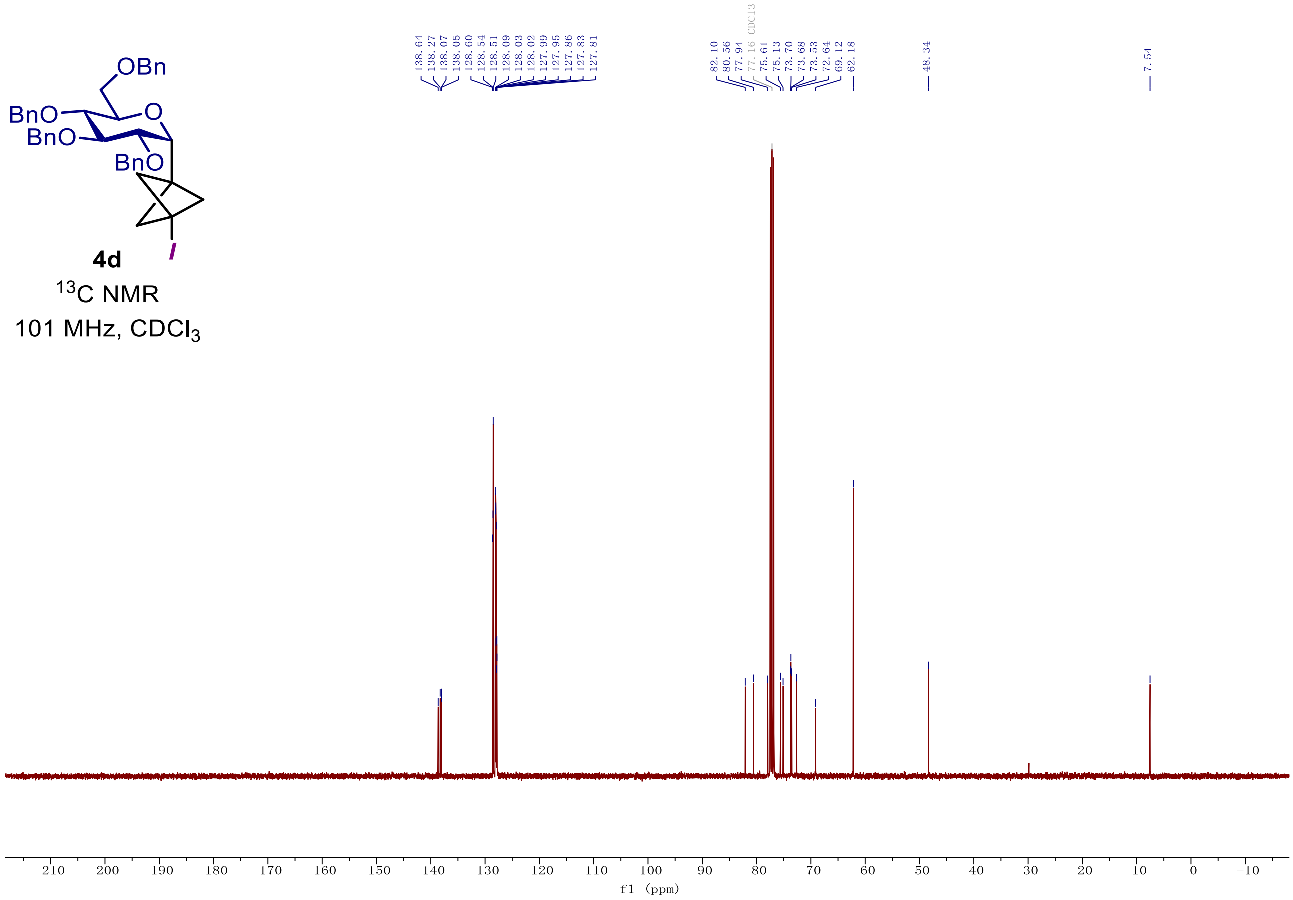

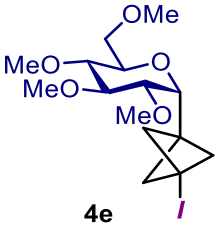

4e

^{1}H NMR

400 MHz, $CDCl_3$

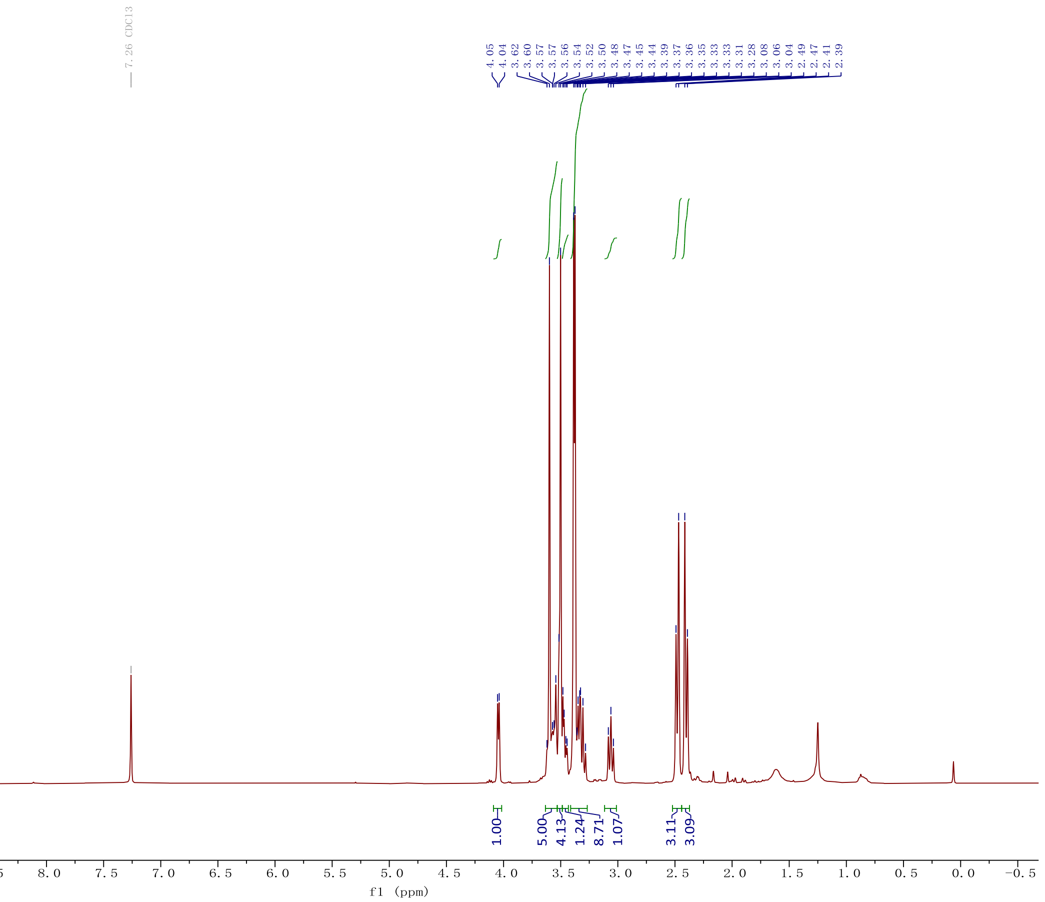

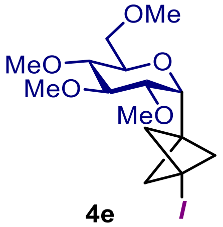

4e

^{13}C NMR

101 MHz, $CDCl_3$

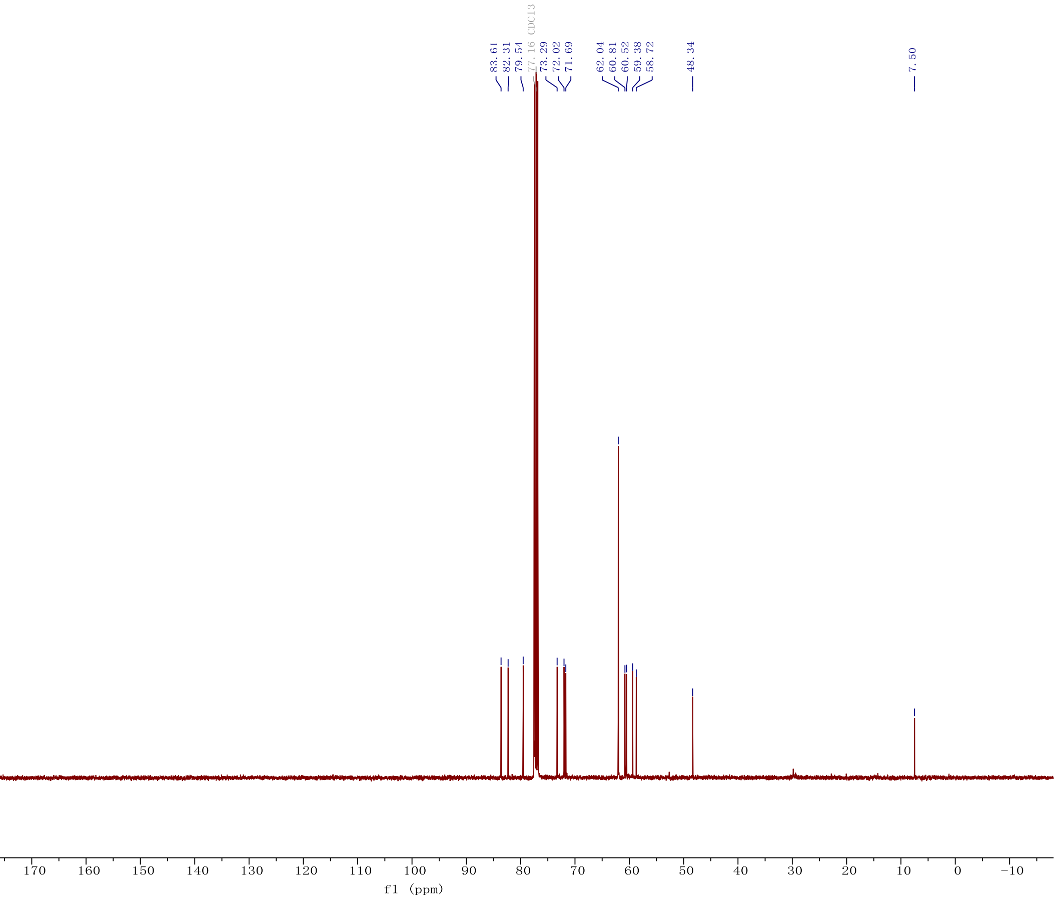

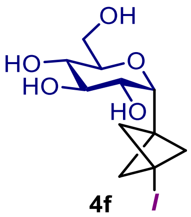

4f

^{1}H NMR

400 MHz, CD_3OD

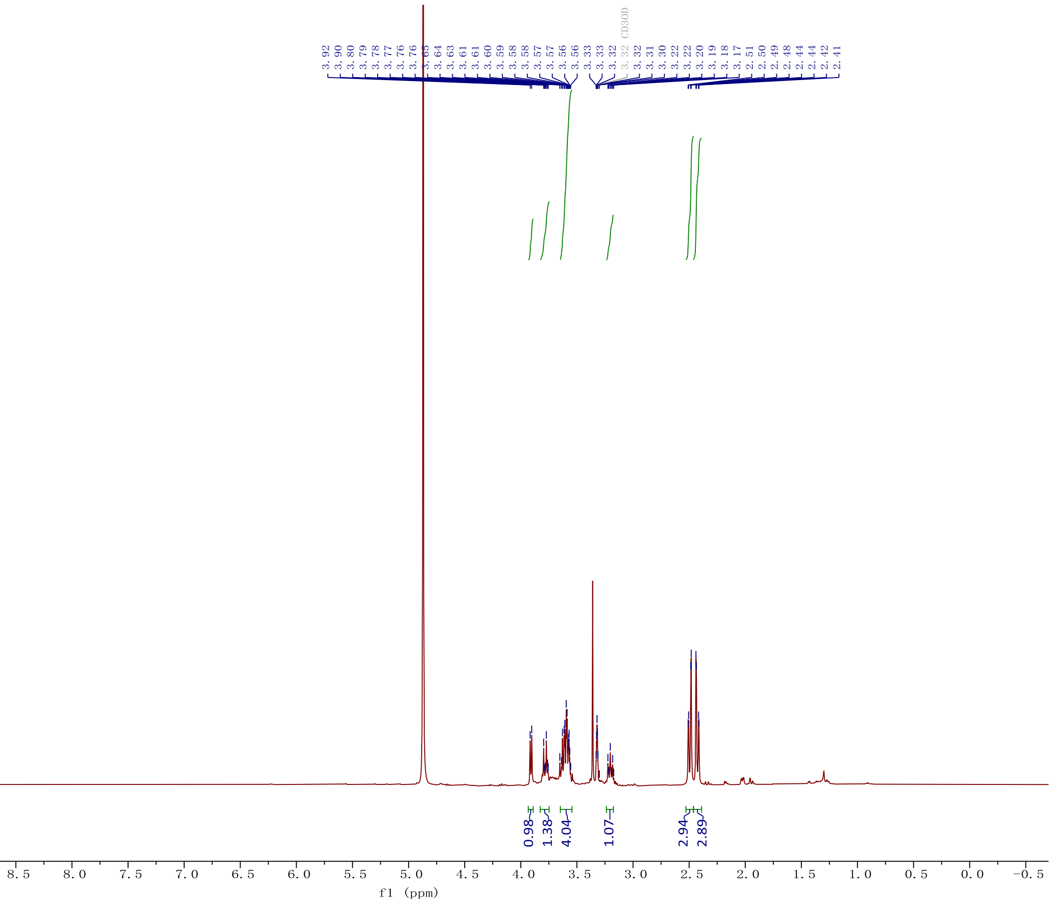

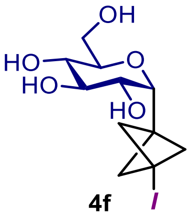

4f

^{13}C NMR

101 MHz, CD_3OD

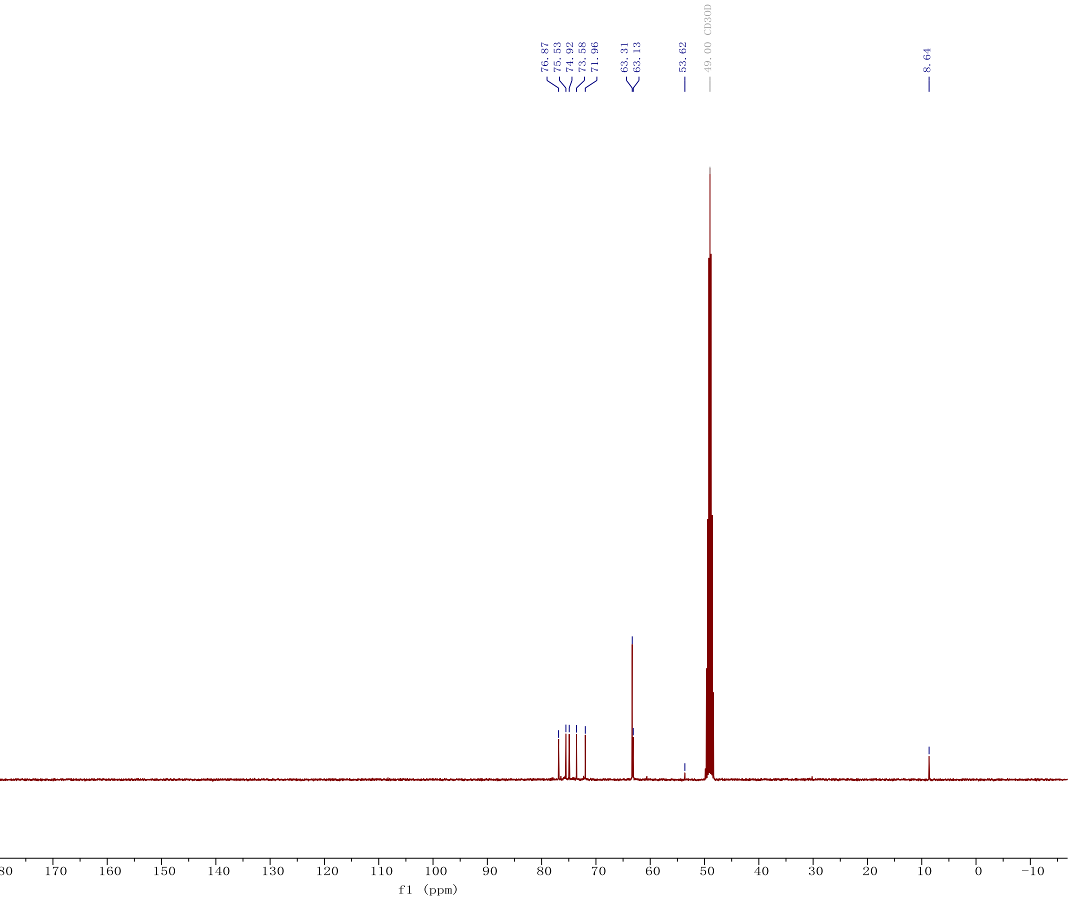

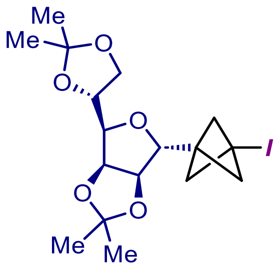

4g

^{1}H NMR

400 MHz, $CDCl_3$

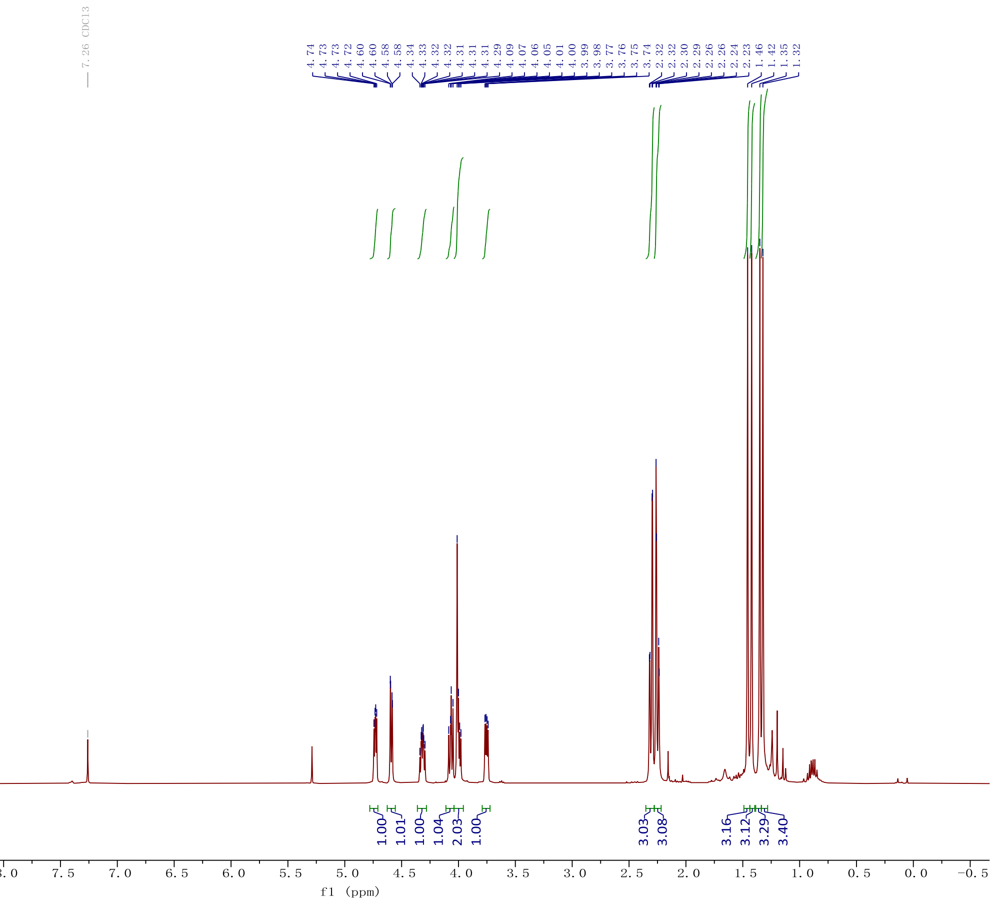

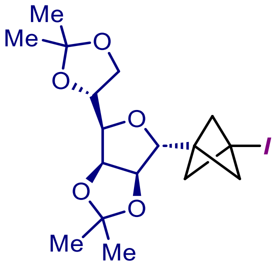

4g

^{13}C NMR

101 MHz, $CDCl_3$

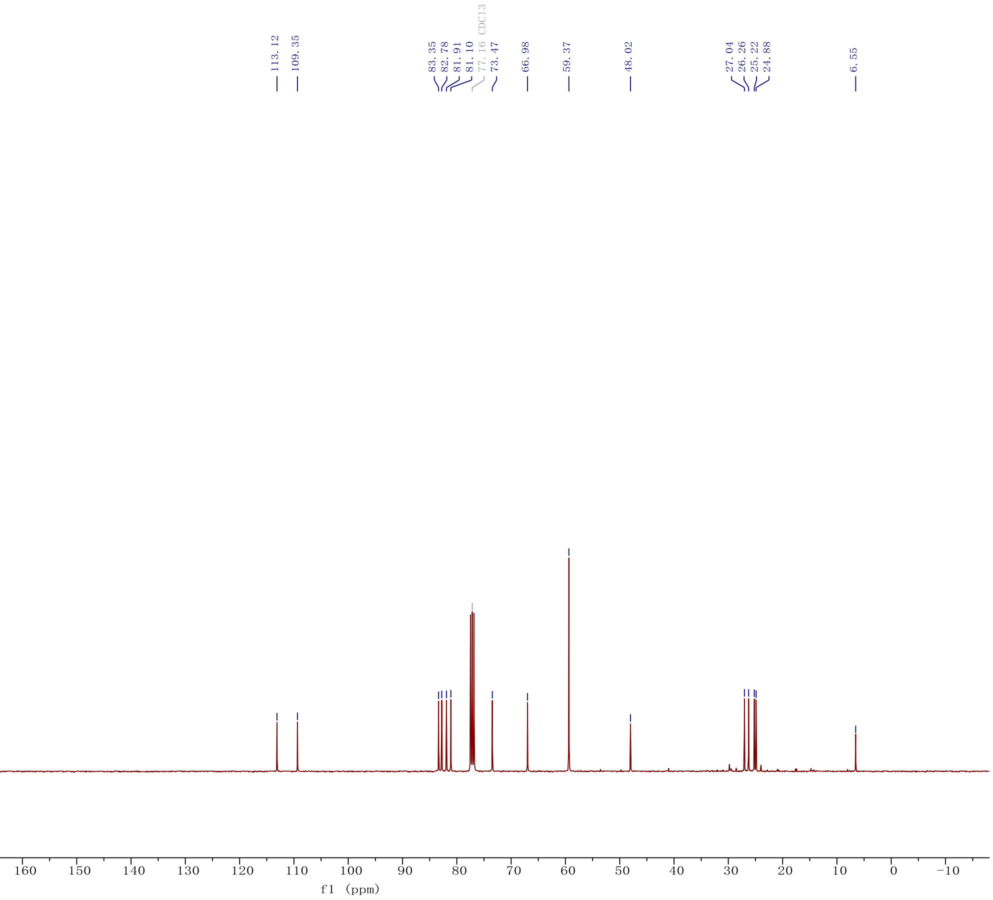

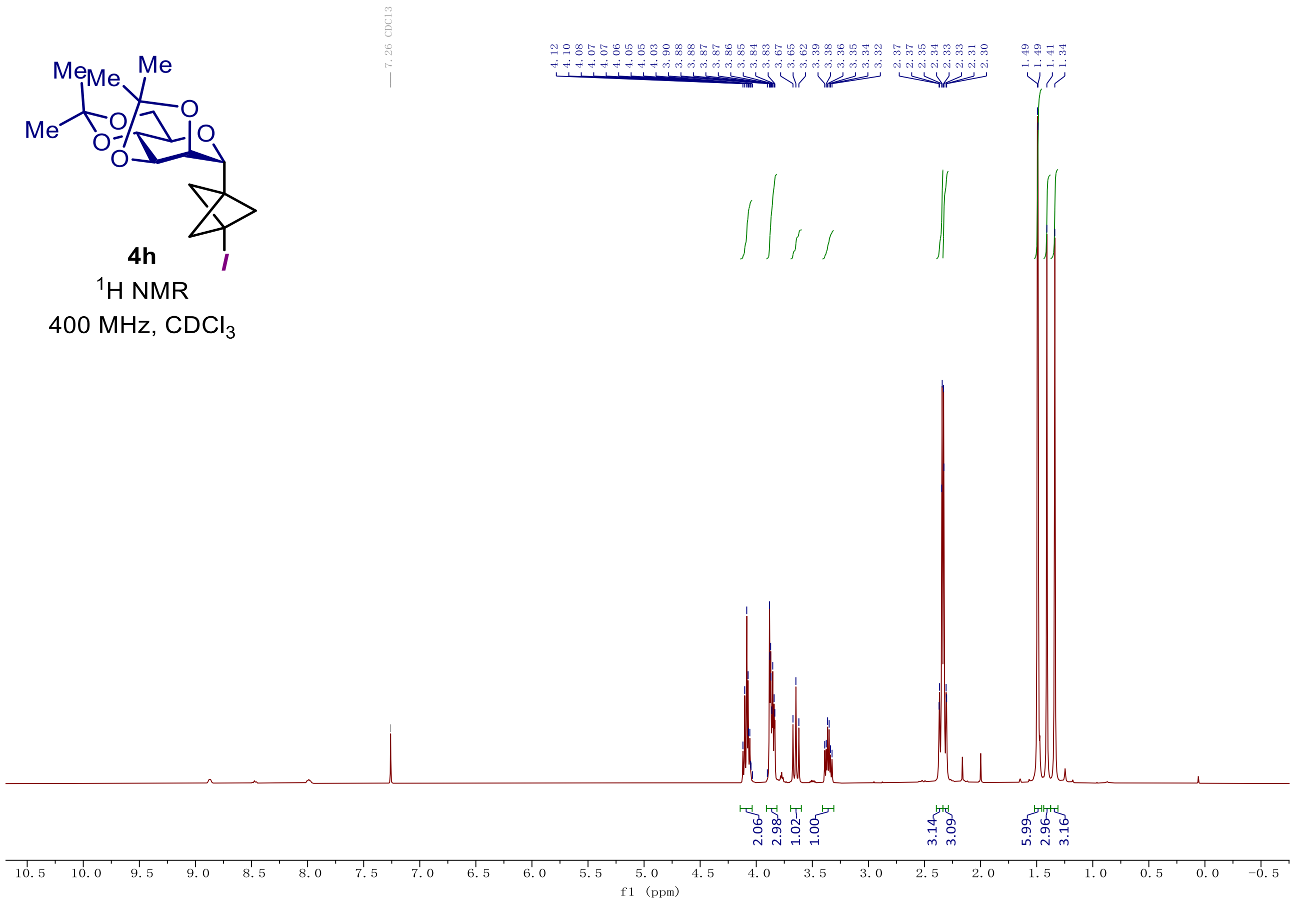
Me
Me
Me
Me
O
O
O
O
O
I
4h
^{1}H NMR
400 MHz, CDCl$_3$
7.26 CDCl3
2.06
2.98
1.02
1.00
3.14
3.09
5.99
2.96
3.16
f1 (ppm)

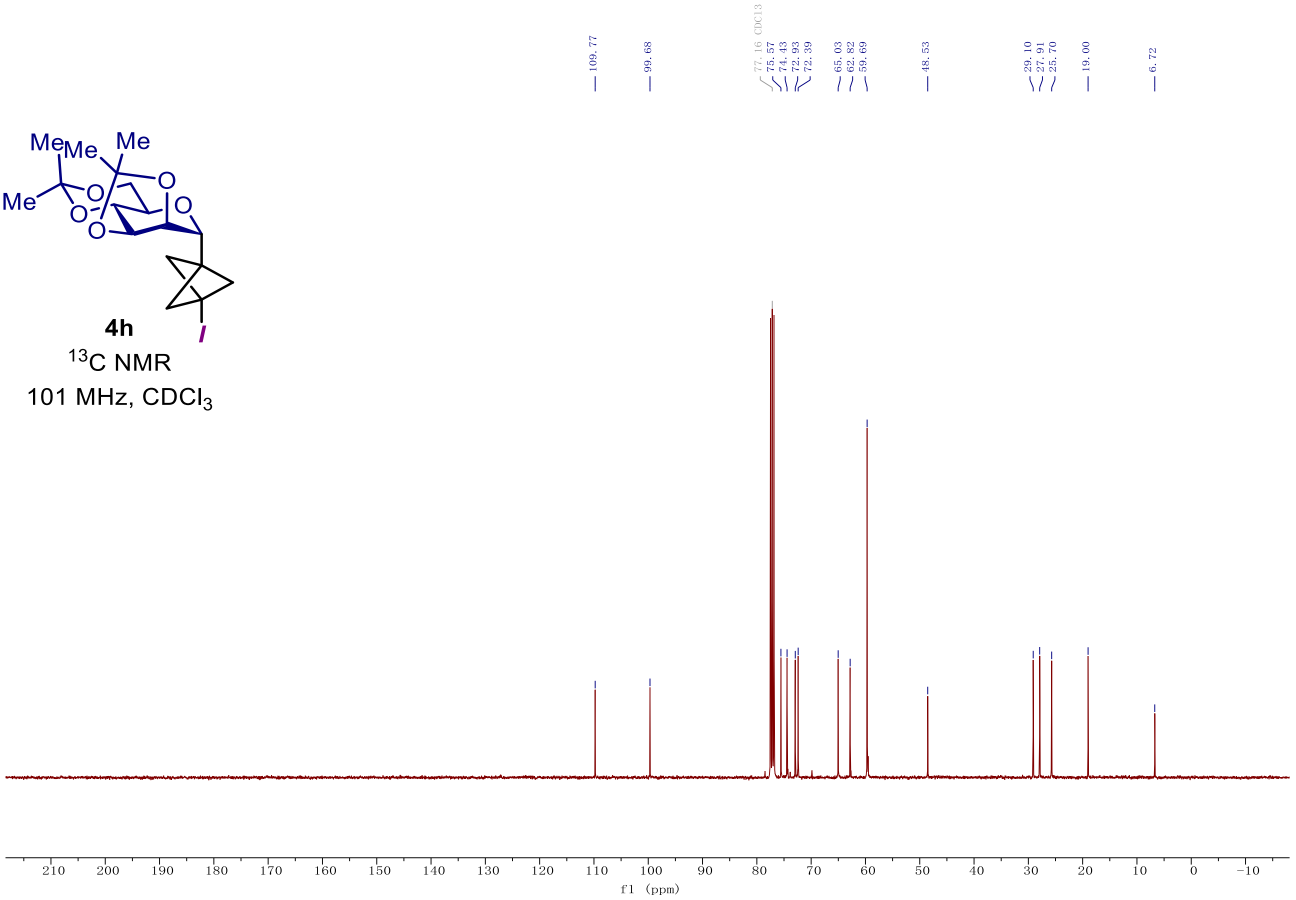
Me
Me
Me
Me
O
O
O
O
O
I
4h
^{13}C NMR
101 MHz, CDCl$_3$
109.77
99.68
77.16 CDCl3
75.57
74.43
72.93
72.39
65.03
62.82
59.69
48.53
29.10
27.91
25.70
19.00
6.72
f1 (ppm)

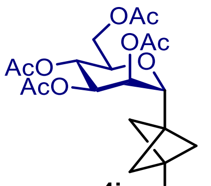

4i

^{1}H NMR

400 MHz, $CDCl_3$

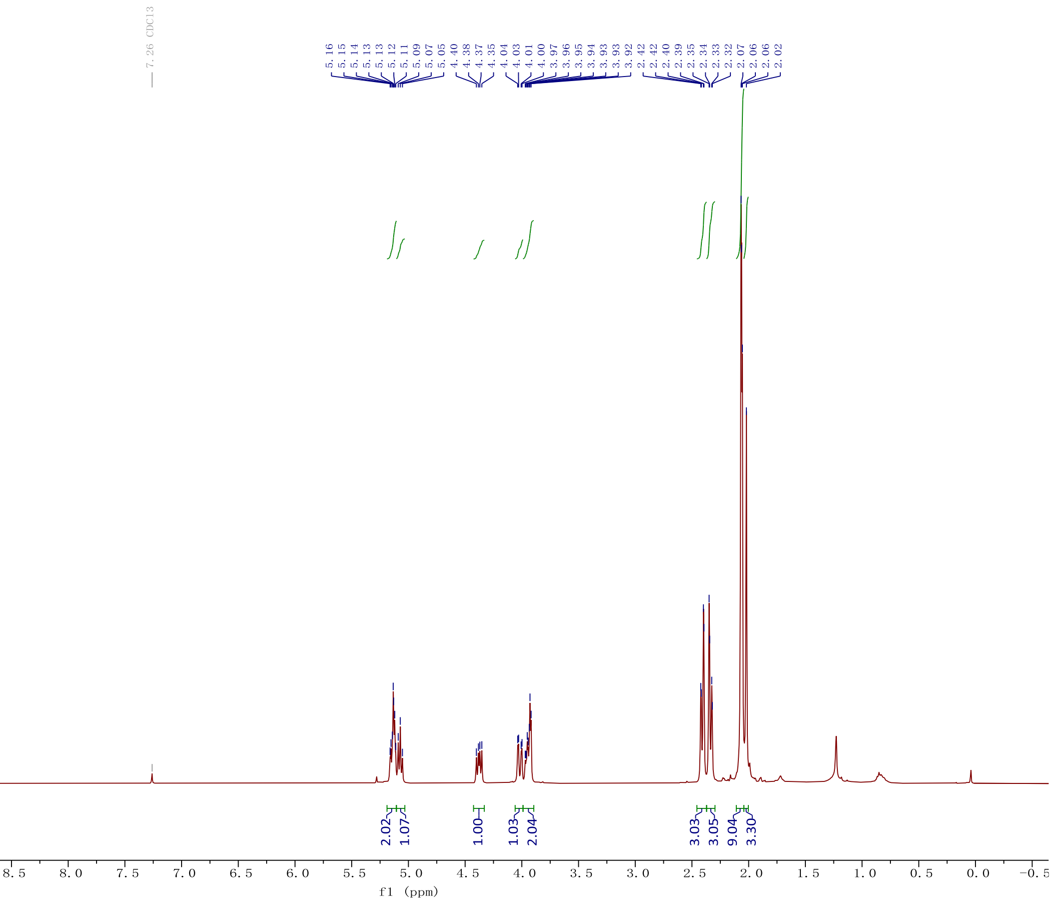

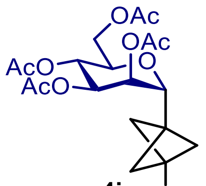

4i

^{13}C NMR

101 MHz, $CDCl_3$

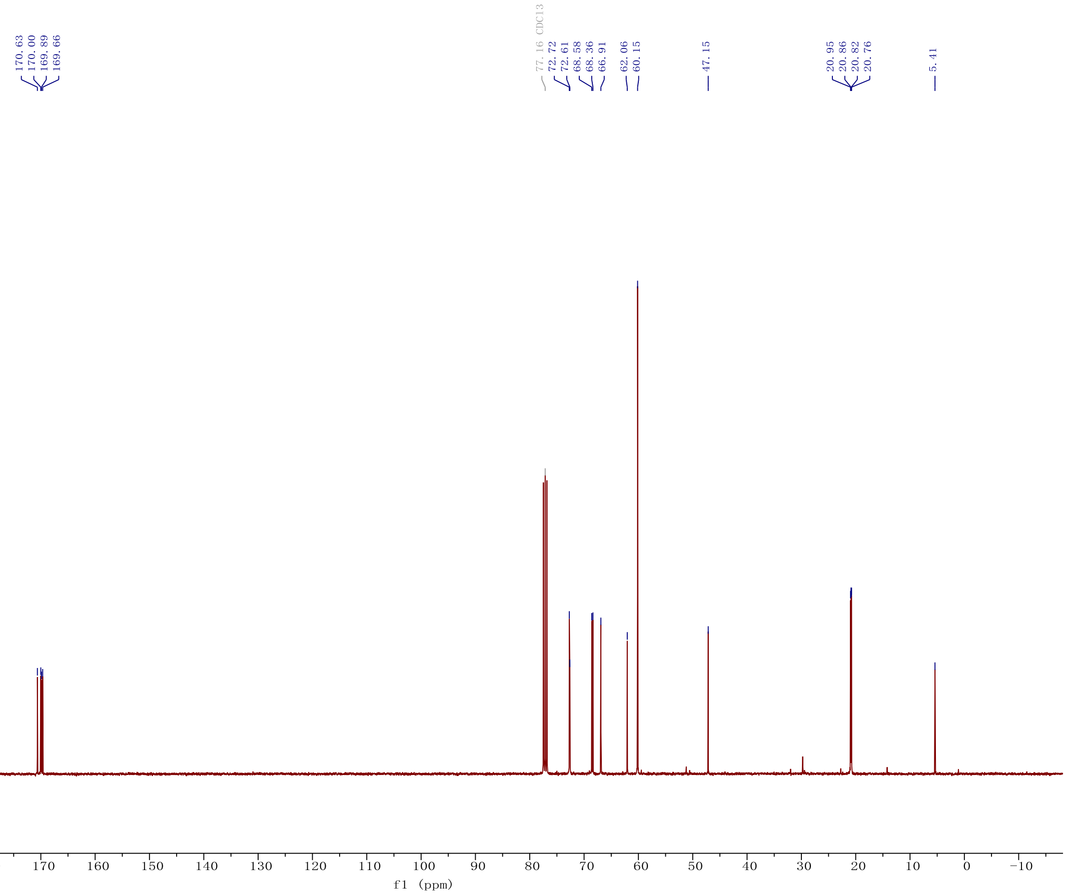

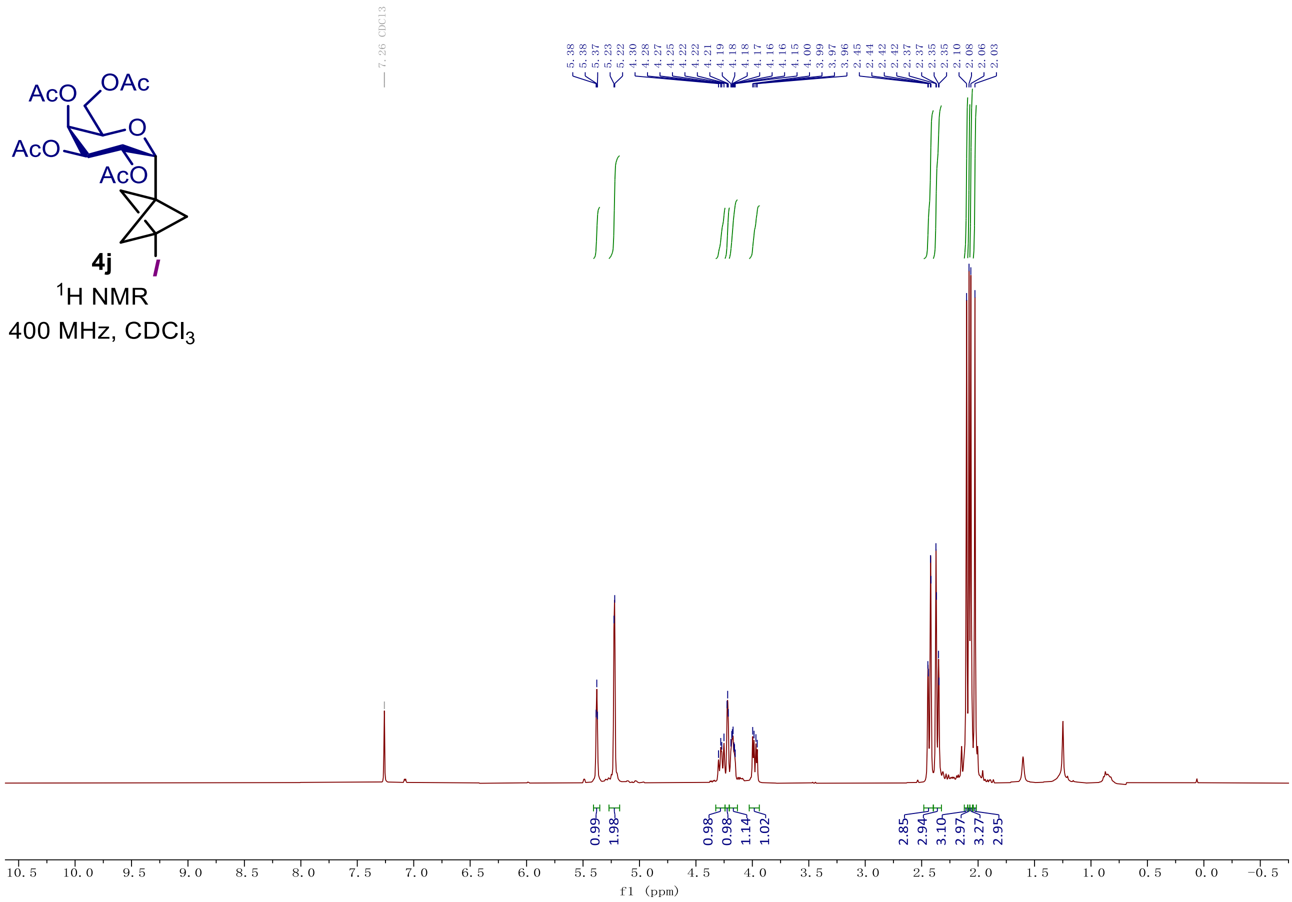
AcO
OAc
AcO
AcO
O
I
4j
1H NMR
400 MHz, CDCl3
7.26 CDCl3
5.38 5.38 5.37 5.23 5.22 4.30 4.28 4.27 4.25 4.22 4.22 4.21 4.19 4.18 4.18 4.17 4.16 4.16 4.15 4.00 3.99 3.97 3.96 2.45 2.44 2.42 2.42 2.37 2.37 2.35 2.35 2.10 2.08 2.06 2.03
0.99 1.98 0.98 0.98 1.14 1.02 2.85 2.94 3.10 2.97 3.27 2.95
10.5 10.0 9.5 9.0 8.5 8.0 7.5 7.0 6.5 6.0 5.5 5.0 4.5 4.0 3.5 3.0 2.5 2.0 1.5 1.0 0.5 0.0 -0.5
f1 (ppm)

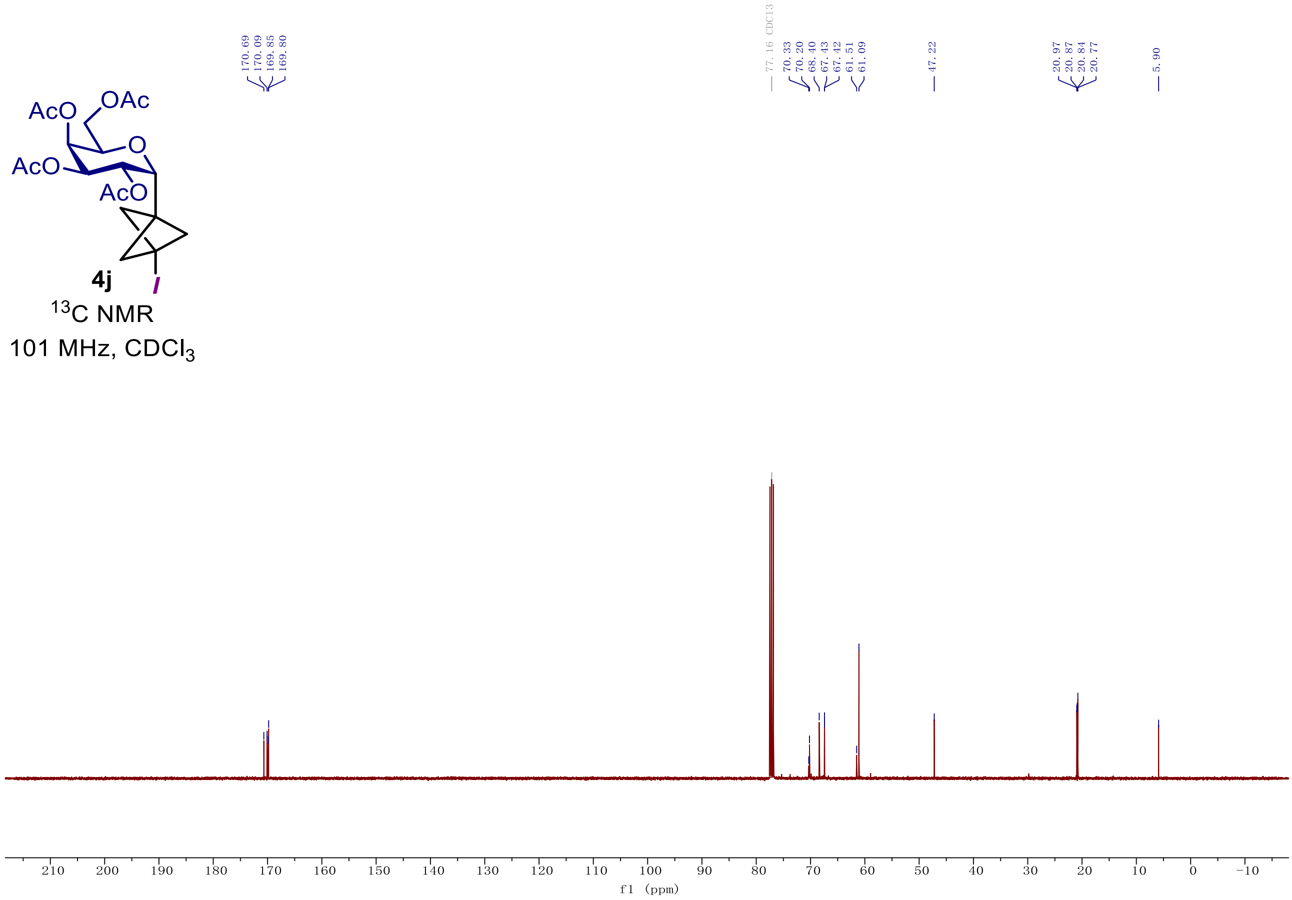
AcO
OAc
AcO
AcO
O
I
4j
13C NMR
101 MHz, CDCl3
170.69 170.09 169.85 169.80
77.16 CDCl3
70.33 70.20 68.40 67.43 67.42 61.51 61.09
47.22
20.97 20.87 20.84 20.77
5.90
210 200 190 180 170 160 150 140 130 120 110 100 90 80 70 60 50 40 30 20 10 0 -10
f1 (ppm)

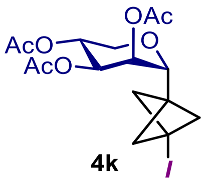

4k

^{1}H NMR

400 MHz, $CDCl_3$

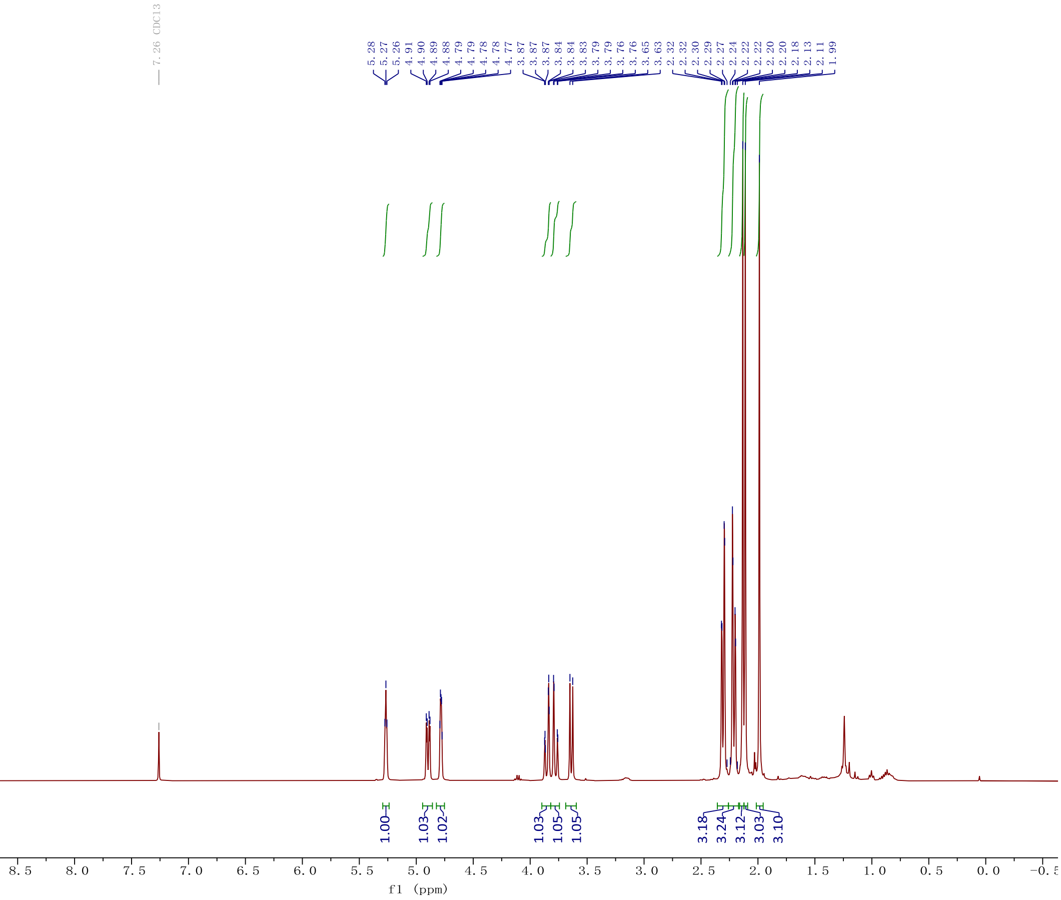

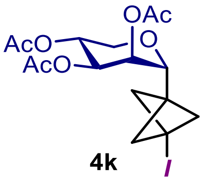

4k

^{13}C NMR

101 MHz, $CDCl_3$

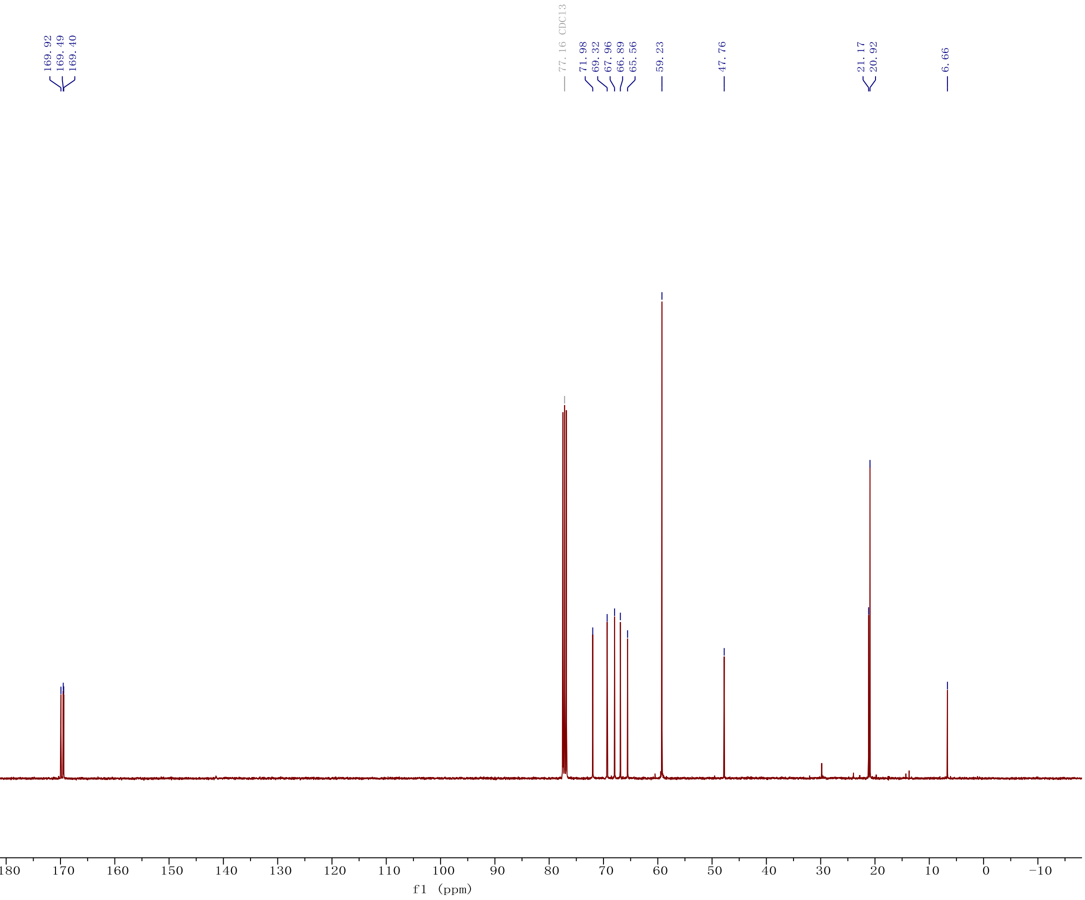

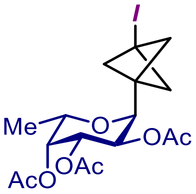

4l

^{1}H NMR

400 MHz, $CDCl_3$

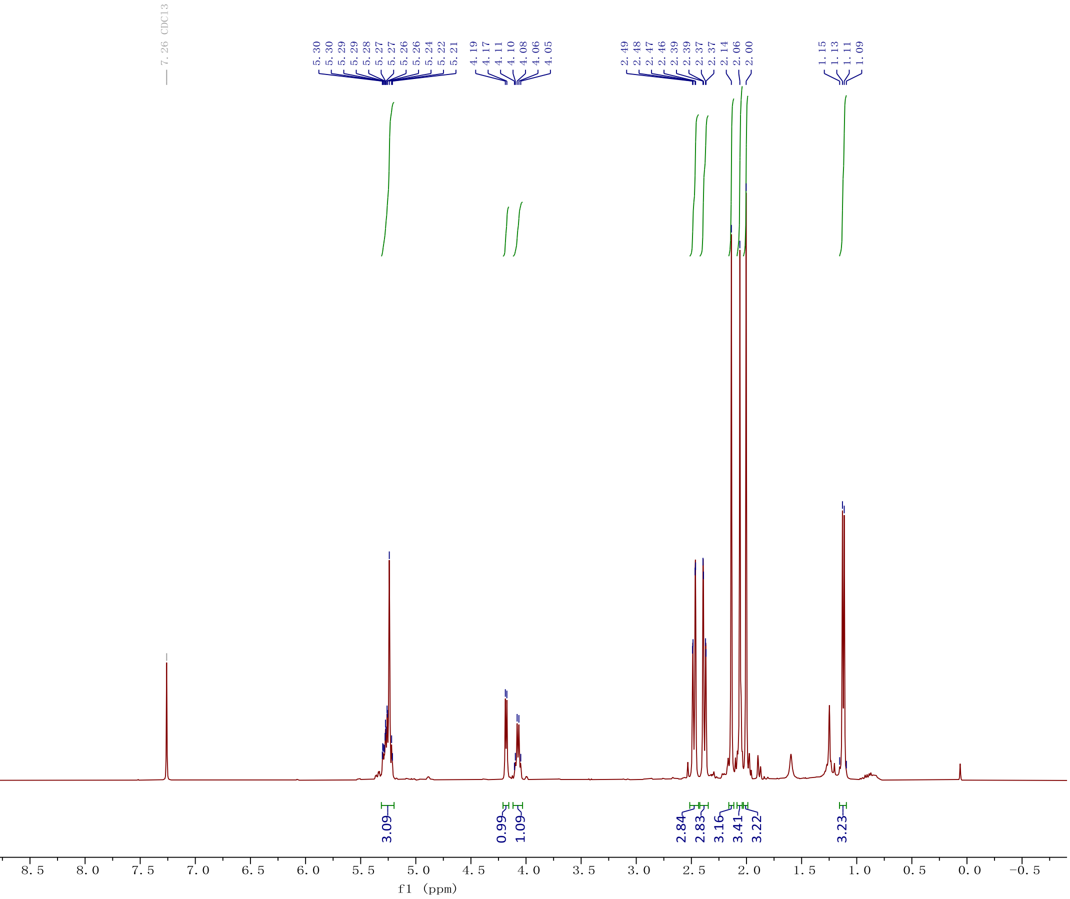

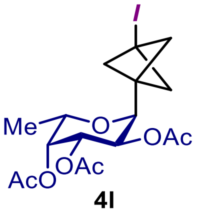

4l

^{13}C NMR

101 MHz, $CDCl_3$

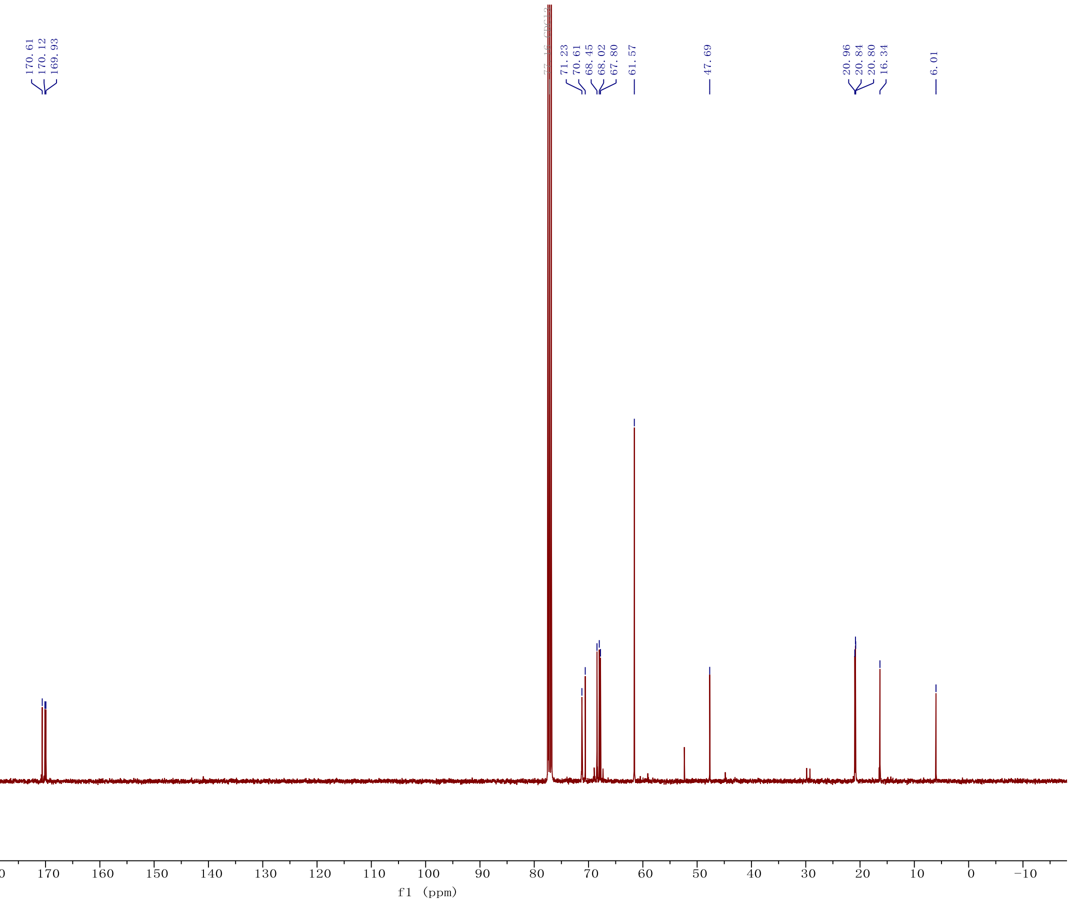

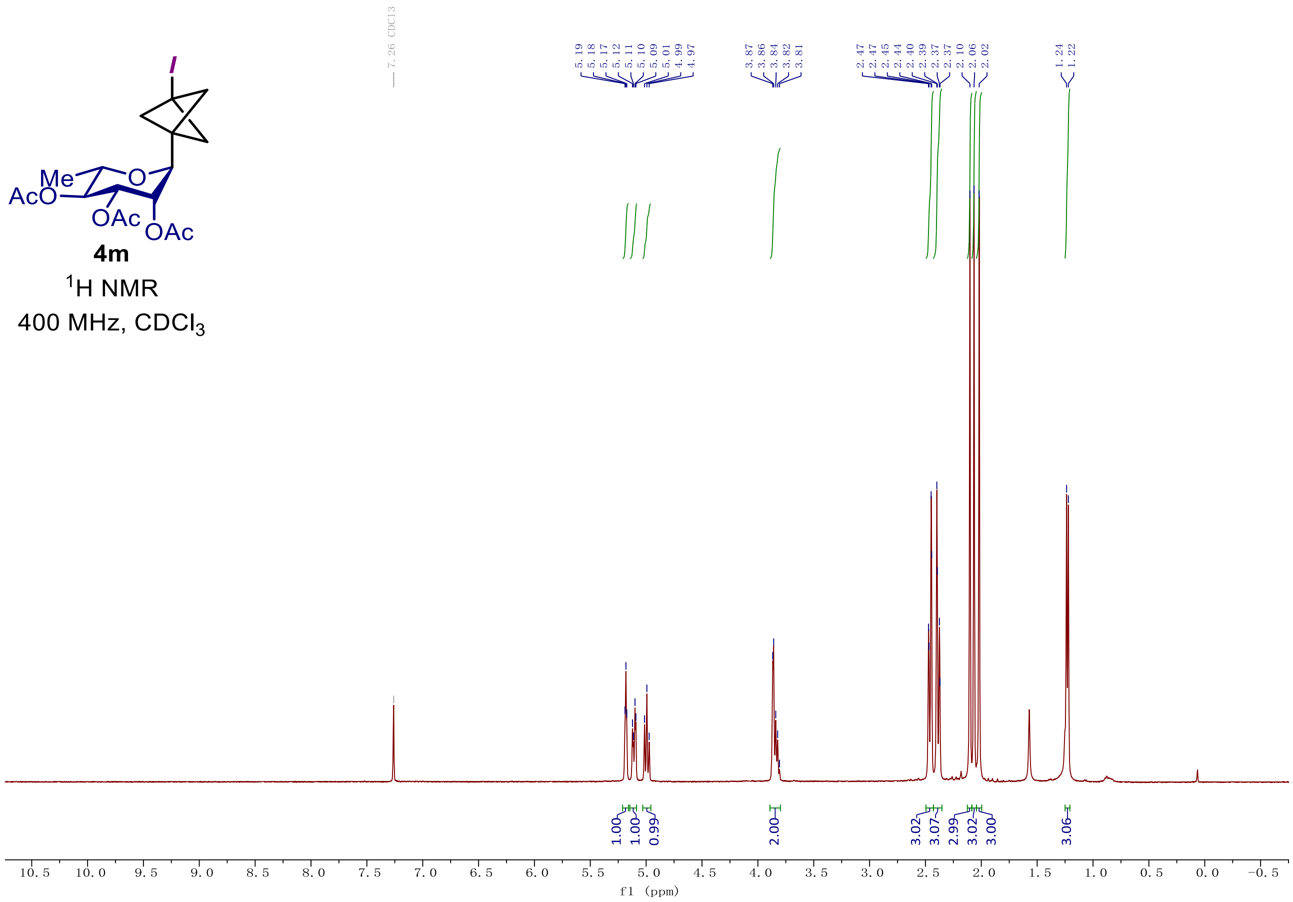
AcO
Me
O
OAc
OAc
I
4m
1H NMR
400 MHz, CDCl3
7.26 CDCl3
5.19 5.18 5.17 5.12 5.11 5.10 5.09 5.01 4.99 4.97
3.87 3.86 3.84 3.82 3.81
2.47 2.47 2.45 2.44 2.40 2.39 2.37 2.37 2.10 2.06 2.02
1.24 1.22
1.00 1.00 0.99 2.00 3.02 3.07 2.99 3.02 3.00 3.06
f1 (ppm)

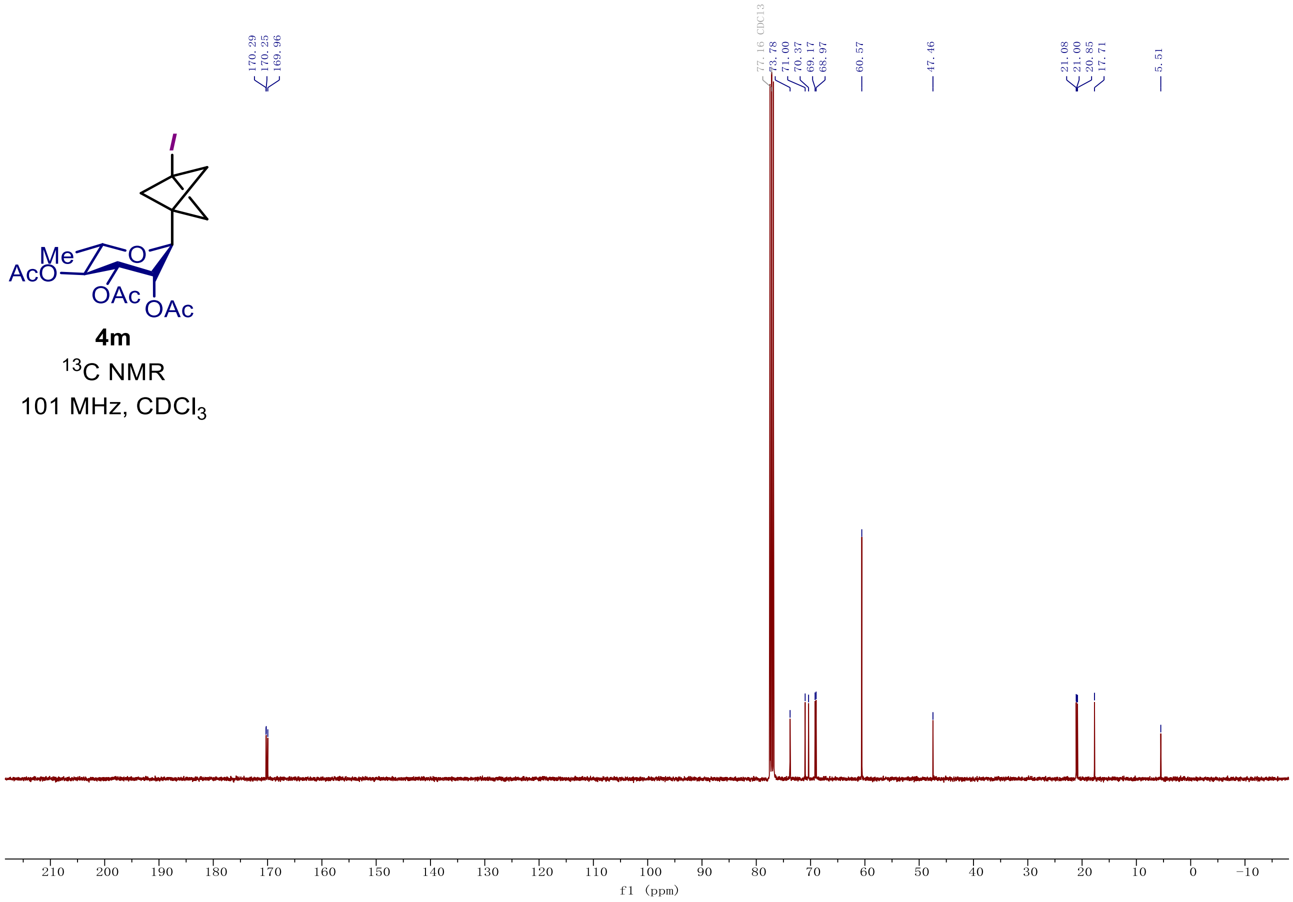
AcO
Me
O
OAc
OAc
I
4m
13C NMR
101 MHz, CDCl3
170.29 170.25 169.96
77.16 CDCl3
73.78 71.00 70.37 69.17 68.97
60.57
47.46
21.08 21.00 20.85 17.71
5.51
f1 (ppm)

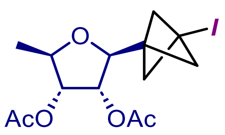

4n

^{1}H NMR

400 MHz, $CDCl_3$

β:α 10:1

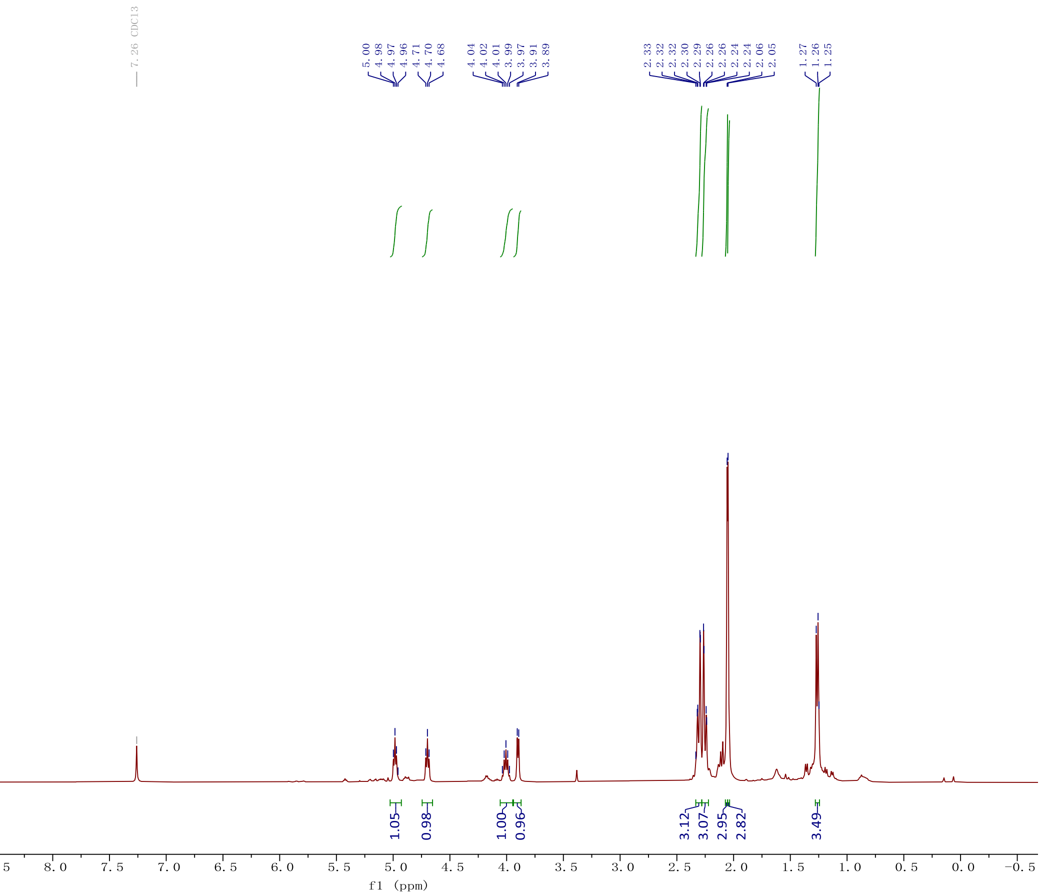

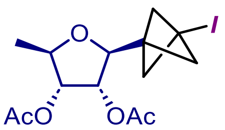

4n

^{13}C NMR

101 MHz, $CDCl_3$

β:α 10:1

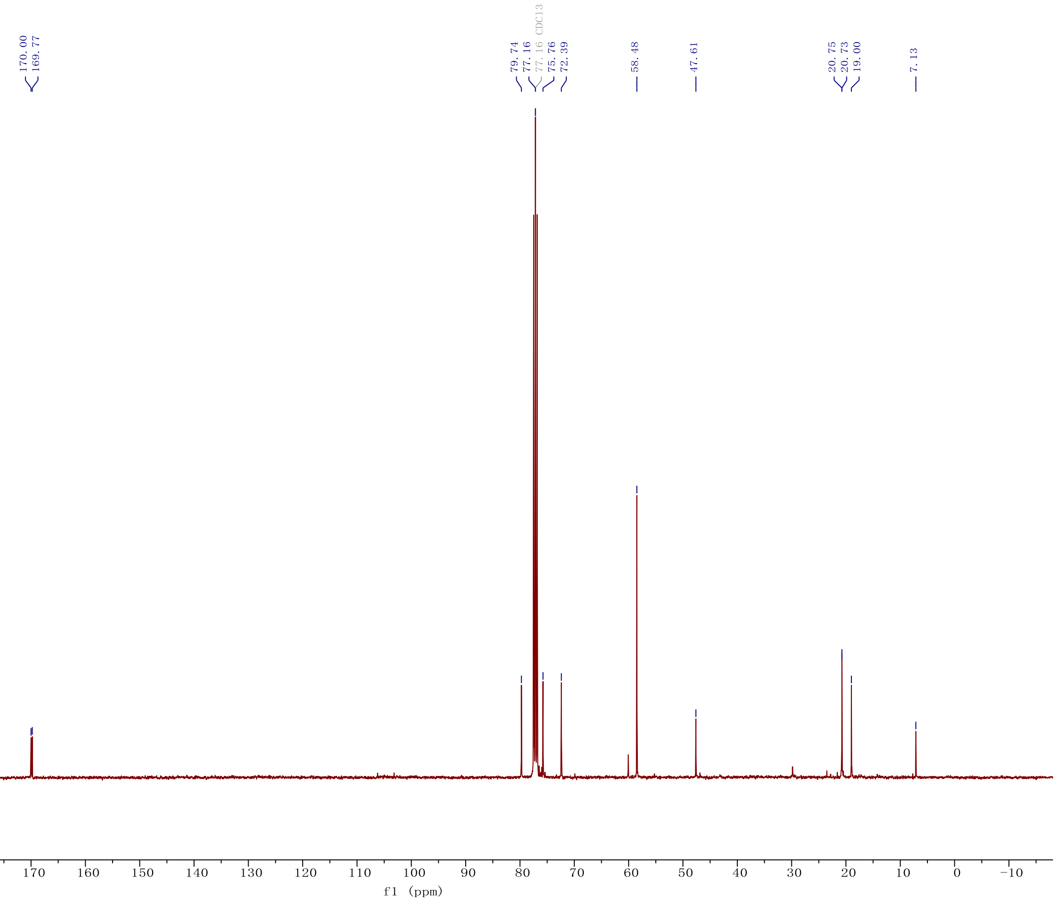

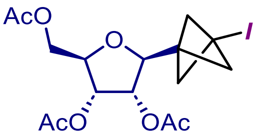

4o

^{1}H NMR

400 MHz, $CDCl_3$

β:α 13:1

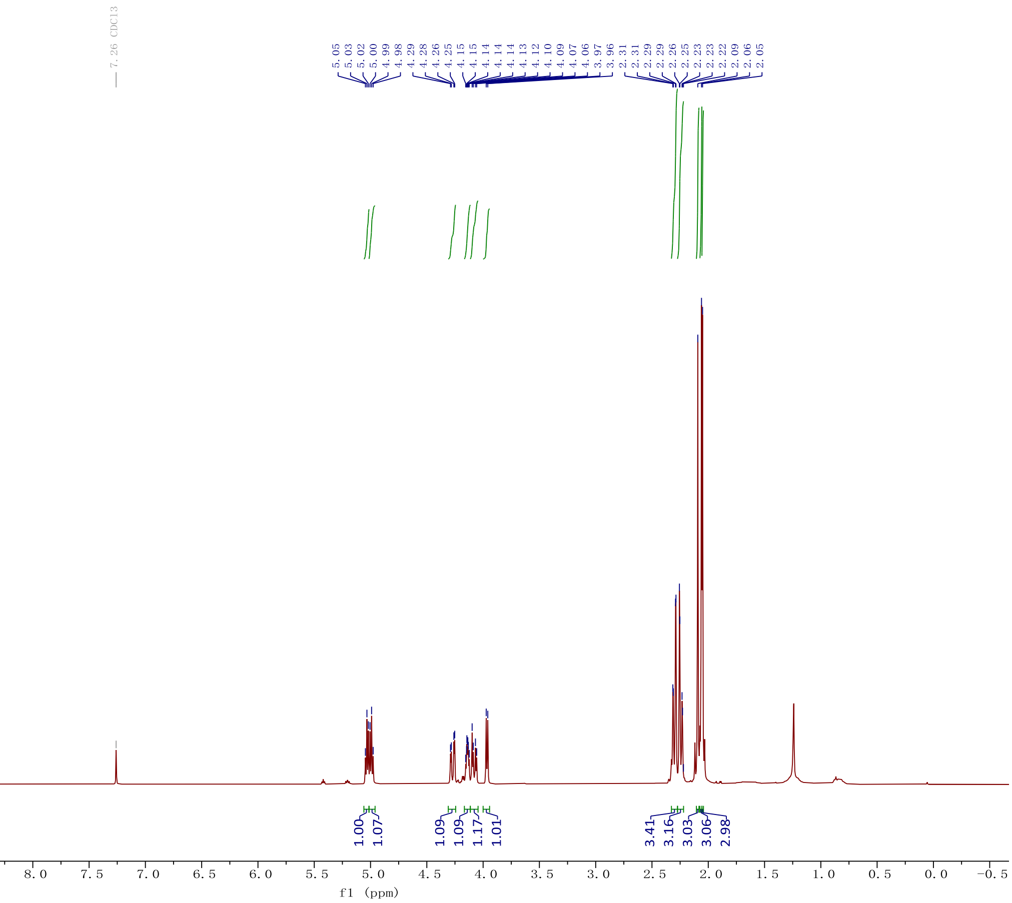

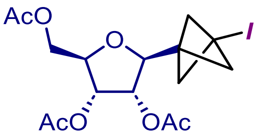

4o

^{13}C NMR

101 MHz, $CDCl_3$

β:α 13:1

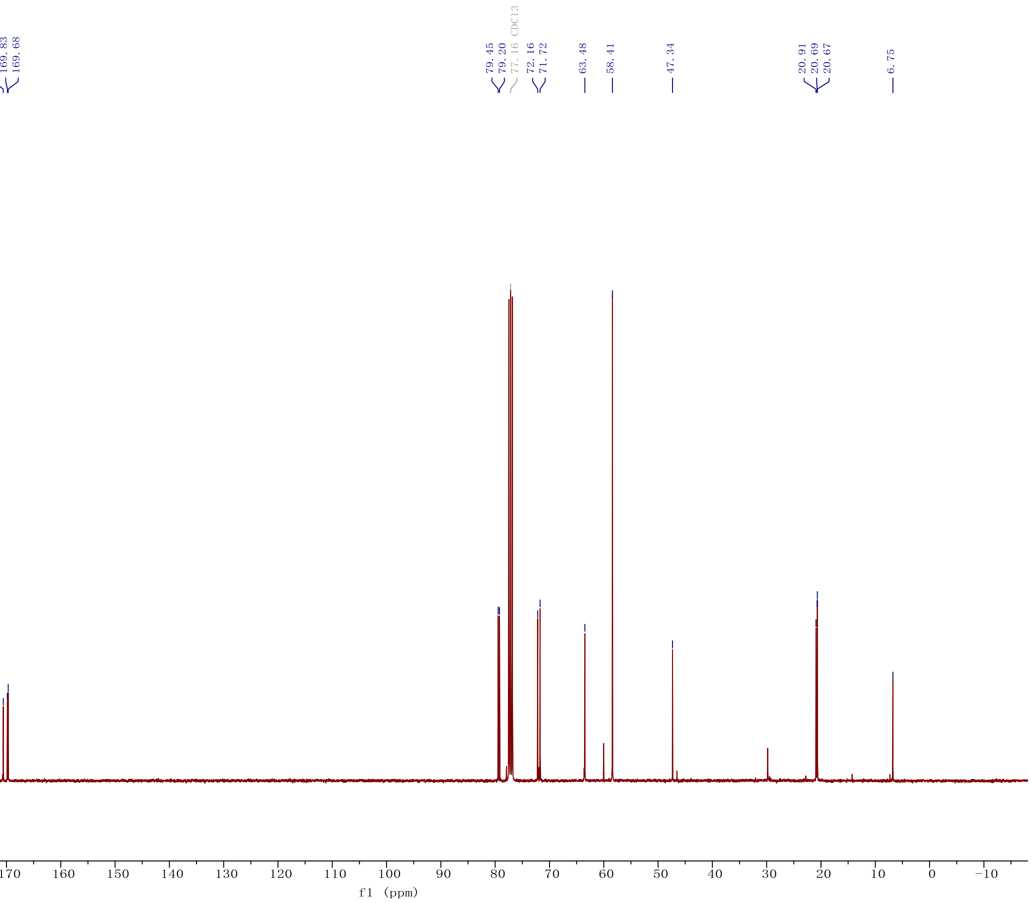

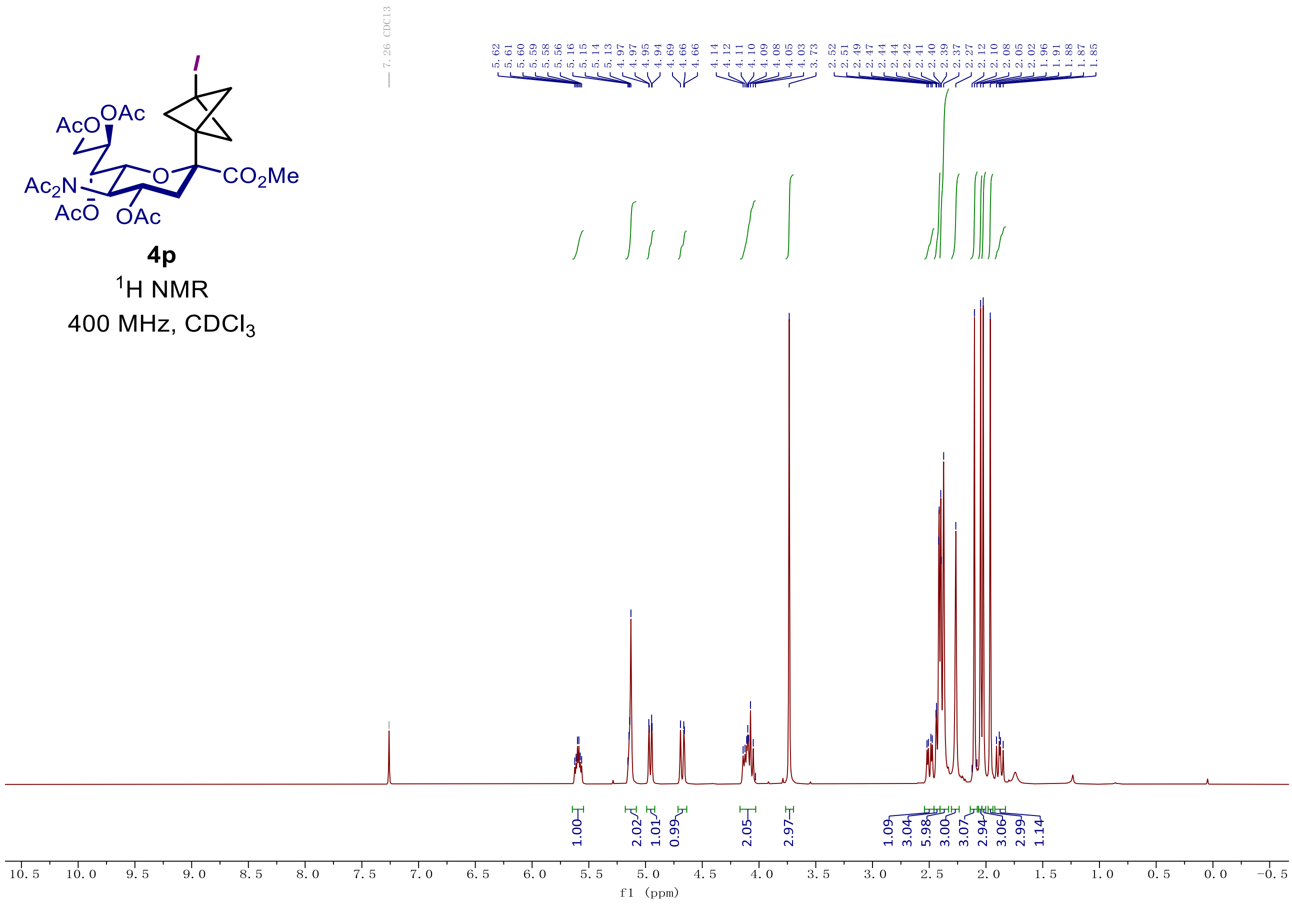
AcO OAc
Ac2N
AcO OAc
O
CO2Me
I
4p
1H NMR
400 MHz, CDCl3
7.26 CDCl3
5.62 5.61 5.60 5.59 5.58 5.56 5.16 5.15 5.14 5.13 4.97 4.95 4.94 4.69 4.66 4.66 4.14 4.12 4.11 4.10 4.09 4.08 4.05 4.03 3.73 2.52 2.51 2.49 2.47 2.44 2.44 2.42 2.41 2.40 2.39 2.37 2.27 2.12 2.10 2.08 2.05 2.02 1.96 1.91 1.88 1.87 1.85
1.00 2.02 1.01 0.99 2.05 2.97 1.09 3.04 5.98 3.00 3.07 2.94 3.06 2.99 1.14
f1 (ppm)

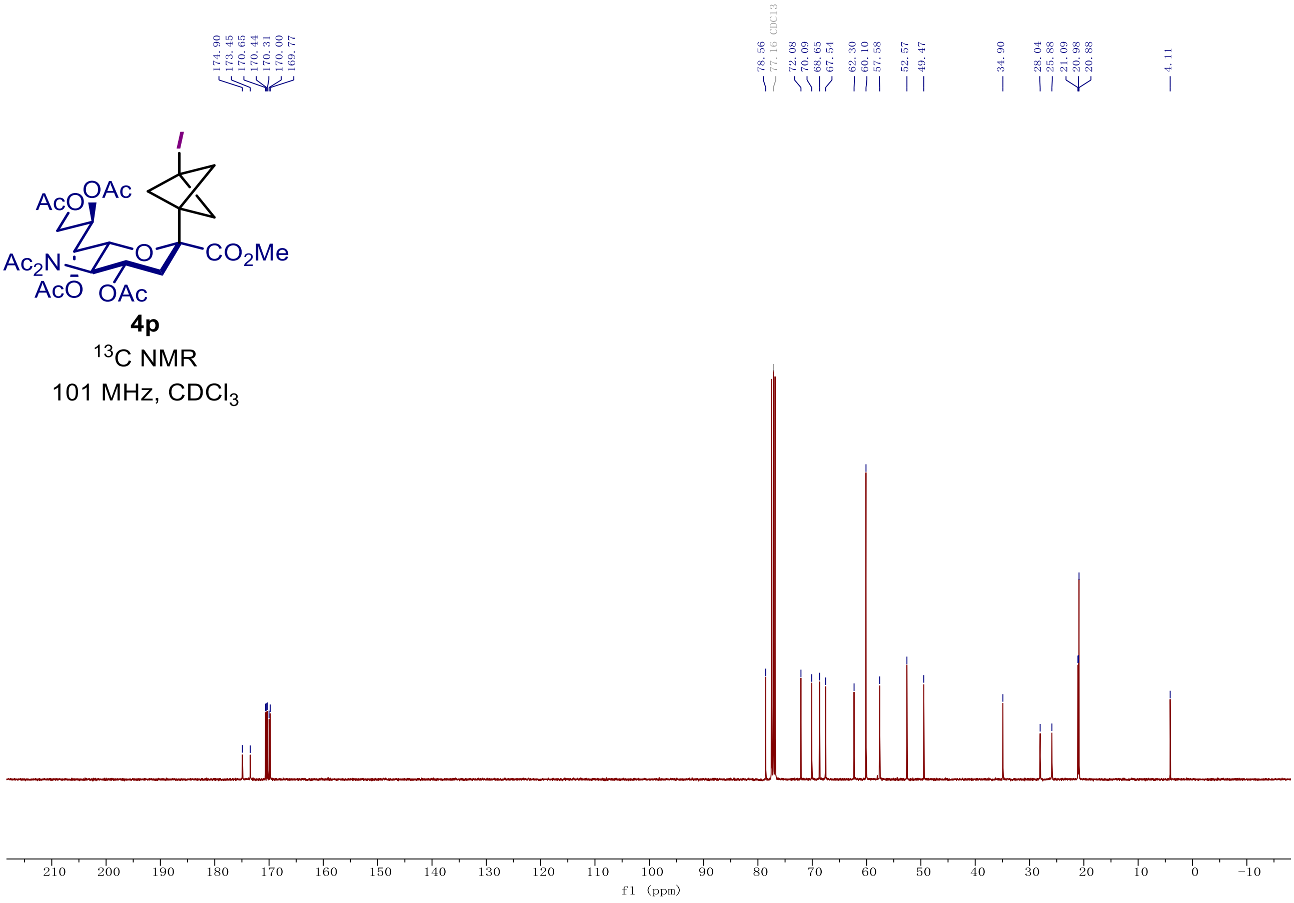
174.90 173.45 170.65 170.44 170.31 170.00 169.77
78.56 77.16 CDCl3 72.08 70.09 68.65 67.54 62.30 60.10 57.58 52.57 49.47 34.90 28.04 25.88 21.09 20.98 20.88 4.11
AcO OAc
Ac2N
AcO OAc
O
CO2Me
I
4p
13C NMR
101 MHz, CDCl3
f1 (ppm)

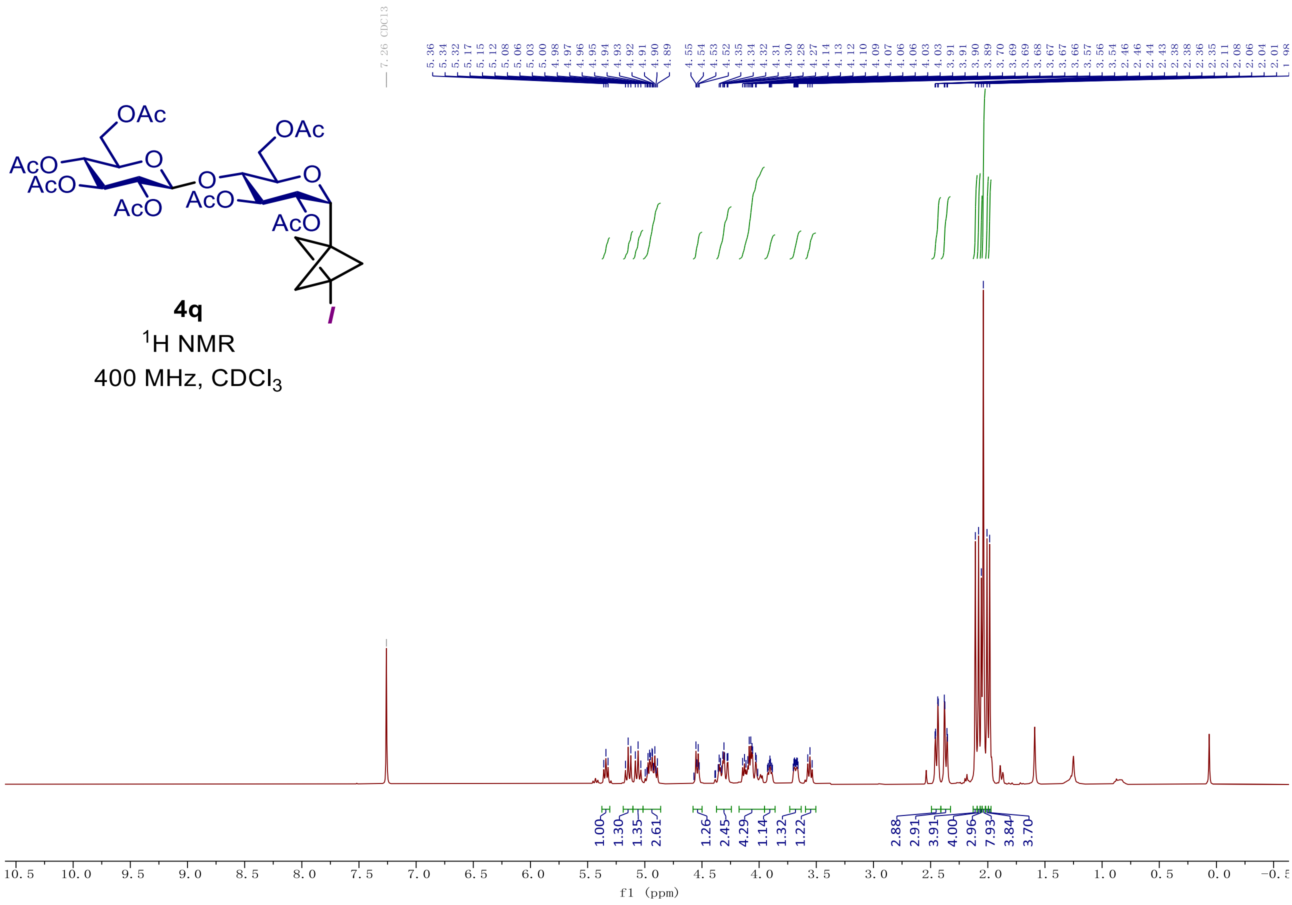

4q
^1H NMR
400 MHz, $CDCl_3$

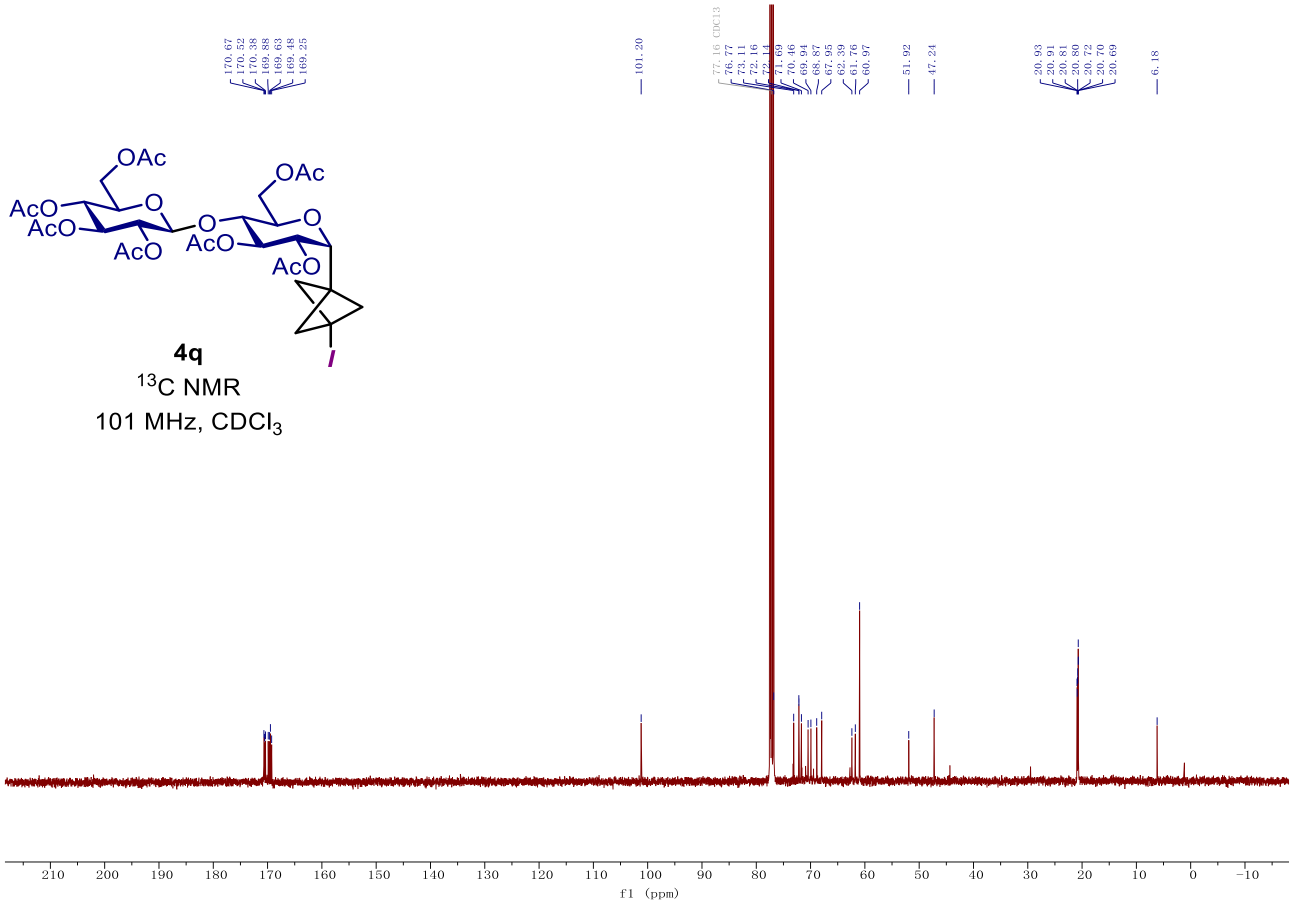

4q
^{13}C NMR
101 MHz, $CDCl_3$

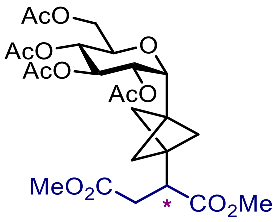

5a

^{1}H NMR

400 MHz, $CDCl_3$

1:1 d.r.

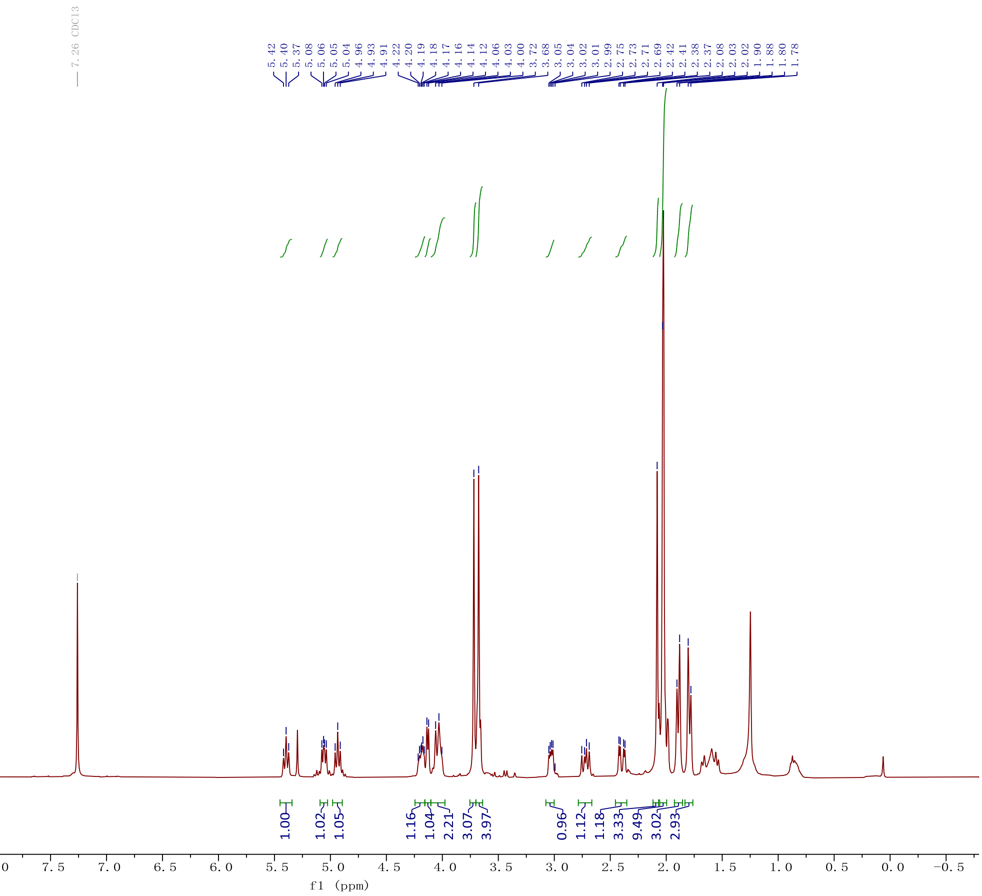

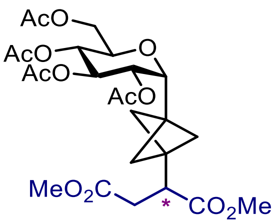

5a

^{13}C NMR

101 MHz, $CDCl_3$

1:1 d.r.

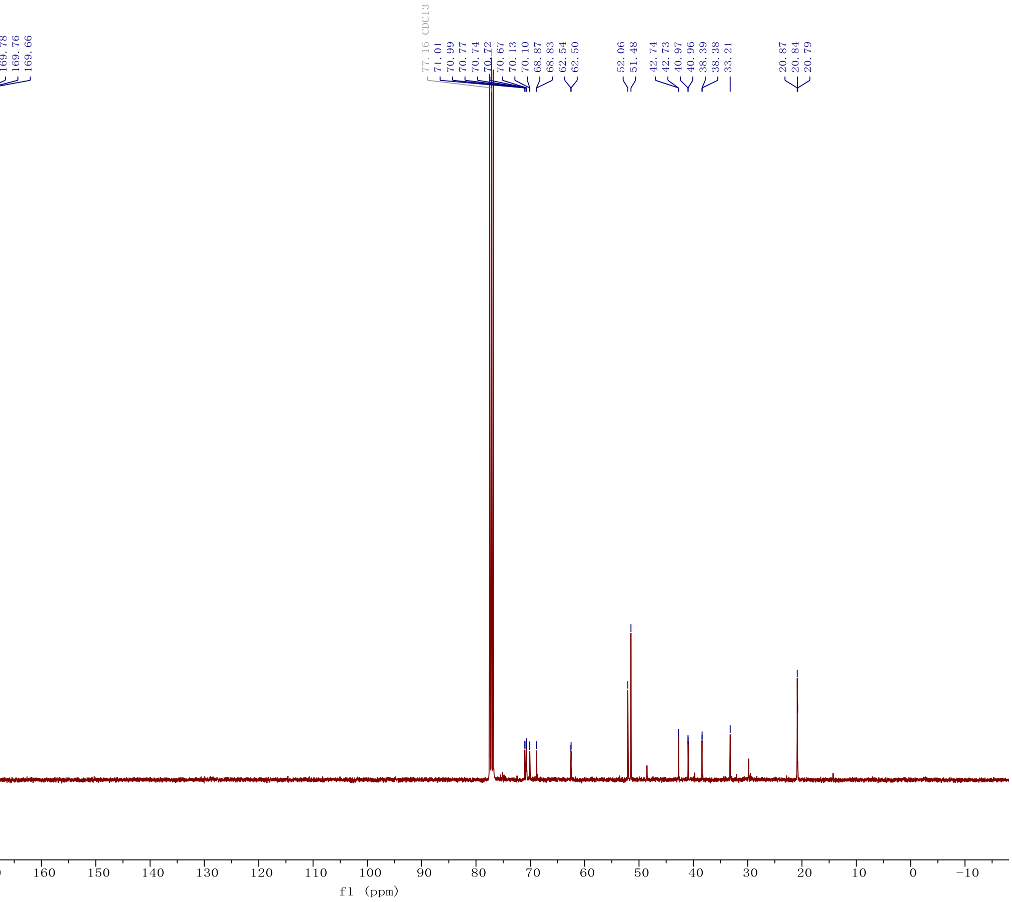

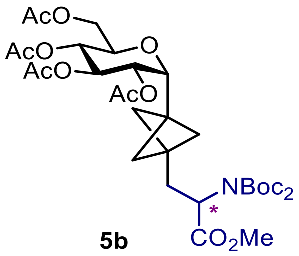

5b
^{1}H NMR
400 MHz, CDCl$_3$
1:1 d.r.

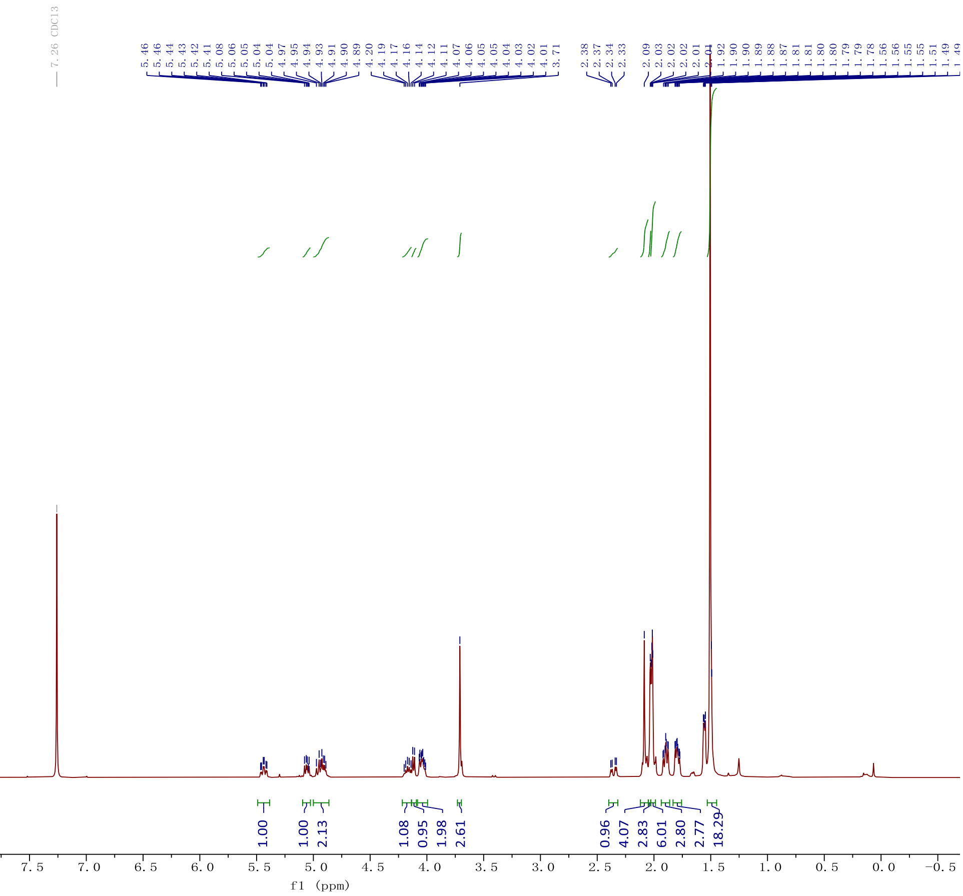

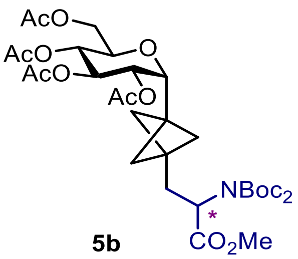

5b
^{13}C NMR
101 MHz, CDCl$_3$
1:1 d.r.

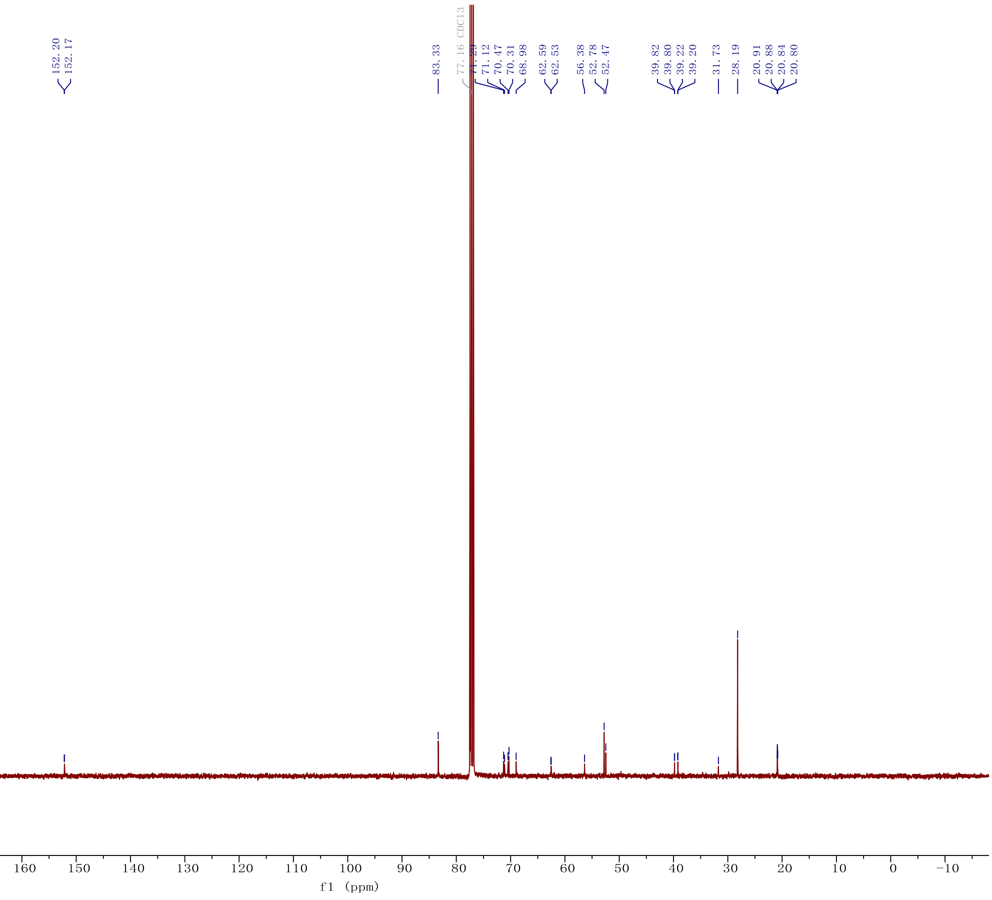

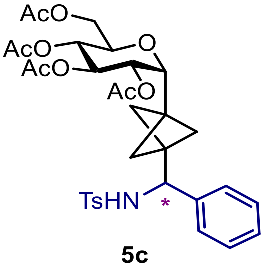

5c

^{1}H NMR

400 MHz, $CDCl_3$

1:1 d.r.

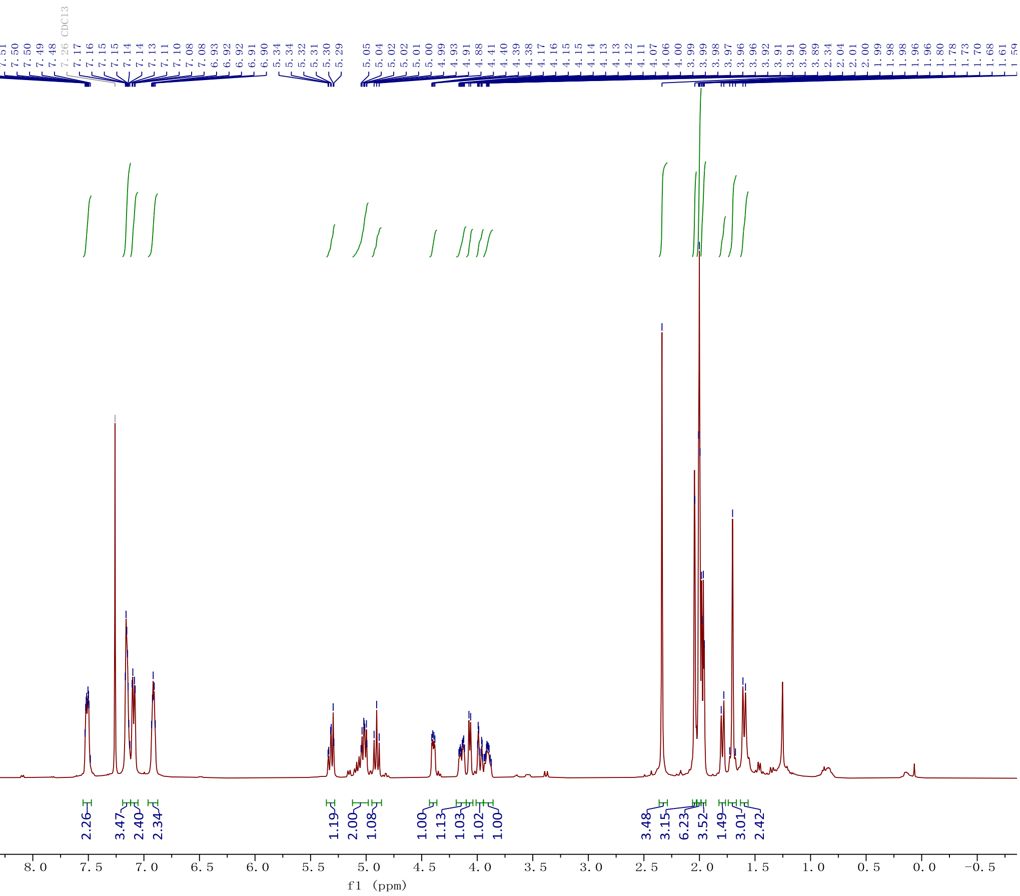

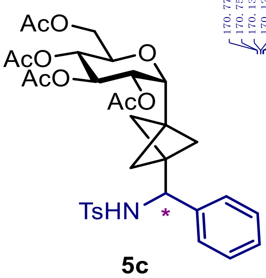

5c

^{13}C NMR

101 MHz, $CDCl_3$

1:1 d.r.

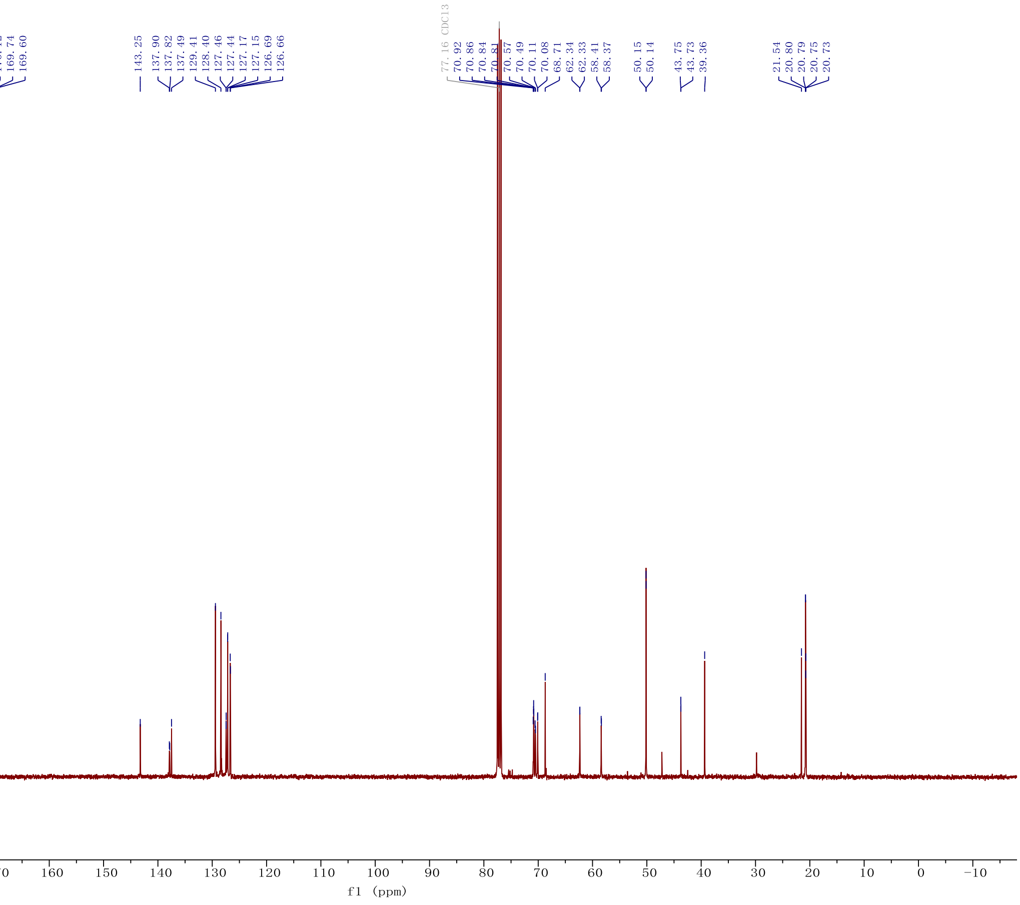

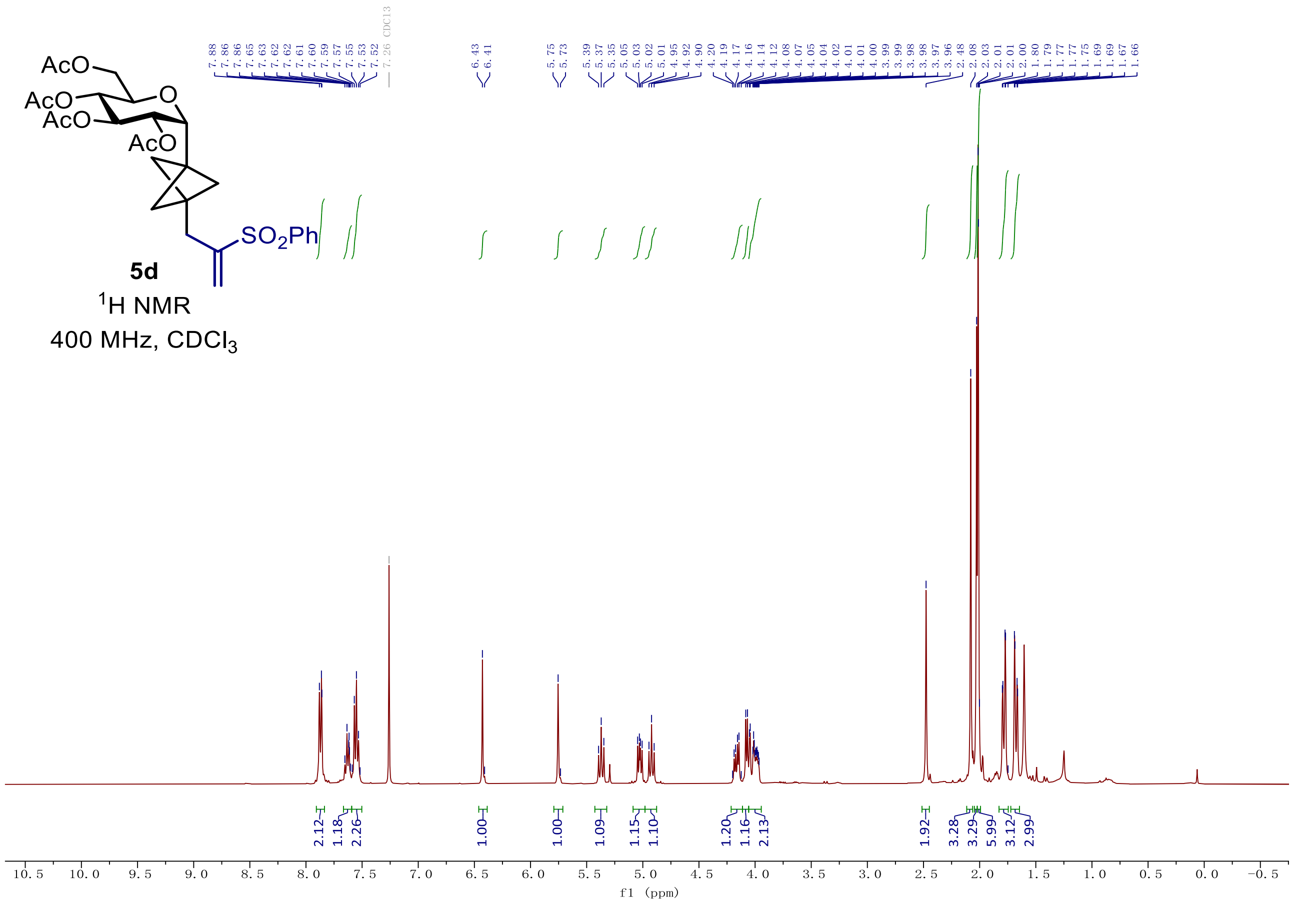
AcO
AcO
AcO
AcO
O
SO2Ph
5d
1H NMR
400 MHz, CDCl3
f1 (ppm)

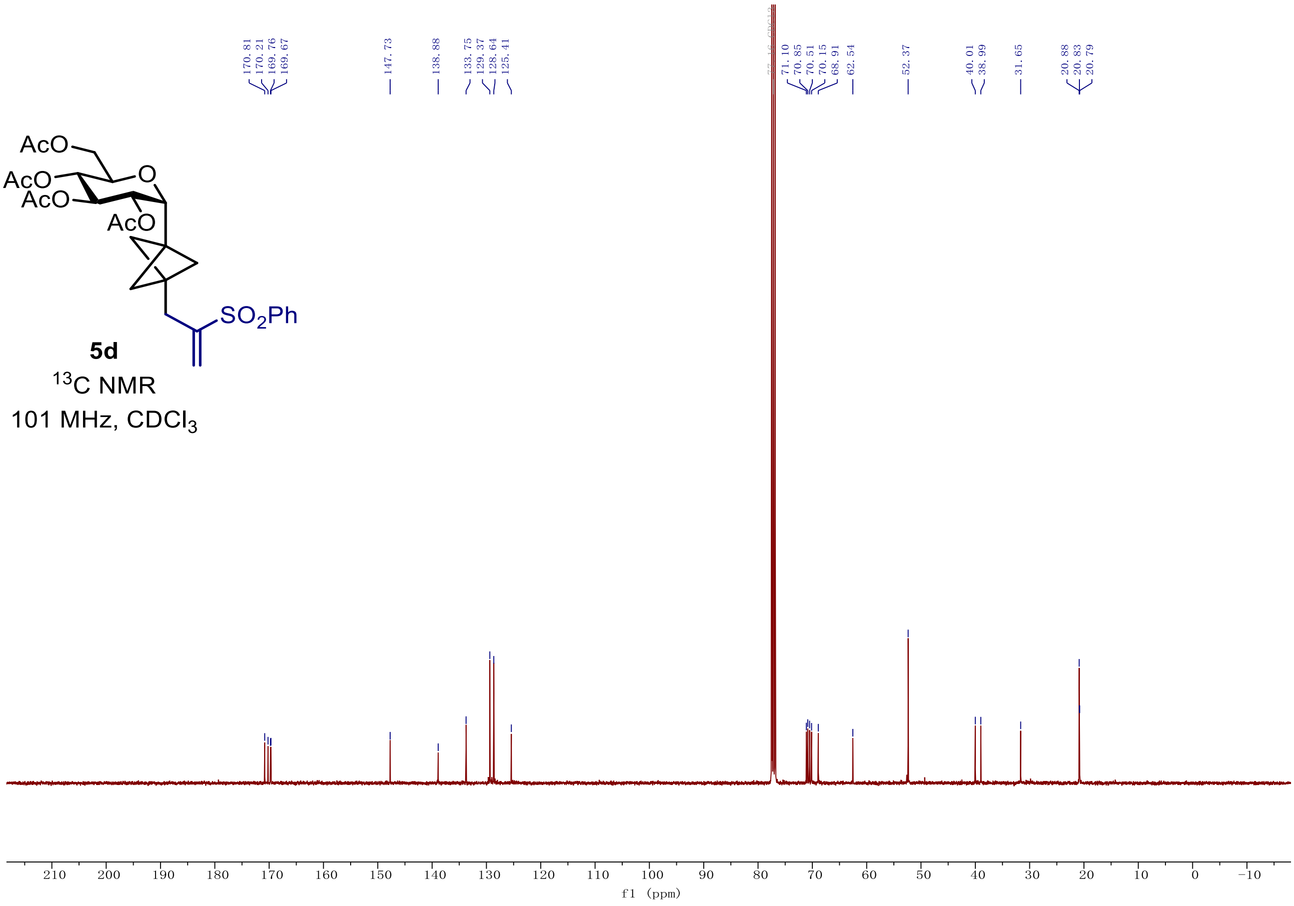
AcO
AcO
AcO
AcO
O
SO2Ph
5d
13C NMR
101 MHz, CDCl3
f1 (ppm)

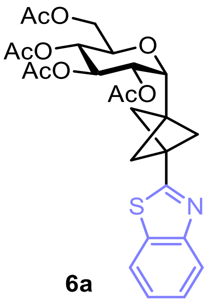

6a
^{1}H NMR
400 MHz, $CDCl_3$

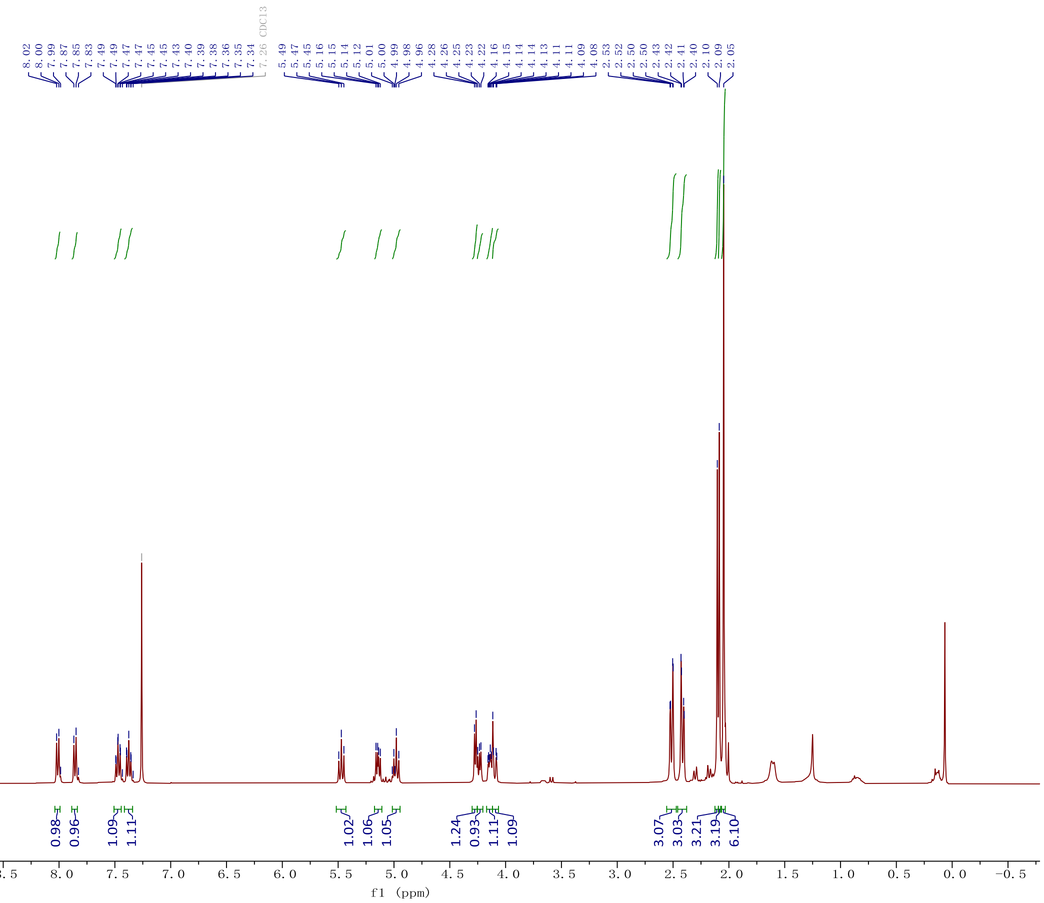

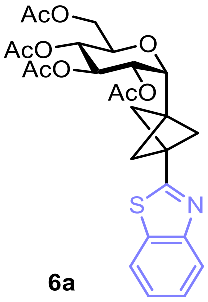

6a
^{13}C NMR
101 MHz, $CDCl_3$

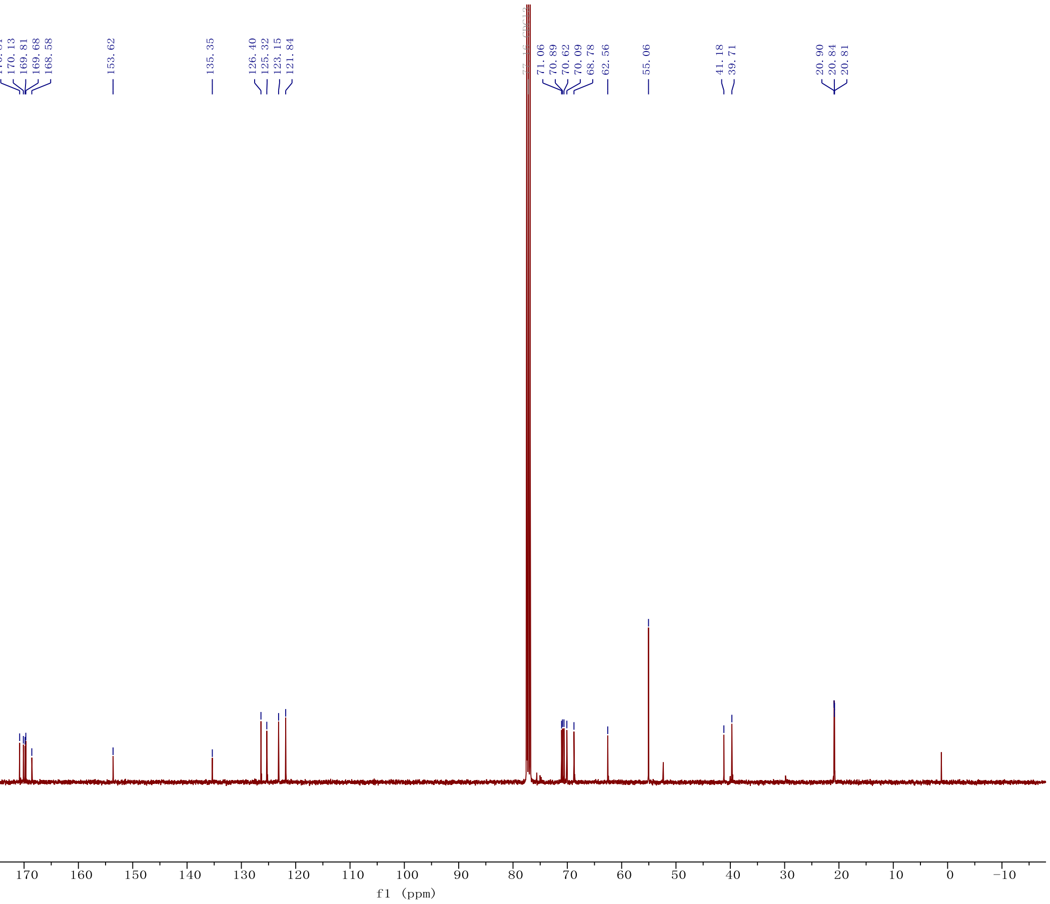

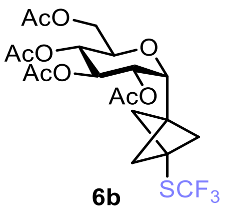

6b

^{1}H NMR

400 MHz, $CDCl_3$

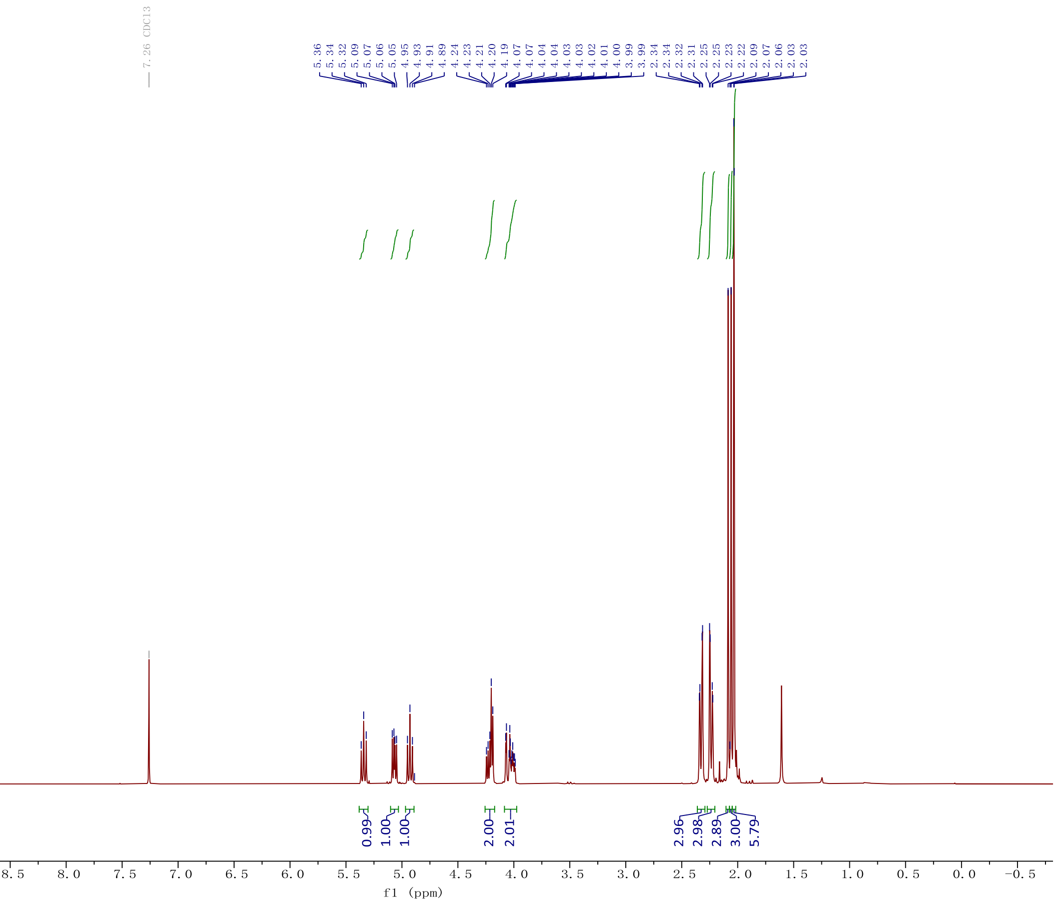

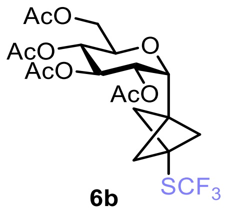

6b

^{13}C NMR

101 MHz, $CDCl_3$

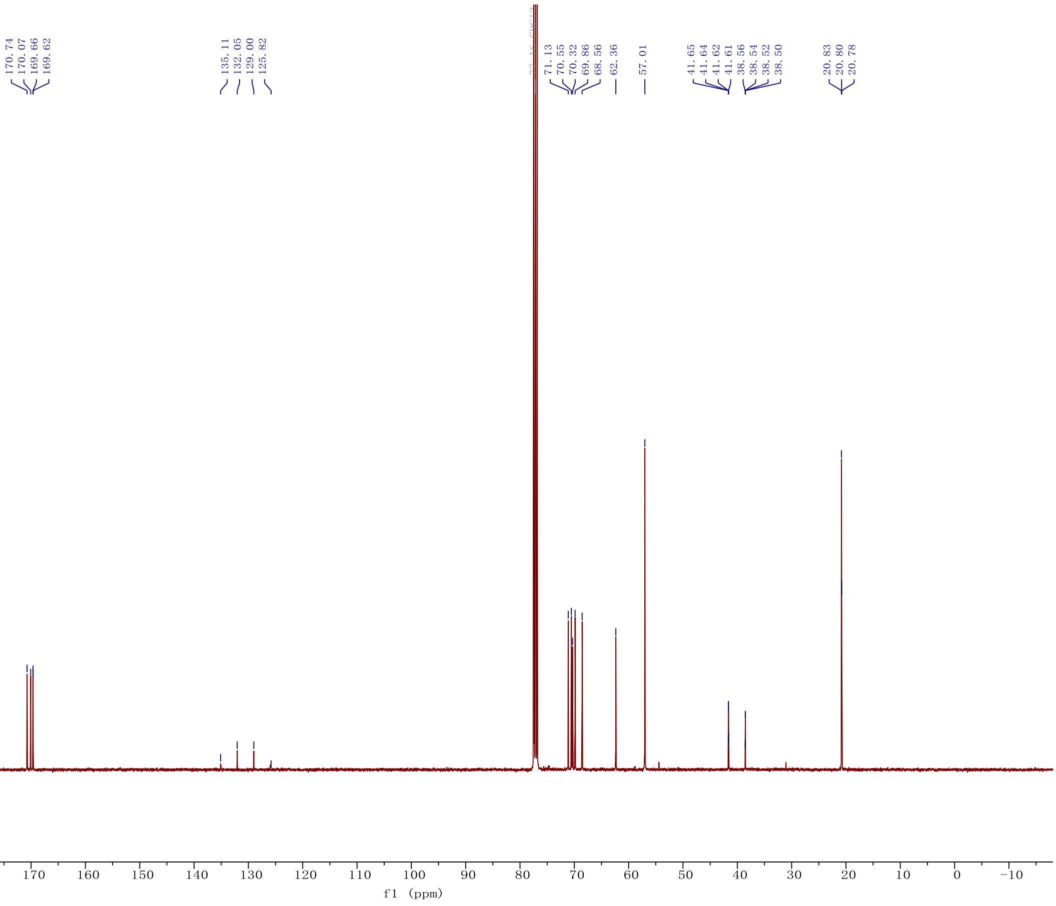

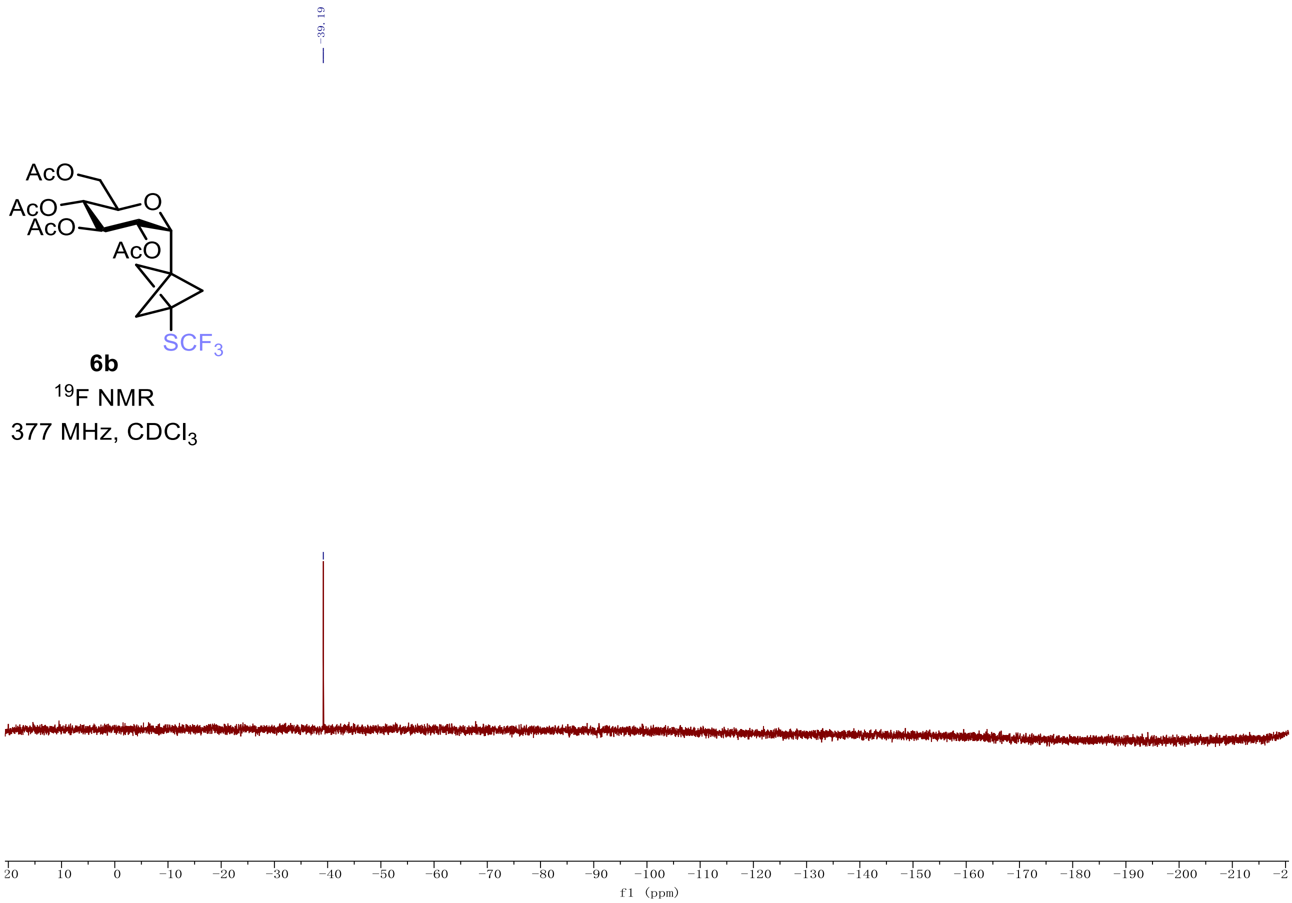

6b

^{19}F NMR

377 MHz, $CDCl_3$

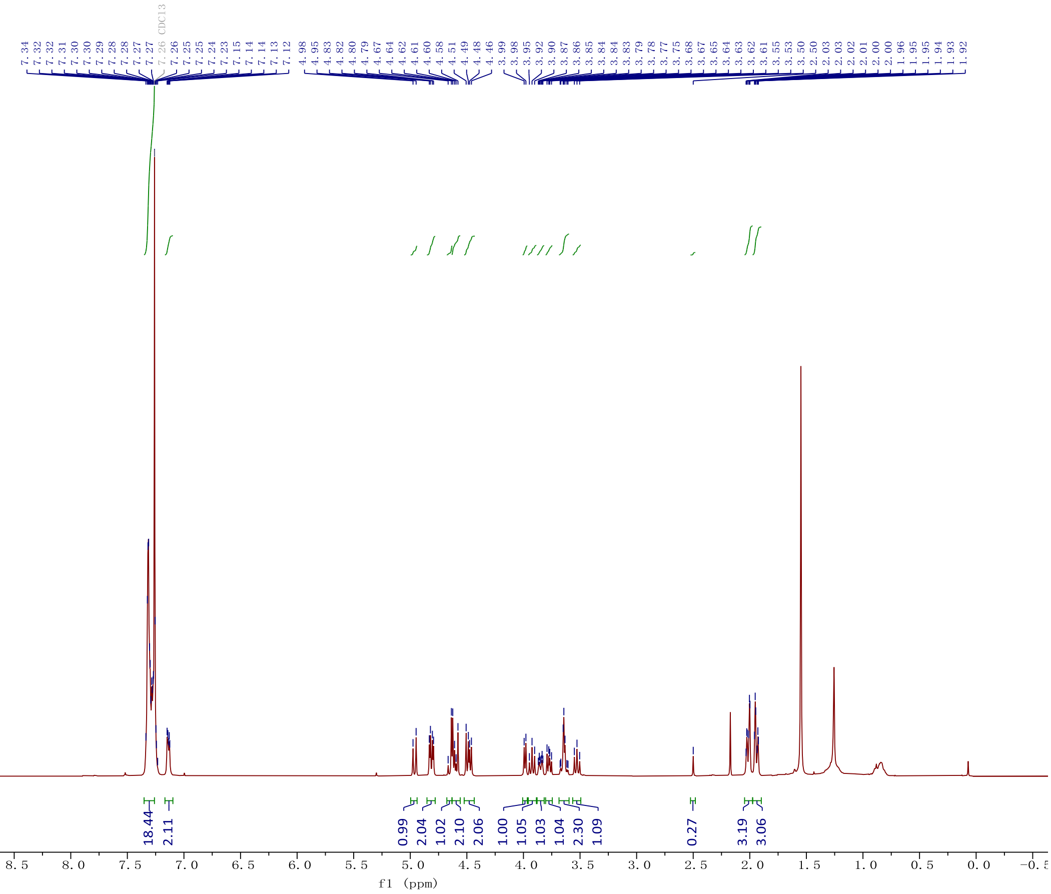

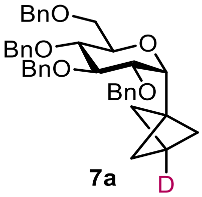

7a

^{1}H NMR

400 MHz, $CDCl_3$

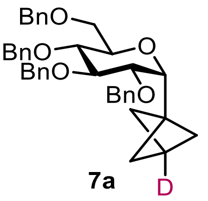

7a

^{13}C NMR

101 MHz, $CDCl_3$

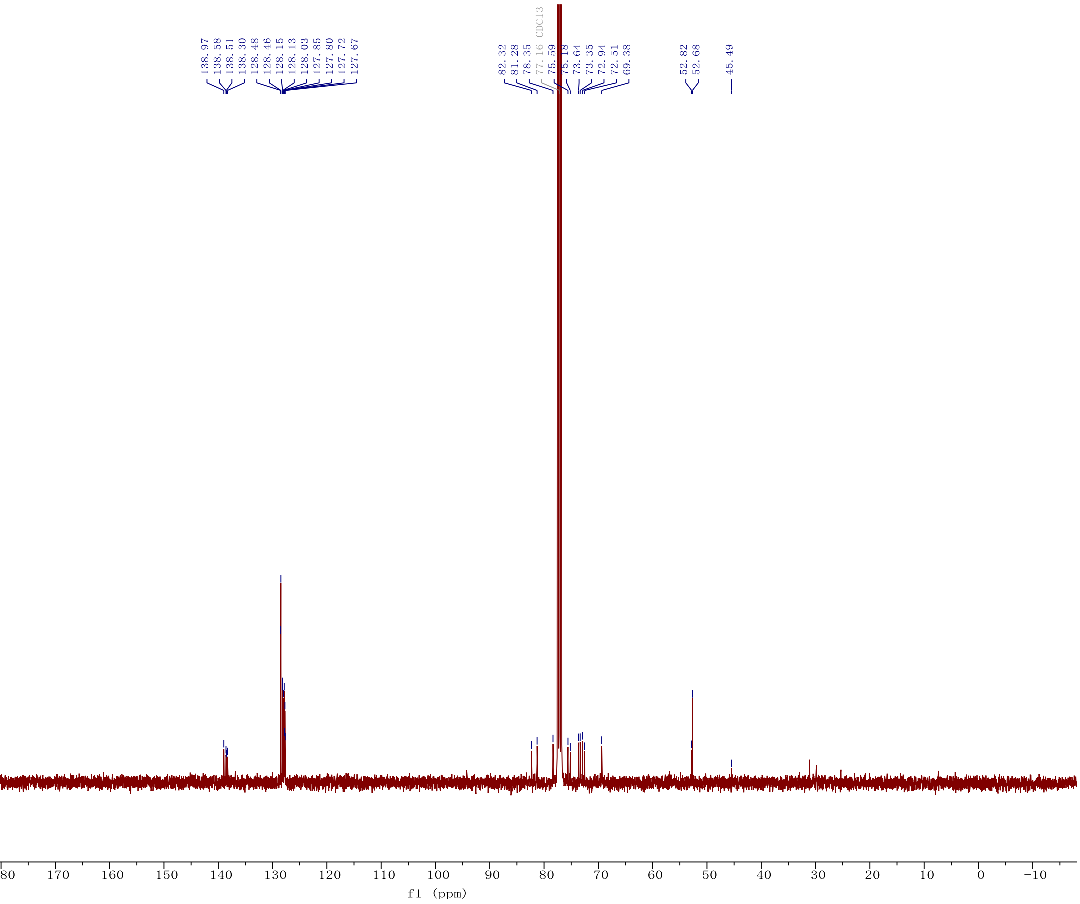

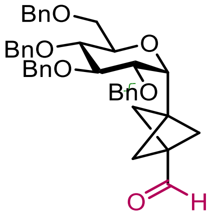

7b

^{1}H NMR

400 MHz, $CDCl_3$

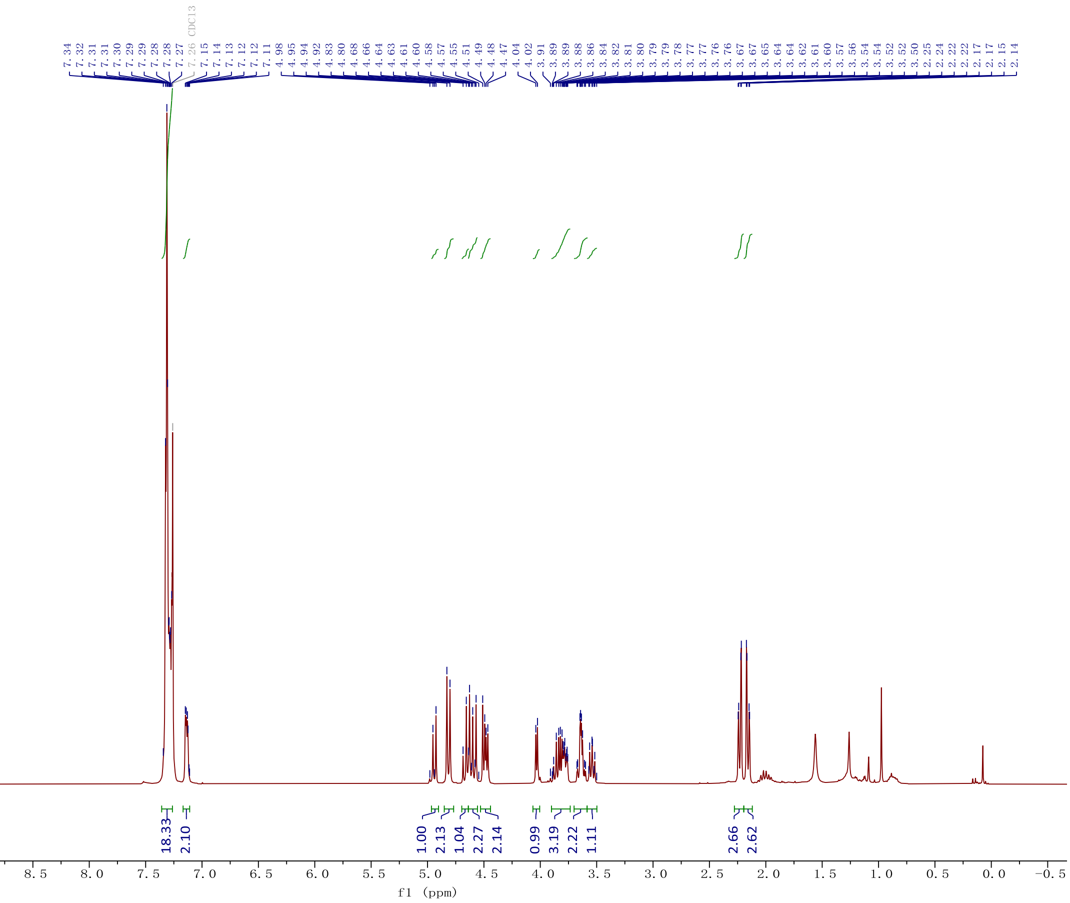

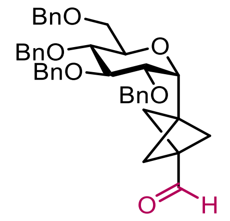

7b

^{13}C NMR

101 MHz, $CDCl_3$

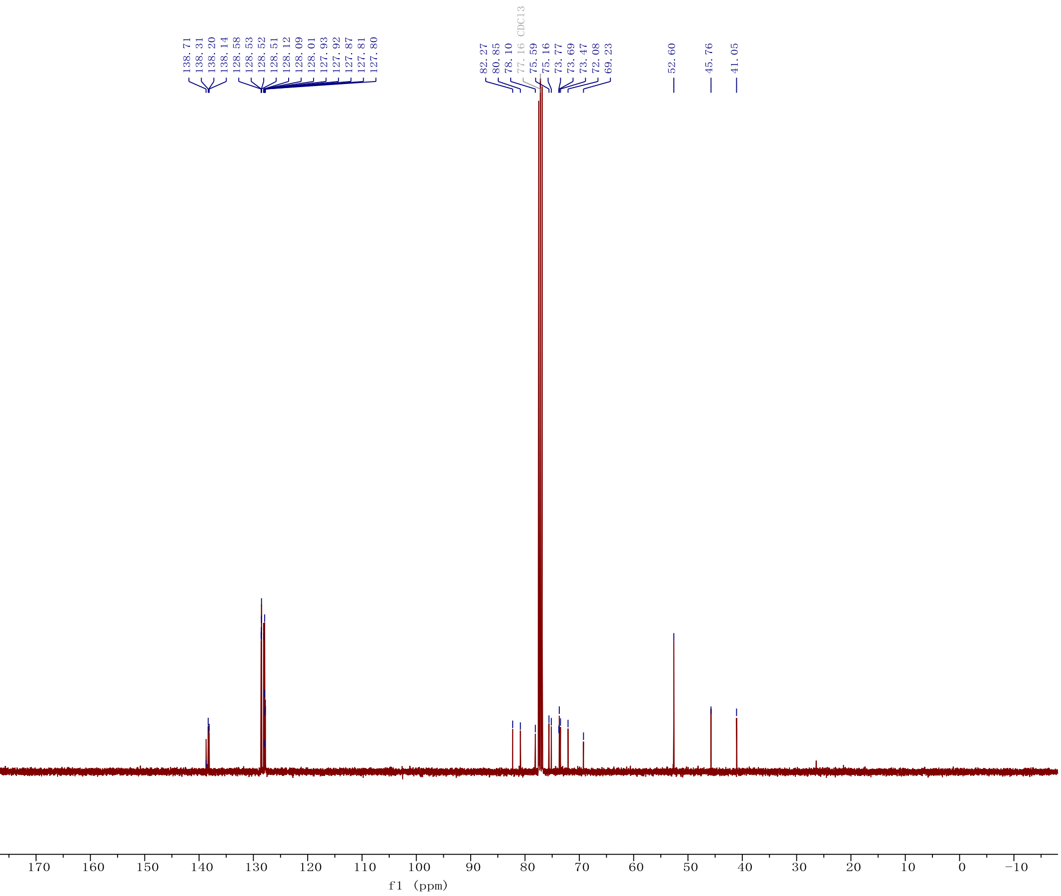

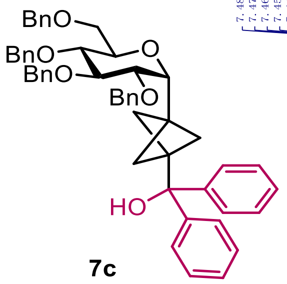

7c

^{1}H NMR

400 MHz, $CDCl_3$

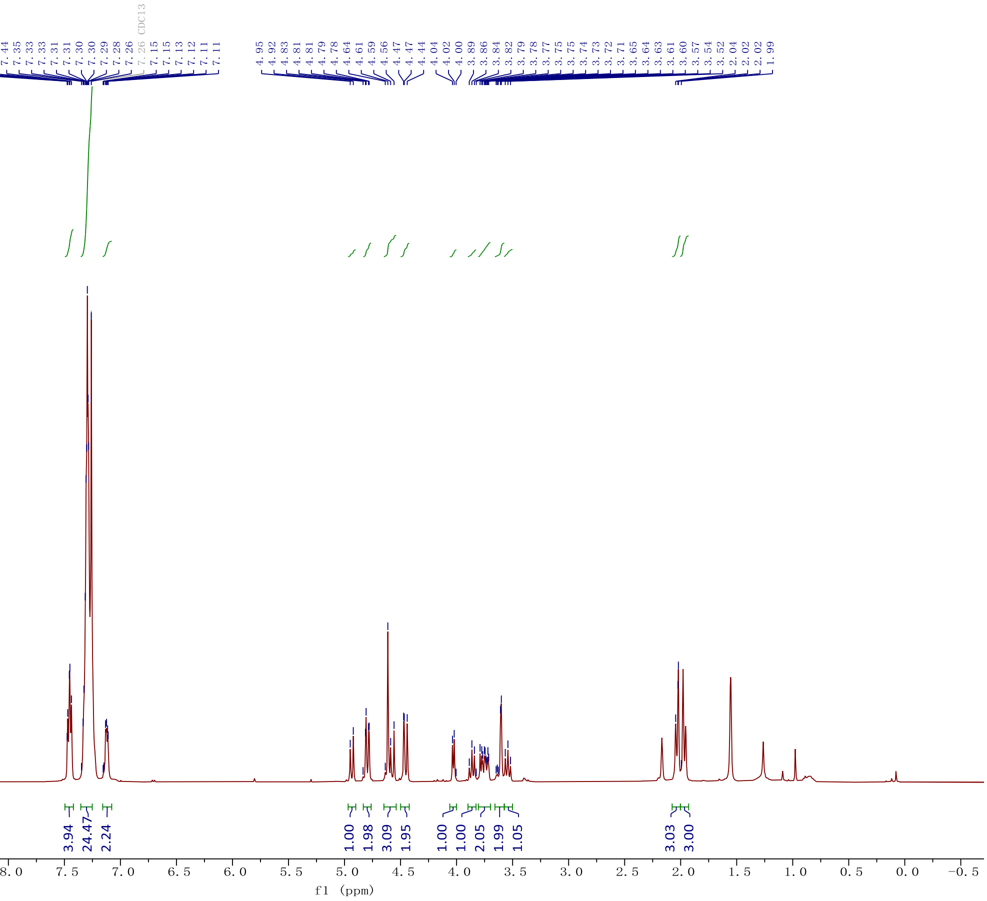

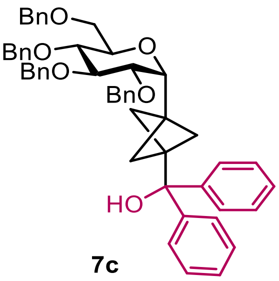

7c

^{13}C NMR

101 MHz, $CDCl_3$

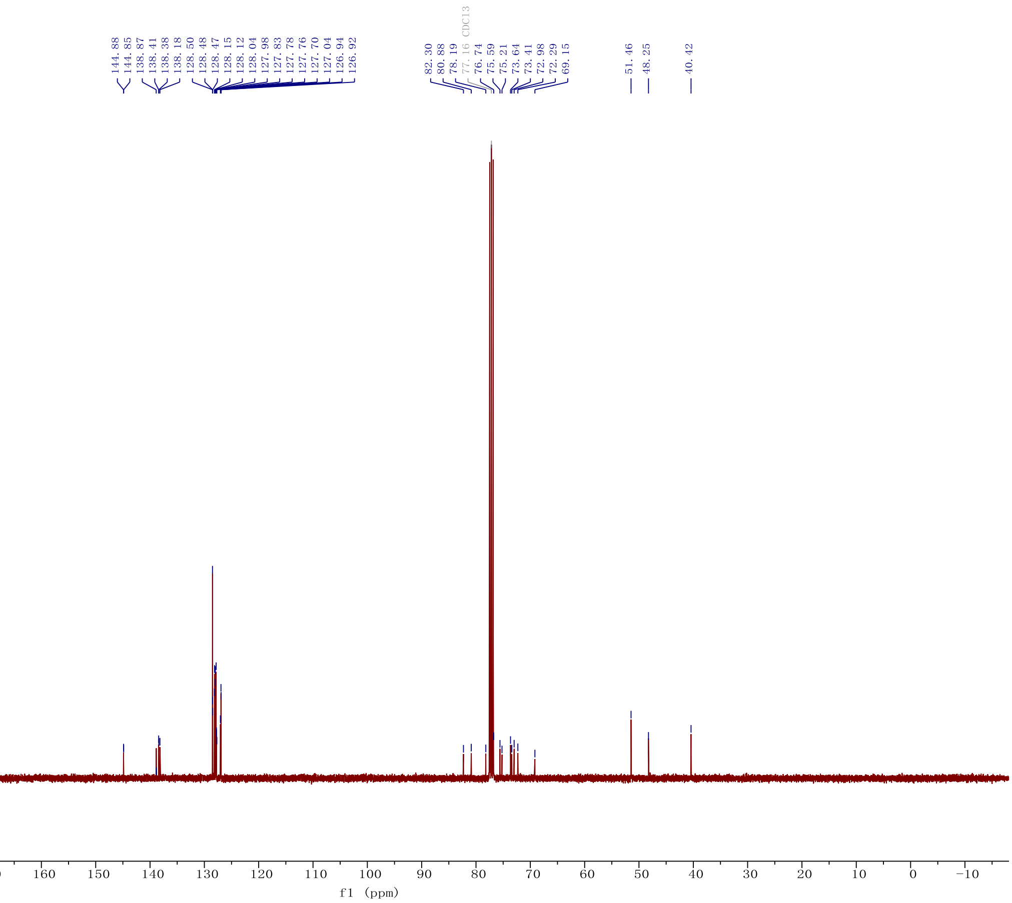

Supplement: Supplementary file 1 [file ol6c02105_si_001.pdf]
